# A Time-Resolved Spectroscopic Investigation of a Novel BODIPY Copolymer and Its Potential Use as a Photosensitiser for Hydrogen Evolution

**DOI:** 10.3389/fchem.2020.584060

**Published:** 2020-10-19

**Authors:** Aoibhín A. Cullen, Katharina Heintz, Laura O'Reilly, Conor Long, Andreas Heise, Robert Murphy, Joshua Karlsson, Elizabeth Gibson, Gregory M. Greetham, Michael Towrie, Mary T. Pryce

**Affiliations:** ^1^School of Chemical Sciences, Dublin City University, Dublin, Ireland; ^2^Department of Chemistry, Royal College of Surgeons in Ireland, Dublin, Ireland; ^3^Energy Materials Laboratory, Department of Chemistry, School of Natural and Environmental Science, Newcastle University, Newcastle upon Tyne, United Kingdom; ^4^Central Laser Facility, Science & Technology Facilities Council, Research Complex at Harwell, Rutherford Appleton Laboratory, Oxford, United Kingdom

**Keywords:** BODIPY polymer, photocatalytic, time-resolved spectroscopy, hydrogen, TAS, TRIR

## Abstract

A novel 4,4-difuoro-4-bora-3a,4a-diaza-s-indacene (BODIPY) copolymer with diethynylbenzene has been synthesised, and its ability to act as a photosensitiser for the photocatalytic generation of hydrogen was investigated by time-resolved spectroscopic techniques spanning the ps- to ns-timescales. Both transient absorption and time-resolved infrared spectroscopy were used to probe the excited state dynamics of this photosensitising unit in a variety of solvents. These studies indicated how environmental factors can influence the photophysics of the BODIPY polymer. A homogeneous photocatalytic hydrogen evolution system has been developed using the BODIPY copolymer and cobaloxime which provides hydrogen evolution rates of 319 μmol h^−1^ g^−1^ after 24 h of visible irradiation.

## Introduction

Increasing levels of CO_2_ in the atmosphere has resulted in an increase in the rate of global warming, necessitating a move away from burning fossil fuels. Hydrogen has been proposed as a clean energy vector, which has led to the development of photocatalysts for hydrogen generation (Dalle et al., 2019; Fajrina and Tahir, 2019). Many inorganic photosensitisers, based on ruthenium, iridium or rhenium, have been used for hydrogen generating systems in both inter- and intra-molecular assemblies. Knowledge of the photophysics of these systems is essential to “fine tune” the photocatalytic systems and increase their efficiencies (Singh Bindra et al., 2012; Tong et al., 2014; Kowacs et al., 2016; Rommel et al., 2016; Das et al., 2017; O'Reilly et al., 2018). However, these systems have many drawbacks including high cost and inefficient use of the visible light spectrum. Organic photosensitisers for hydrogen evolution are less well-developed, despite a number of welcome attributes, such as low cost (Mishra et al., 2009; Manton et al., 2014; Summers et al., 2015; Luo et al., 2018; Lai et al., 2020).

Conjugated polymers offer many advantages over traditional inorganic-based systems for the production of hydrogen. They can facilitate energy transfer along the polymer backbone following photoexcitation, directing it to catalytically active sites for solar-driven hydrogen evolution (Guiglion et al., 2016; Zhang et al., 2016; Wang et al., 2018, 2019; Xu et al., 2018; Dai and Liu, 2020; Jayakumar and Chou, 2020). For instance Zhang et al. reported the use of poly(fluorene-co-phenylene) as a photosensitiser (PS) for hydrogen generation. When used in conjunction with a Ni catalyst and EDTA as a sacrificial agent, efficiencies of up to 429 mmol g^−1^ h^−1^ were achieved (Yong et al., 2018). Graphitic carbon nitride (g-C_3_N_4_) based polymers have also been investigated because of their thermal stability and high hydrogen evolution rates (HER) (Ong et al., 2016; Wang et al., 2017), although they require post-modification, such as surface functionalisation or surface assembly to maximise hydrogen evolution (Ran et al., 2018; Wang et al., 2020; Yi et al., 2020). However, harsh reaction conditions are required to synthesise pristine g-C_3_N_4_ which limits its application as a photocatalyst (Ong et al., 2016).

A diverse range of conjugated polymers can be prepared under mild conditions using metal-catalysed cross-coupling reactions. To date, a range of conjugated polymers including linear polymers, conjugated microporous polymers (CMPs) (Sprick et al., 2016; Liu et al., 2018), covalent organic frameworks (COFs) (Stegbauer et al., 2014; Banerjee et al., 2017; Pachfule et al., 2018), and covalent triazine frameworks (CTFs) have been reported (Bi et al., 2015; Li et al., 2016; Meier et al., 2017). While there are many limitations with polymeric photocatalysts, such as precise control of molecular weight distribution, there are also many advantages, including the ability to establish structure-activity relationships by incorporating different molecular building blocks (Woods et al., 2020). The first report of tunable organic polymers for hydrogen evolution was presented by Cooper et al. who developed pyrene-based conjugated polymers with various monomeric compositions and optical gaps ranging from 1.94 to 2.95 eV (Sprick et al., 2015). The optical gap of the polymer is a key determinant of their efficiency as photocatalysts for hydrogen evolution. For instance, adding various co-monomers in the preparation of poly(*p*-phenylene) enhanced hydrogen evolution. The highest activity was observed with incorporation of a dibenzo[*b, d*] thiophene sulfone moiety, yielding an evolution rate of 92 μmol h^−1^, compared to 2.0 μmol h^−1^ for the homopolymer (Sprick et al., 2016). The superior performance of conjugated polymers and their successful use as photosensitisers has been explained by both their light-harvesting and electron transport capabilities (Chen et al., 2010; Jiang and McNeill, 2017). Certain barriers to improvement of photocatalytic activities or organic polymers remain however. A reduction in exciton binding energies for subsequent charge-carrier generation is required to improve the viability of polymer systems for hydrogen generation.

**Figure 1:**
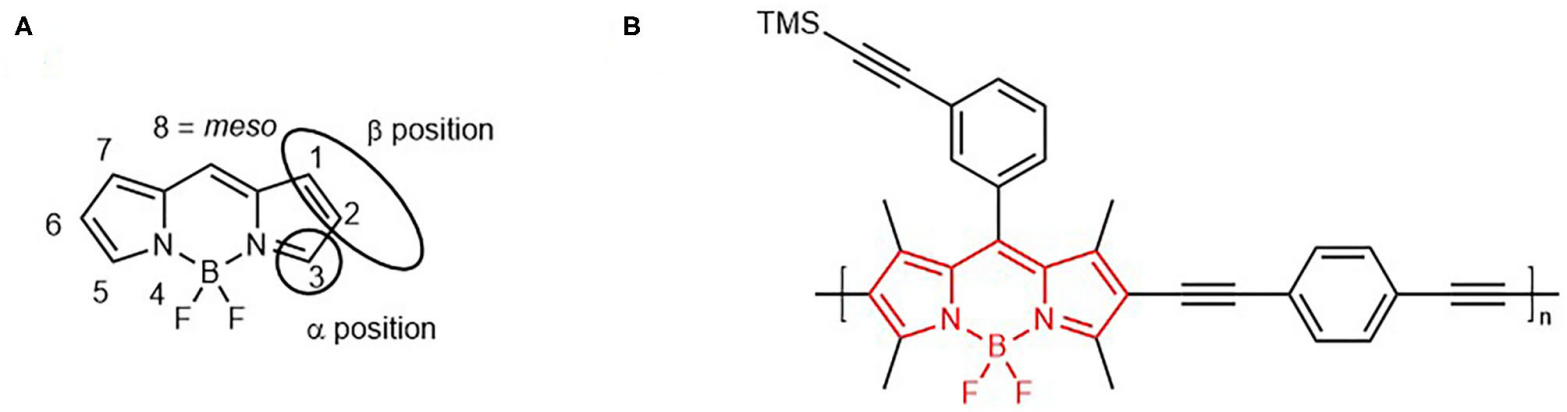
BODIPY core scaffold (4,4-difuoro-4-bora-3a,4a-diaza-s-indacene) showing IUPAC numbering system **(A)**. 3-TMS polymer reported in this study (red bonds showing the BODIPY unit in the polymeric backbone) **(B)**.

BODIPY dyes (Figure 1A) are one of the most extensively studied chromophores in recent years, due to ease of synthesis, thermal stability and solubility in a range of organic solvents (Wan et al., 2003; Azov et al., 2005; Ziessel et al., 2005; Kim et al., 2006; Loudet and Burgess, 2007). A variety of BODIPY architectures have been developed, involving substitution at the *meso* position, the pyrrole unit (β position), the boron atom, as well as post-functionalisation and polymerisation at the periphery of the chromophore (Figure 1B) (Ulrich et al., 2008; Boens et al., 2015, 2019; Zhao et al., 2015; Zhang and Zhu, 2019).

Absorption and emission properties of BODIPYs may vary with substituents on the BODIPY core, e.g., iodine or alkyl groups (Banfi et al., 2013). Synthetic modifications at the *meso* position have less effect on the photophysical properties of the BODIPY however (Guzow et al., 2009; Banfi et al., 2013). Copolymerisation of the BODIPY core at the 2 and 6 position can form linear copolymers (Alemdaroglu et al., 2009; Donuru et al., 2009a,b). BODIPY polymers of this type have applications in areas, such as optoelectronics, organic field transistors, batteries, photovoltaics, and cellular imaging (Squeo et al., 2017), however their use in hydrogen evolution reactions (HER) is the focus of this manuscript. BODIPY monomers and iodinated BODIPY monomers have been used in homogeneous photocatalytic hydrogen evolution, including both intermolecular (Luo et al., 2015b; Sabatini et al., 2016; Dura et al., 2017; Xie et al., 2019) and intramolecular systems (Lazarides et al., 2011; Bartelmess et al., 2014; Luo et al., 2015a; Zheng et al., 2015). Some heterogeneous systems incorporating BODIPY a chromophore onto TiO_2_ surfaces have been shown to lead to H_2_ evolution (Sabatini et al., 2011; Suryani et al., 2019). BODIPY dyes have been immobilised onto photocathodes including NiO (Summers et al., 2015; Black et al., 2017). Limitations in the systems described to date include the narrow absorption profiles of BODIPY chromophores, instability upon irradiation, fast rate of charge recombination and insufficient charge-separated state lifetimes.

Based on the features of the best performing chromophores, we have designed a novel conjugated copolymer containing the BODIPY core unit in the polymer backbone (Figure 1B), which absorb strongly in in the region of the solar irradiance spectrum. Irradiation of this polymer produces a long-lived triplet excited state, which in the presence a cobaloxime molecular catalyst acts as an effective hydrogen evolution catalyst. We also report the first time-resolved study of BODIPY based copolymers using transient absorption and time-resolved infrared spectroscopy to investigate the early-time photodynamics in a range of solvents for comparison with the parent BODIPY monomer.

## Materials and Methods

All solvents were supplied by Aldrich Chemicals Co.® and anhydrous solvents containing sure/seal® were used under the flow of nitrogen. [3-(trimethylsilyl)ethynylbenzaldehyde] was purchased from Sigma-Aldrich and used as received. Reagents were obtained commercially from Aldrich Chemicals Co®., ABCR®, Honeywell Fluka®, Flourochem Ltd.® and were used without any further purification.

### Physical Measurements

^1^H and ^13^C NMR spectra were recorded on either a Bruker 400 or 600 MHz spectrometer and were referenced to the deuterated solvent peak as an internal reference. Mass spectra were measured on a waters Q-TOF 6200 series. All UV spectra were recorded on the Agilent 8453 UV-vis spectrophotometer equipped with Agilent ChemStation software. FTIR measurements were carried out on Perkin-Elmer 2000 FTIR spectrophotometer in a liquid solution cell using spectrophotometric grade dichloromethane. All excitation spectra, emission spectra, emission maps and time-correlated single photon counting (TCSPC) lifetimes were carried out using a FLS1000 photoluminescence spectrometer (Edinburgh instruments), equipped with a Xe Arc lamp for steady-state measurements and a visible PMT-900 detector. All data analysis carried out using Floracle® software. All samples were measured at room temperature and were purged with N_2_ prior analysis. For TCSPC, a 510 nm variable pulse length diode laser (VPL-510) was utilised to excite the ground state sample. The accuracy of the fit of the decays was judged by chi-squared (χ^2^) and sum of residuals was always χ^2^ < 1.1. The fluorescence decay time (τ) was obtained from the slope of the decay curve. Accumulation of the steady-state spectra was obtained using 1 s dwell time and ×2 repeats per spectrum. All samples were measured in a 1 × 1 cm quartz cuvette and samples were <0.2 at 510 nm to ensure an optically dilute solution to avoid inner-filter effects. Size exclusion chromatography (SEC) was used to determine the dispersities (*Ð*_*M*_) and molecular weights of polymers. SEC was conducted in 1,1,1,3,3,3-Hexafluoro-2-propanol (HFiP) using an PSS SECurity SEC system equipped with a PFG 7 μm 8 × 50 mm pre-column, a PSS 100 Å, 7 μm 8 × 300 mm and a PSS 1,000 Å, 7 μm 8 × 300 mm column in series and a differential refractive index (RI) detector at a flow rate of 1.0 mL min^−1^. The systems were calibrated against Agilent Easi-Vial linear poly(methyl methacrylate) (PMMA) standards and analysed by the software package PSS winGPC UniChrom.

### Synthesis

3-TMS BODIPY monomer and 3-TMS diiodo BODIPY monomer were synthesised as per previously reported methods and spectroscopic results are consistent with the literature (Godoy et al., 2010; Li et al., 2019). We report the first synthesis of 3-TMS BODIPY polymer using a previously reported method for the Sonogashira polycondensation reaction (Donuru et al., 2009a). The synthetic procedures used for the synthesis of all compounds are detailed in the Supporting Information.

### Fluorescence Quantum Yield Calculations

Steady-state fluorescence measurements were recorded using the LS50B luminescence spectrophotometer. Prior to obtaining the emission spectra, samples were diluted to ~0.2 abs units at λ_exc_ using the UV-vis spectrometer to inhibit inner-filter effect. The reference compound used was previously reported by Banfi et al. (3-pyridine H-BODIPY, Φ_fl_ = 0.62 in CH_2_Cl_2_) (Banfi et al., 2013). An excitation wavelength of 490 nm and a slit width of 2.5 nm was used for the samples and the standards. The compounds were measured in aerated solution at room temperature. Spectroscopic CH_2_Cl_2_ was the solvent used for all samples and standards. The emission quantum yield was the measured as per the following Equation (1):

(1) $$\begin{array}{r} \Phi_{\text{sample}}=\;\Phi_{\text{std fl}}\times \;\left(\frac{{\text{I}}_{\text{sample}}}{{\text{I}}_{\text{std}}}\right)\times \;\left(\frac{\text{Ab}{\text{s}}_{\text{std}}}{\text{Ab}{\text{s}}_{\text{sample}}}\right) \end{array}$$

Where *I*_*sample*_ and *I*_*standard*_ is the integrated area under the emission curve when the sample was excited at 490 nm. *Abs* denotes the optical density of the sample solution at the excitation wavelength.

### Fluorescence Lifetime Measurements

Fluorescence lifetimes were fitted using Floracle® software to either a simple monoexponential or the following bi-exponential formula (2):

(2) $$\begin{array}{r} \text{Fit}=\text{A}+\;{\text{B}}_{1}{\text{e}}^{\left(-\text{t}/{\text{τ}}_{1}\right)}+{\text{B}}_{2}{\text{e}}^{\left(-\text{t}/{\text{τ}}_{2}\right)} \end{array}$$

where the contribution (% relatively) of each of the different components is B_1_ and B_2_, respectively.

### Singlet Oxygen Quantum Yield Calculations

The singlet oxygen (^1^Δ_g_) quantum yield (Φ_Δ_) was measured using zinc *meso*-tetraphenylporphyrin (ZnTPP) in CHCl_3_ as the standard (Φ_Δ_ = 0.72) (Redmond and Gamlin, 1999). The singlet oxygen near infrared emission (NIR) spectra were recorded using an Andor InGaAs detector coupled with a Shamrock 163 Spectrograph. The excitation sources were supplied by Thorlabs and the monochromatic line used was a 530 nm diode laser. All UV-vis spectra were recorded both before and after singlet oxygen measurements, the optical density of standards and samples were 0.3 absorption units at the excitation wavelength (λ_exc_ = 530 nm). Standard and sample measurements were run under identical experimental conditions employing the same solvent, excitation source, LED exposure time and accumulation cycles to allow for direct comparison of NIR emission spectra. The NIR emission spectra was then integrated using baseline correction software in the region of λ_1_ = 1,230 nm to λ_2_ = 1,330 nm and Φ_Δ_ calculated using the following Equation (3):

(3) $$\begin{array}{r} \Phi_{\text{sample}}=\;\frac{\Phi_{\text{ref}}\left(\text{Are}{\text{a}}_{\text{sample}}\text{xAb}{\text{s}}_{\text{ref}}\right)}{\left(\text{Are}{\text{a}}_{\text{ref}}\text{xAb}{\text{s}}_{\text{sample}}\right)} \end{array}$$

Where Φ_ref_ is the singlet oxygen quantum yield of the standard, Area_sample_ and Area_ref_ are the integrated area between 1,230 and 1,330 nm of the phosphorescence of singlet oxygen, respectively, Abs_ref_ and Abs_sample_ are the absorption of both solutions at the wavelength of excitation.

### ps-Transient Absorption Measurements and ps-Time Resolved Infrared Measurements

ps-TA and ps-TRIR spectra were recorded using the ULTRA instrument at the Central Laser Facility in the Rutherford Appleton Laboratory in the U.K. and has been described elsewhere (Greetham et al., 2010).

### ns-Transient Absorption Measurements

ns transient absorption data were recorded on the LP980 transient absorption spectrometer (Edinburgh Instruments), λ_exc_ = 355 nm. All samples were degassed using three freeze-pump thaw cycles prior to sample measurement. The optical density of the sample was ~0.3 at 355 nm prior to transient absorption measurement on the LP980. Samples were checked for photodegradation by comparing UV-vis absorbance spectra before and after TA measurements and no changes were observed.

### Photocatalytic Homogeneous H_2_ Evolution Experiments

For photocatalytic hydrogen evolution, each sample was prepared in a 23 mL glass Schlenk tube stoppered with an air-tight rubber septum. Prior to sample preparation, an aqueous solution of 0.2 M ascorbic acid was adjusted to the desired pH value by titrating the solution with an appropriate amount of 0.2 M NaOH solution. The polymer and the catalyst were dissolved in 4 mL of organic solvent (either CH_3_CN or THF) followed by addition of 4 mL of the ascorbic acid solution to yield an 8 mL 1:1 (v/v) mixture of organic solvent and aqueous ascorbic acid solution (0.1 M final concentration of ascorbic acid). The components were dissolved in the photocatalytic solution, and degassed using three freeze-pump thaw cycles prior to irradiation. The solution based photocatalytic experiments (λ > 420 nm) were all conducted for a period of 24 h using a 300 W Xe arc lamp. All experiments for the detection of hydrogen evolution were carried out in triplicate. At each timepoint reported, 1 mL of the headspace from the Schlenk flask was injected onto the GC to quantify the amount of H_2_ produced. For the heterogeneous photocatalysis on NiO, the reaction cell was degassed for at least 15 min with either nitrogen or argon. Aqueous electrolytes were freshly prepared and pH adjusted with concentrated HCl, and measured with a benchtop pH meter (Hanna Instruments). The cell was irradiated with simulated 1 sun intensity light (AM 1.5, 100 mW cm^−2^) using a 300 W Xe arc lamp (Oriel Instruments). Hydrogen was detected with a thermal conductivity detector (TCD) with the system operating at 80°C. Gas sampling was done in flow, through an integrated cell block (Supplementary Figure 44). Further details can be found in Supporting Information.

## Results and Discussions

### Synthesis

**Figure 2:**
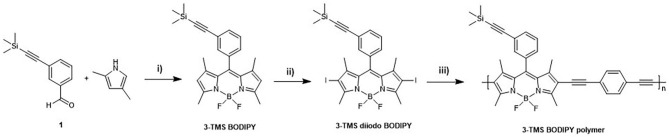
Synthesis of 3-TMS polymer. *Reagents and conditions*: **i)** TFA, DDQ, BF_3_OEt_2_, TEA, dry CH_2_Cl_2_, N_2_, RT, 3 days; **ii)** I_2_, HIO_3_, EtOH, r.t. overnight; **iii)** Pd(PPh_3_)_2_Cl_2_, PPh_3_, CuI, THF/Diisopropylamine, reflux, 3 days.

The synthesis of the BODIPY polymer was achieved in three steps (Figure 2), via initial synthesis of the BODIPY monomer (Banfi et al., 2013) and subsequent iodination at the 2 and 6 position (3-TMS diiodo BODIPY). The polymer (M_n_ = 7.5 kDa, Ð* = 1.4, see Supplementary Figure 8) was synthesised using Sonogashira polycondensation between the iodinated BODIPY core and a 1,4-diethynylbenzyl linker to yield the 3-TMS BODIPY polymer depicted in Figure 2 (herein referred to as polymer). The resulting polymeric material exhibits a bimodal molecular weight distribution. SEC analysis confirmed the successful formation of polymeric structures well-beyond the oligomer regime with a bimodal molecular weight distribution (Supplementary Figure 8).

### UV-Visible and Emission Spectroscopy

**Figure 3:**
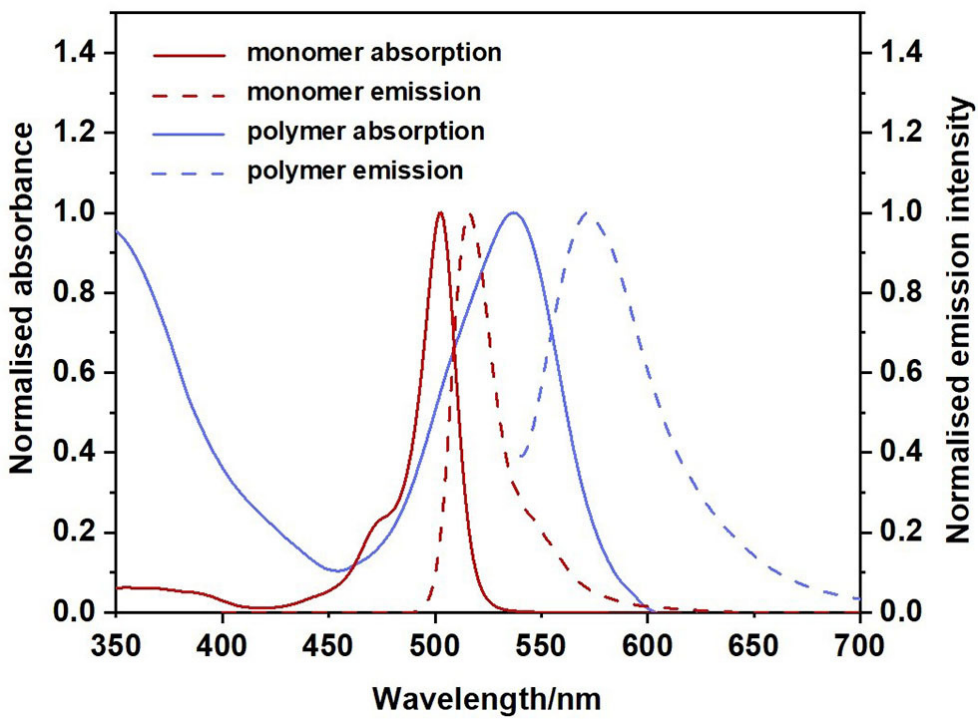
Normalised absorption spectra for monomer (solid red), polymer (solid blue) and normalised emission spectra for monomer (dashed red) and polymer (dashed blue). CH_2_Cl_2_, 298 K.

The UV-visible and emission spectra of the monomer and polymer are presented in Figure 3 (recorded in CH_2_Cl_2_). The spectrum indicated with a solid red line corresponds to the absorption spectrum of the monomer. This spectrum displays the characteristic narrow absorption band of the BODIPY core (λ_max_ = 503 nm; FWHM_abs_ = 768 cm^−1^) which has been assigned to a S_0_ to S_1_ π-π^*^ transition. A weak shoulder is observed at 470 nm attributed to the S_0_-S_2_ (π-π^*^ transition). The associated emission spectrum (dashed red line) also displays the characteristic sharp band, λ_max_, at 516 nm representing a Stokes shift of 501 cm^−1^, within the range typical for BODIPY materials. This indicates only a small change in dipole moment (or geometry) in transition from the ground to the excited state (Luo et al., 2015b).

The fluorescence quantum yield for the monomer is high (Φ_fl_ = 92% Table 1), in agreement with the literature (Ulrich et al., 2008; Zhang and Zhu, 2019). The UV spectrum of the polymer is presented as the solid blue feature in Figure 3 (with additional spectra in various solvents presented in Supplementary Figure 11). The increased π-conjugation along the polymer backbone also results in a broadening of the BODIPY absorption (Alemdaroglu et al., 2009; Meng et al., 2009; Khetubol et al., 2015). The lowest energy absorption feature of the polymer is broad (FWHM_abs_ = 2,209 cm^−1^), with the λ_max_ is red-shifted by 36 nm compared to the monomer. A new intense feature is present at about 350 nm, which is also displayed in the excitation spectrum of the polymer (Supplementary Figure 13). In addition, the lowest energy absorption band is partially resolved into two features in some solvents, indicating the existence of different environments for the BODIPY moiety along the polymer backbone. In this regard, the BODIPY unit is acting as a probe to the various environments on the polymer, and these environments have a dramatic effect on the photophysical behaviour of the BODIPY chromophore. The emission spectrum of the polymer is also broadened compared to the monomer with a larger Stokes shift of 1,101 cm^−1^ (Table 1). The emission maximum of the polymer is shifted some 57 nm to the red compared to the monomer and the emission band extends to 700 nm. These observations are consistent with other studies on polymeric BODIPY systems and again implies the presence of a multiple of environments for the BODIPY chromophore (Bucher et al., 2017). The fluorescence quantum yield of the polymer is greatly reduced compared to the monomer (Φ_fl_ = 0.09, Table 1). It appears that incorporation of a conjugated linker unit is important in modulating the photophysical properties of BODIPY polymers and reducing the luminescence of the BODIPY core (Alemdaroglu et al., 2009).

**Table 1:** Photophysical properties of the monomer and polymer.

**Compound**	**λ_abs_ (nm)**	**λ_em_ (nm)^a^**	**Stokes shift Δν (cm^**−1**^)^b^**	**Optical gap (eV)^c^**	$\Phi_{\text{fl}}^{\text{d}}$	$\Phi_{\text{ISC}}^{\text{e}}$	$\Phi_{\Delta }^{\text{f}}$	**τ_s_ (ns)^g^**	**τ_T_ (μs)^h^**
Monomer	503	516	501	2.41	0.92	0.08	0.05	4.3	–
Polymer	539	572	1101	2.19	0.09	0.91	0.77	1.1, 3.9	61

*All spectra were recorded in CH_2_Cl_2_ at room temperature*.

a *Wavelength used to excite the compound correlated to the absorption maxima of each compound, respectively*.

b *Stokes shift*.

c *Calculated from the onset of absorption spectrum (Wanwong et al., 2016) (Supplementary Figure 30)*.

d *Flourescence quantum yield calculated using 3-pyridine H-BODIPY as a reference standard, where Φ_fl_ = 62% in CH_2_Cl_2_ (Banfi et al., 2013), detailed in the experimental section*.

e *Intersystem crossing efficiency, approximated by the Ermolev's rule: Φ_ISC_ = 1 – Φ_fl_ (Wu et al., 2011)*.

f *Singlet oxygen quantum yield*.

g *Obtained from TCSPC technique in CH_2_Cl_2_*.

h *Obtained from transient absorption spectroscopy, λ_exc_ 355 nm, sample degassed using freeze-pump thaw method*.

**Figure 4:**
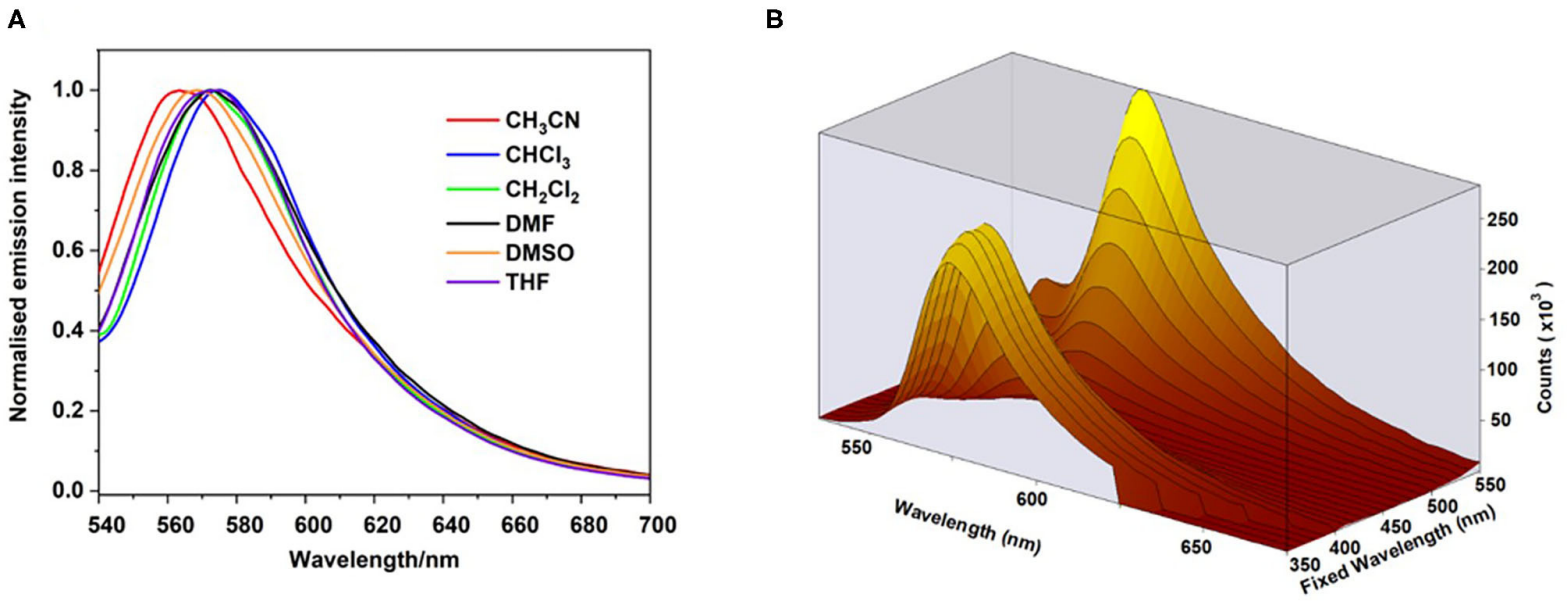
Emission spectra of the polymer in acetonitrile (red), chloroform (blue), dichloromethane (green), dimethylformamide (black), dimethyl sulfoxide (orange) and tetrahydrofuran (purple) following excitation at 530 nm **(A)**. 3-D Emission map of polymer in dichloromethane, recorded at room temperature. Fixed wavelength axis indicates the various excitation wavelengths used **(B)**. All spectra recorded in at room temperature.

The emission spectra of the polymer were relatively insensitive to the nature of the solvent (Figure 4A) implying that BODIPY emission occurs from only selective environments. However, a slight hypsochromic shift (*ca*.−9 nm) is observed in acetonitrile compared to other solvents (571–574 nm, Table 2). The emission map (Figure 4B) follows closely the absorption profile of the ground state, indicating coupling of the chromophores responsible for the two lowest energy features in the absorption spectrum to the emissive state.

**Table 2:** Fluorescence decay lifetimes of the monomer and polymer using 510 nm LED diode.

**Compound**	**Solvent**	**$\lambda_{\text{em}}^{\text{a}}$ (nm)**	**$\tau_{1}^{\text{b}}$ (ns)**	**% rel τ_1_**	**$\tau_{2}^{\text{b}}$ (ns)**	**% rel τ_2_**
Monomer	CH_2_Cl_2_	513	–	–	4.3	100
Polymer	CH_2_Cl_2_	572	1.1	8.7	3.9	91.3
	THF	573	0.8	9.4	3.8	90.6
	CHCl_3_	574	0.7	7.4	4.2	92.6
	CH_3_CN	568	0.8	14.5	3.6	85.5
	DMSO	570	0.8	25.6	3.5	74.4
	DMF	572	0.4	27.8	3.8	72.2

a *λ_em_ (nm): emission wavelength (at the maximum intensity)*.

b *τ_fl_ (ns): fluorescence lifetimes*.

### Fluorescence Lifetime Analysis Using TCSPC

**Figure 5:**
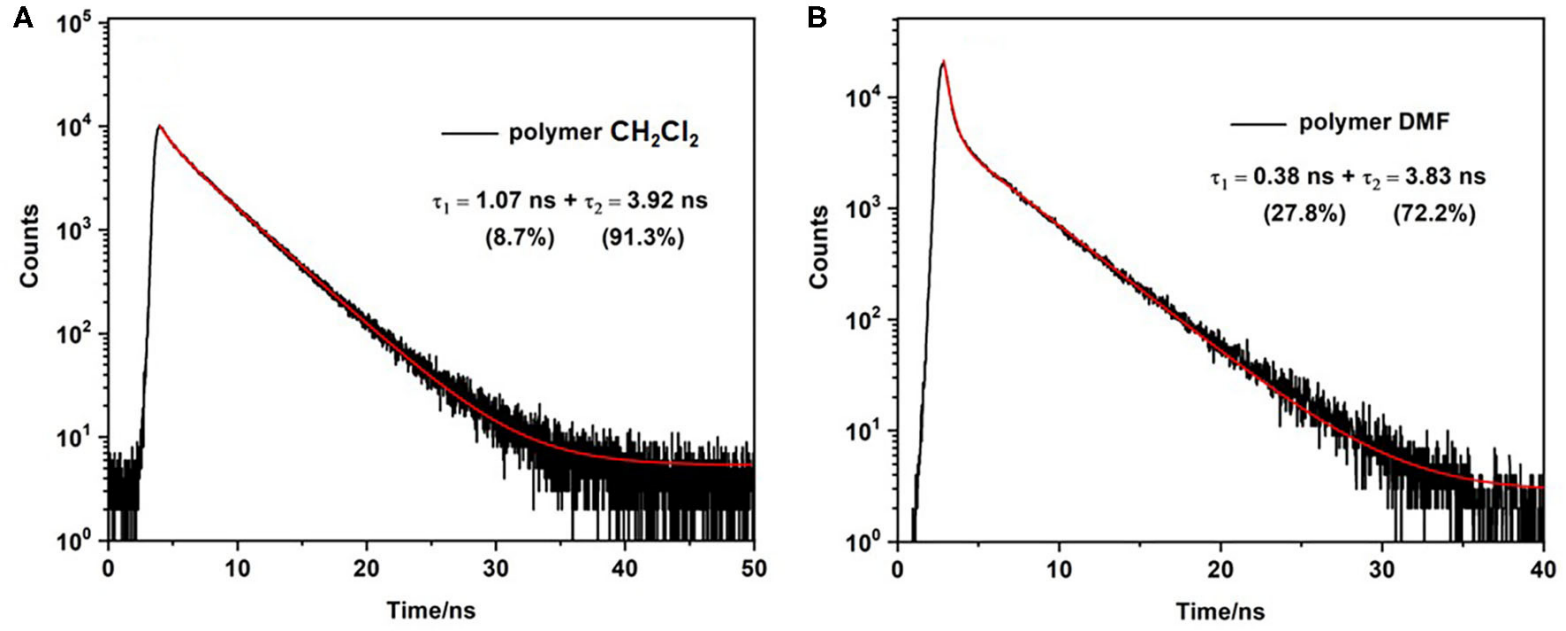
Emission decay of polymer in CH_2_Cl_2_ **(A)** and DMF **(B)** obtained using FLS1000 Photoluminescence spectrometer λ_exc_ = 510 nm. All solutions purged with N_2_ for 20 minutes prior to sample measurement.

The fluorescence lifetime (τ_fl_) of the monomer was measured in dichloromethane solution. The luminescence decay follows a single exponential profile yielding a τ_fl_ of 4.3 ns (Supplementary Figure 25), typical of emission from the lowest energy singlet excited state (Krumova and Cosa, 2010). As mentioned above the quantum yield for fluorescence is much lower for the polymer and the fluorescence decay shows a biexponential profile, comprising a fast process with τ_fl_ on the order of 1 ns or less, and a slower process with a lifetime similar to that of the singlet emission from the monomer (τ_fl_ ≈ 4 ns). The lifetimes were measured in a variety of solvents and these data are presented in Table 2. It is clear that while the lifetimes of the two decay processes are largely insensitive to the solvent, however the relative proportions of the two decay processes vary. The fast process contributes more in DMF or DMSO than it does in CH_2_Cl_2_ or THF (Figure 5). These observations can be explained by proposing that the singlet emission only occurs from selective environments on the polymer, which do not facilitate intersystem crossing (ISC) to the triplet surface (Chen et al., 2019). The proportion of the excited BODIPY centres, which undergo singlet emission, is strongly affected by the interaction between the polymer and the solvent. This explains the substantial decrease in Φ_fl_ for the polymer compared to the monomer. The majority of excited BODIPY centres can undergo charge transfer processes along the conjugated polymer which promotes ISC (Filatov, 2019). More details of the emission decay profiles are presented in the supporting information (Supplementary Figures 26–29).

Biexponential decays have previously been reported for other BODIPY polymers and are consistent with the results in this study (Economopoulos et al., 2013). For instance Donuru et al. (2009b), reported a lifetime of τ = 4.1 ns for a BODIPY monomer, and a lifetime of τ = 1.1 ns in the resulting polymer. Other reports also follow similar trends of a decreased lifetimes for polymeric materials (Donuru et al., 2010). Douhal et al. reported three lifetime components for another conjugated BODIPY copolymer, τ_1_ = 0.22 ns, τ_2_= 0.7 ns, and τ_3_ = 4.3 ns in CH_2_Cl_2_, λ_exc_ = 371 nm (Piatkowski et al., 2019). The latter two lifetimes are similar to those obtained in this work (Table 2) and it is possible that a third lifetime component exists for our polymer but on a timescale shorter than the instrument response time of 50 ps (see the transient absorption results outlined below).

### Transient Absorption Spectroscopy

**Figure 6:**
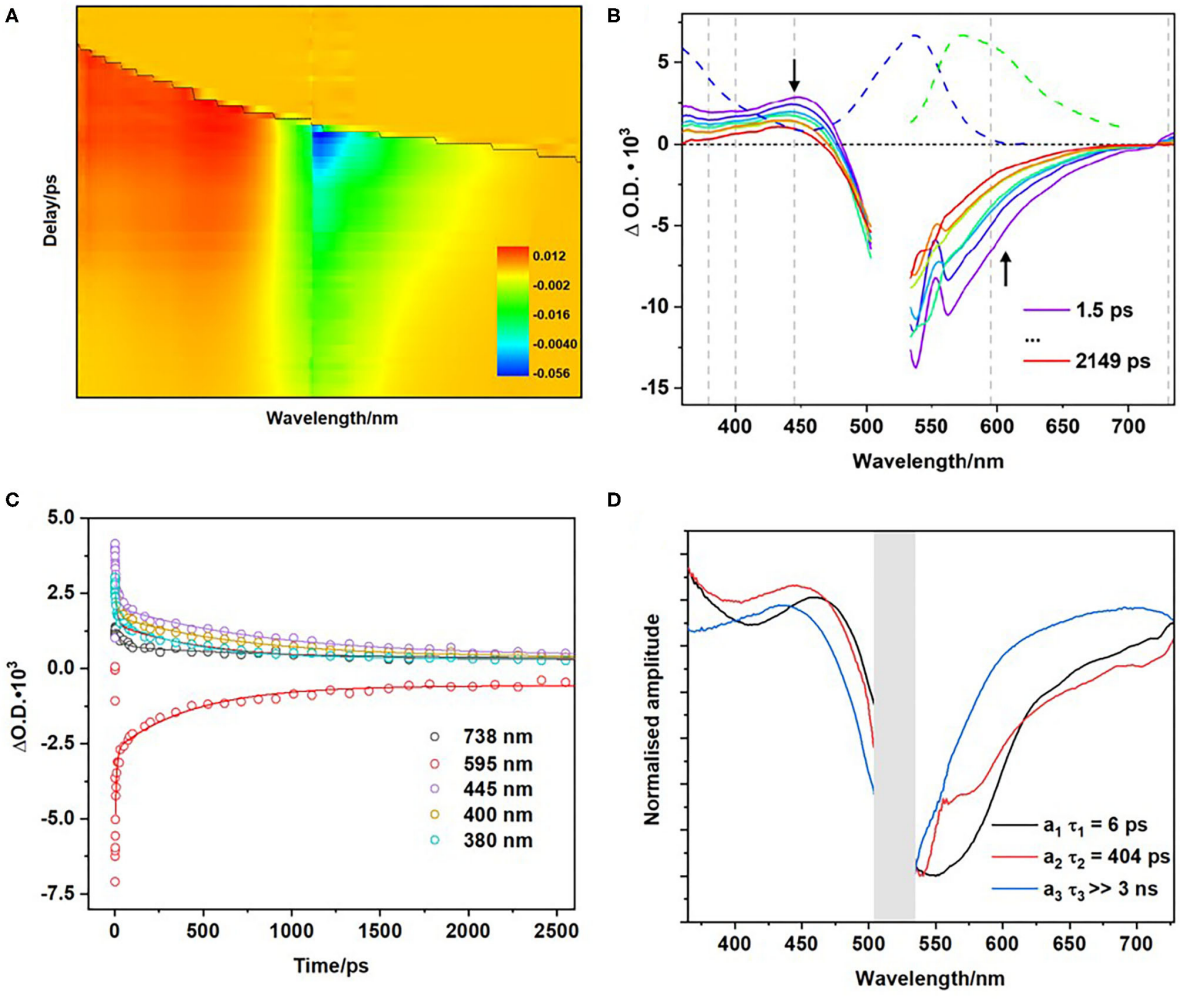
Transient absorption spectra of polymer in CD_3_CN following excitation using 525 nm **(A)**. Transient absorption spectra of polymer corresponding to indicated time delays, grey lines indicate kinetic traces analysed **(B)**. Temporal evolution of the spectra at wavelengths indicated by grey broken lines in **(B)** together with the exponential best fit line **(C)**. Normalised Decay Associated Spectra (DAS) corresponding to the lifetimes extracted from modeling of the TA spectra in CD_3_CN **(D)**.

To investigate the early excited state dynamics, ps-ns transient absorption (TA) measurements were undertaken on the polymer in a range of solvents including CD_3_CN, DMSO, CHCl_3_, and CH_2_Cl_2_. Figure 6A displays the colour 2-D TA plot for the polymer following excitation at 525 nm in CD_3_CN and Figure 6B contains the corresponding time-resolved spectra. To aid in spectral interpretation the ground state absorption spectrum of the polymer (blue dashed line) and emission spectra (green dashed line) are plotted. The region of most interest is indicated with a downward arrow, while the region close to the upward arrow is subject to distortions because of emission.

Excitation results in a ground state bleach (GSB) around 540 nm and the formation of an excited state absorption (ESA) at ~435 nm (Figure 6B). The ESA at 435 nm decays with three decay components (Figure 6B) with τ_1_ = 6 ± 1 ps, and τ_2_ = 404 ± 32 ps, and a third component with τ_3_ > 3 ns (Table 3). Supplementary Figure 32 shows the additional TA spectra obtained in DMSO, CHCl_3_, and CH_2_Cl_2_, all of which exhibit similar spectral features. The longer of these lifetimes are similar to those obtained in the emission studies described above (Table 2). The first component (τ_1_), with lifetimes in the range 6–18 ps, is too short-lived to be detected in the luminescence studies. The time scale for this species is within the range of vibrational relaxation, however the spectroscopic behavior of the bands is not typical of this process, and another possibility is structural relaxation of the BODIPY polymer (Kee et al., 2005; Suhina et al., 2017). The second component (τ_2_) in the range 400–1,400 ps corresponds to the fast component in the emission lifetime studies. The more rapid excited state decay in the more polar solvents suggests that the excited state is quenched by electron transfer to form a charge-separated triplet state, which is facilitated by polymer-solvent interactions. The absorption of the triplet species persisted to >3 ns after excitation, as did the GSB. Triplet excited state formation is also observed in other heavy-atom-free BODIPY photosensitisers (Filatov, 2019). Halogenated BODIPY monomers also have an ESA feature in this region attributed to the formation of a triplet excited state (Sabatini et al., 2011; Lee et al., 2020).

**Table 3:** Summary of the lifetimes in the TAS experiments for the ESA feature at ~445 nm (using a biexponential function) following excitation at 525 nm.

**Solvent**	**τ_1_ (ps)**	**τ_2_ (ps)**
CD_3_CN	6 ± 1	404 ± 32
DMSO	18 ± 3	522 ± 56
CHCl_3_	15 ± 2	784 ± 56
CD_2_Cl_2_	9 ± 1	1359 ± 291

Figure 6D displays the decay associated spectra (DAS) for the polymer in CD_3_CN. The DAS spectra exhibit the amplitude (a_i_) of each lifetime component (τ_i_) along the absorption spectral window in the experimental set-up. The τ_1_ DAS is dominated by a positive feature at 464 nm and, a negative feature corresponding to the GSB at ~546 nm. This initial species absorbs to the red of the parent and of the second species populated. The τ_2_ DAS is structurally similar showing a slight blue shift of the ESA feature on the higher energy side of the spectra *ca*.−18 nm. Finally, a positive feature is also present in the τ_3_ DAS centered at 455 nm and persists on the ns-timescale. Consequently, we can assign the long-time life component to the absorption of a triplet state, which is further supported by ns-TA experiments.

For comparison, TAS was also carried out with the parent BODIPY monomer and the diiodo monomer in CD_3_CN, following excitation at 525 nm (Supplementary Figures 34, 35). The lifetime obtained for the BODIPY monomer (τ ~ 4 ns), is consistent with the population of the S_1_ state, followed by relaxation to the ground state. The GSB feature of the diiodo BODIPY monomer is long-lived and did not decay within the timeframe of the experiment. In the TA spectra for the diiodo BODIPY dye concurrent with decay of the band initially observed at 460 nm, a further band grows in at ~430 nm over *ca*. 50 ps (that is absent in the BODIPY monomer) that persists beyond the experimental times used (2 ns). This is attributed to ISC with formation of the triplet state which is characteristic of heavy-atom BODIPY analogues (Sabatini et al., 2011).

**Figure 7:**
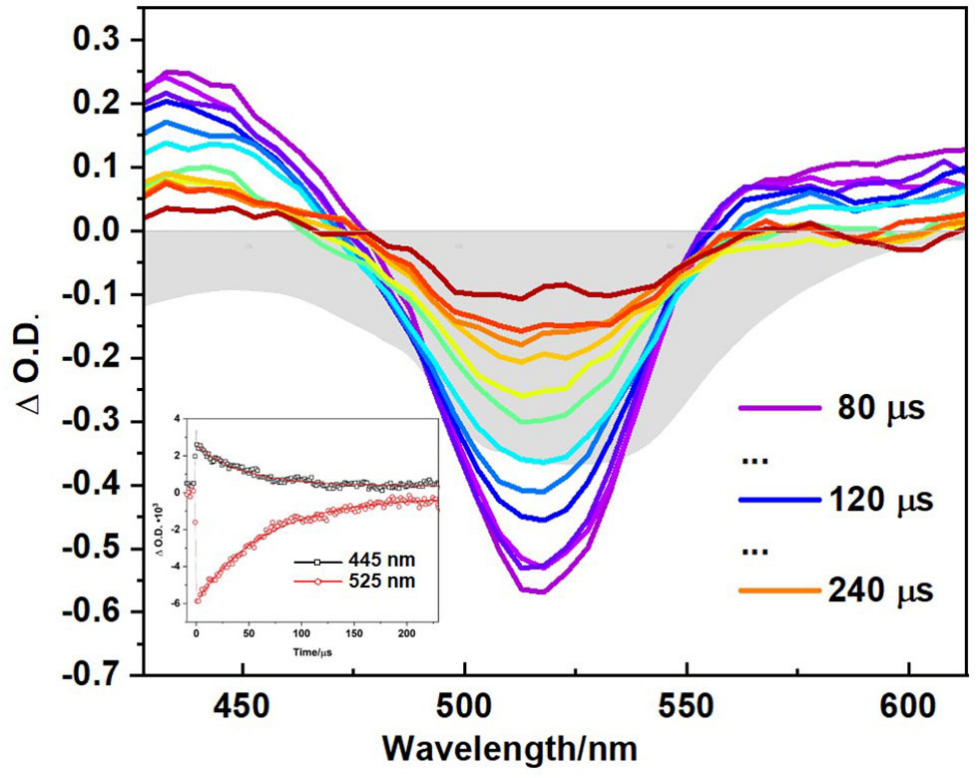
Transient absorption spectroscopy of polymer in CH_3_CN, shown at different time delays, (λ_exc_ = 355 nm) and corresponding decay at ESA and GSB shown in inset at stated wavelength: 445 nm (black squares) and 525 nm (red circles) with red line showing monoexponential fitting to obtain triplet lifetime. Grey shaded curve represents the ground state absorption spectra of polymer in same solvent. All samples were prepared using freeze-pump thawed to degas.

Time-resolved experiments on the polymer in CH_3_CN, using ns laser pulses confirmed the formation of a long-lived excited state consistent with it being a triplet species with τ_T_ = 60 μs. The ns-TA spectrum (Figure 7) exhibits a broad GSB in addition to two ESA features at ~435 and 585 nm, which resembles the final spectrum obtained in the ps-TA experiments described above. The triplet lifetime of 60 μs is similar to triplet state lifetimes measured for other conjugated polymers containing thiophene and fluorene moieties previously measured (20–120 μs) (Yong et al., 2018). Lifetimes in the range 450–710 μs have been reported for polymers with pendant iodo-substituted BODIPY units (Zhang et al., 2020). Triplet excited state species have also been observed for a BODIPY polymer containing an ethynyl thiophene linker (Bucher et al., 2017). The singlet oxygen quantum yield for the polymer (Φ_Δ_ = 0.77, Table 1) represents more than a 10-fold increase over the singlet oxygen quantum yield of the monomer (Φ_Δ_ = 0.05, Table 1) (Supplementary Figure 24) (DeRosa and Crutchley, 2002; McDonnell et al., 2005).

### Time Resolved Infrared Spectroscopy

Time resolved infrared spectroscopy (TRIR) was also carried out on the polymer on the ps to ns timescales following excitation at 525 nm (in a range of solvents; DMSO, CD_3_CN, CHCl_3_, and CD_2_Cl_2_). A representative FTIR spectrum for the polymer is displayed in Supplementary Figure 9, in conjunction with 1,4-diethynylbenzene which was used in the synthesis of the polymer.

**Figure 8:**
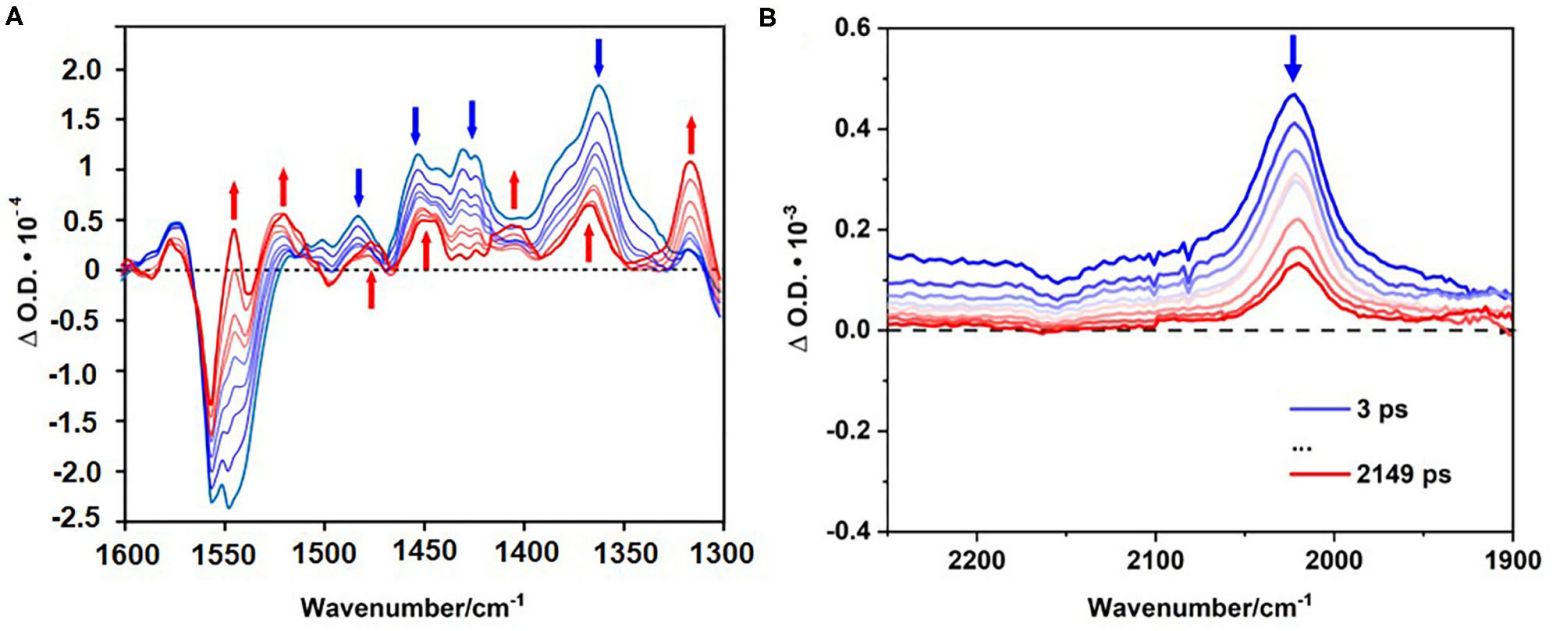
TRIR spectra following excitation at 525 nm of the polymer in CD_3_CN (left) in the fingerprint region **(A)**. TRIR spectra following excitation at 525 nm of the polymer dissolved in chloroform the triple bond region **(B)**. Blue spectra indicating initial time delays (ps), red spectra indicated final time delays (ps).

Immediately following excitation, a depletion is evident at ~1547 cm^−1^ together with new bands at 1267, 1362, and 1452 (broad) cm^−1^ which are assigned to a singlet excited state of the BODIPY. Previously electron transfer dynamics for BODIPY chromophores in CH_2_Cl_2_ have been reported, with a GSB at 1543 cm^−1^, and ESA features at 1339, 1297, and 1507 cm^−1^, respectively (Schoder et al., 2017). These spectroscopic features produced within the instrument response time in our studies, have identical lifetimes [τ = 25 (±5) ps] and are assigned to a singlet excited state (Black et al., 2017). However, for the polymer in this study, additional positive features develop, principally that at 1316 cm^−1^ which is fully formed within 1 ns and persists on the ns-timescale. We have assigned these features to a triplet excited state. Because of the substantial band overlap, accurate kinetic data could not be obtained for the growth of the triplet species, however it is clear that it forms at a rate similar to the fast decay component in the emission lifetime measurements Figure 8A.

Figure 8B displays the spectra obtained in the region where carbon-carbon triple bonds absorb. Following excitation of the polymer in CHCl_3_ (FTIR, Supplementary Figure 9) a positive feature was produced at 2,023 cm^−1^. The equivalent feature for the ground state polymer is observed at 2,339 cm^−1^. The substantial shift of this feature points to charge transfer from the triplet bond to the BODIPY unit in the excited state (Zhu et al., 2008, 2017; Bandyopadhyay et al., 2016). Kinetic analysis of the signal at 2,023 cm^−1^ reveals two decay lifetimes, τ_1_ = 13 ± 2 ps and τ_2_ = 757 ± 62 ps corresponding to vibrational relaxation, and ISC to the triplet surface, respectively. These lifetimes are consistent with those obtained in the TA experiments. Following ISC, a product feature persists to the ns timescale as expected for a triplet species.

TRIR studies were also performed using the diiodo BODIPY monomer. Following excitation, the diiodo monomer displayed a bleach at 1538 cm^−1^ and positive bands at 1488 and 1442 (broad) cm^−1^ (Supplementary Figure 38). After *ca*. 30 ps, two additional bands form at 1525 and 1370 cm^−1^, respectively and persist beyond the timeframe of the experiment. TRIR spectra of the BODIPY monomer under identical experimental conditions reveal a monoexponential decay of the band at 1540 cm^−1^, characteristic of BODIPY monomers. This indicates the decay of the singlet excited state, with no evidence for features which could be assigned to a triplet species (Supplementary Figure 39). TRIR spectra of the monomer and diiodo monomer in the carbon-carbon triple bond region were, as expected, featureless (Supplementary Figure 40).

### Photocatalytic Hydrogen Generation Studies

#### Solution Studies

The polymer was assessed for hydrogen generation with cobaloxime as the catalyst, under both basic and acidic conditions. No hydrogen evolution was detected in CH_3_CN under visible light irradiation (λ > 420 nm) when triethylamine was used as the sacrificial agent (SA). The polymer was unstable under these conditions as verified by UV/visible spectroscopy. However, using ascorbic acid as the SA, hydrogen evolution (*ca*. 108 μ mol h^−1^ g^−1^) was observed under visible light irradiation (λ > 420 nm) (Suryani et al., 2019; Xie et al., 2019).

The efficiency of hydrogen generation was investigated by varying the pH of the ascorbic acid solution added to the 8 mL 1:1 (v/v) photocatalytic solution. Preliminary photocatalysis experiments were carried out using 0.1 M ascorbic acid solution that was adjusted to a pH of ~2 prior to addition to the photocatalytic solution. It is assumed under these reaction conditions that ascorbic acid exists in its fully protonated form, H_2_A (Supplementary Figure 42, pKa = 4.17) (Pellegrin and Odobel, 2017). When we adjusted the pH of the ascorbic acid solution from 2 to 5 prior to sample preparation, an increase in hydrogen evolution (0.2–108 μ mol h^−1^ g^−1^, respectively, Supplementary Table 3) was observed. While we cannot accurately determine the pH value of the final photocatalytic solution, it is assumed that the decreased degree of acidity in the later photocatalytic solution results in monoprotic ascorbate anion (HA^−^) as the dominating species. HA^−^ has previously been reported as a stronger reducing agent than the corresponding pronated form of the sacrificial agent, suggesting a plausible reason for the enhancement of hydrogen activity (Creutz, 1981; Reynal et al., 2015).

**Figure 9:**
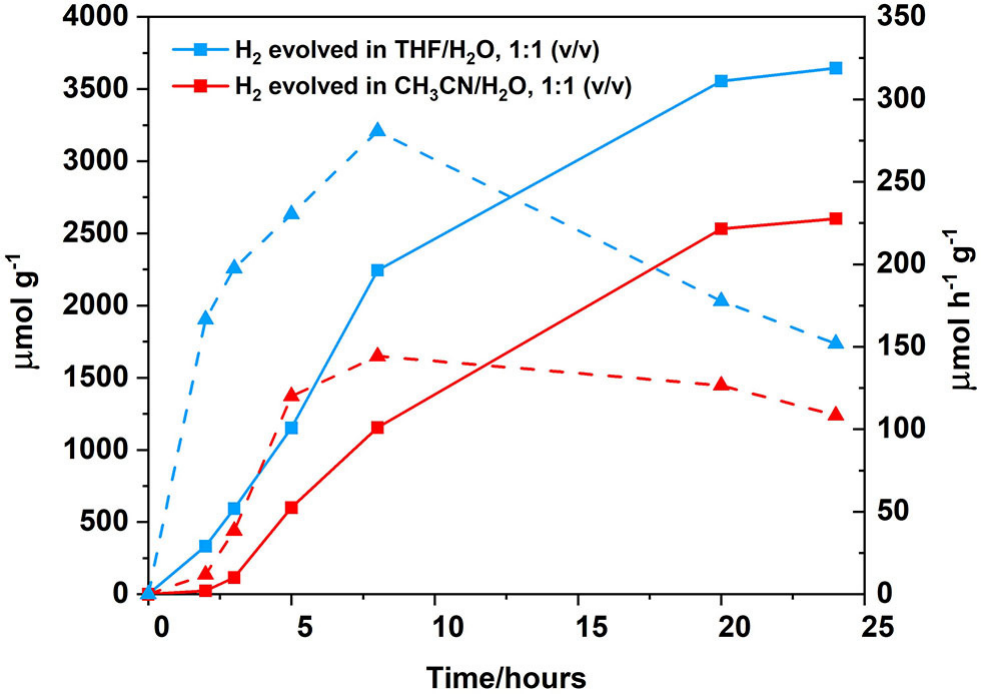
Photocatalytic results following irradiation at λ > 420 nm, cobaloxime as the catalyst (2.5 mM), ascorbic acid as the SA (0.1 M, which was adjusted to pH 5 prior to sample preparation using the appropriate amount of 2 M NaOH), polymer as PS (0.06 mg/mL). Hydrogen evolution curve displayed for different solvent ratios: THF/H_2_O, 1:1 (v/v) (blue squares, solid line) or CH_3_CN/H_2_O, 1:1 (v/v) (red squares, solid line). Hydrogen turnover frequency displayed for each solvent system in μmol h^−1^ g^−1^: THF/H_2_O, 1:1 (v/v) (blue triangles, dashed line) and CH_3_CN/H_2_O, 1:1 (v/v) (red triangles, dashed line). All samples where degassed using three freeze-pump thaw cycles prior to irradiation.

**Figure 10:**
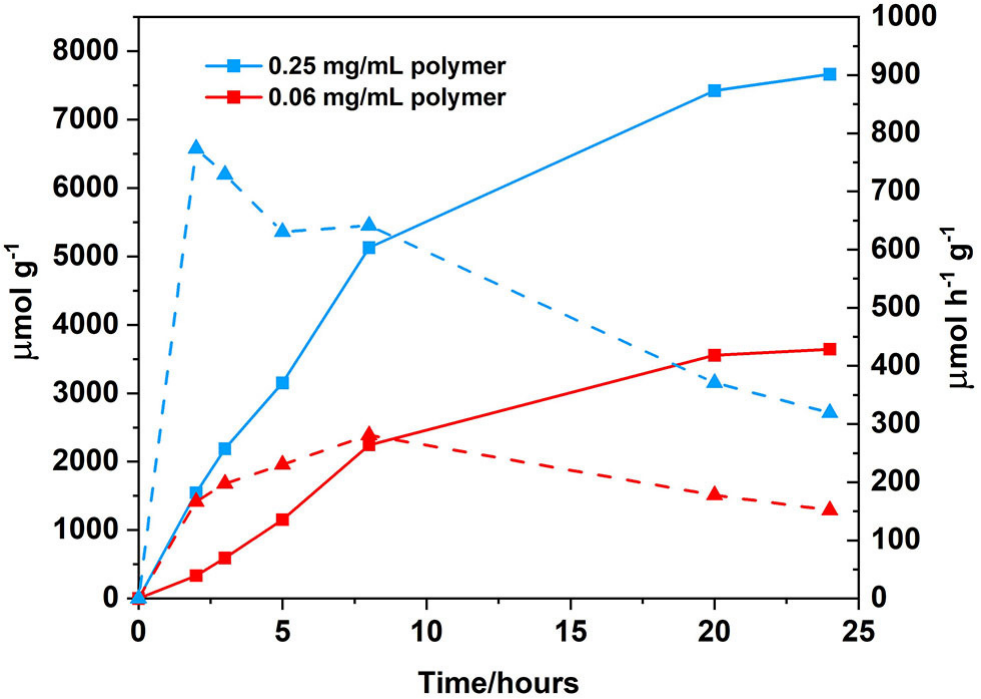
Photocatalytic results following irradiation at λ > 420 nm, cobaloxime as the catalyst (2.5 mM), ascorbic acid as the SA (0.1 M, which was adjusted to pH 5 prior to sample preparation using the appropriate amount of 2 M NaOH), solvent ratio THF/H_2_O, 1:1 (v/v) and polymer as PS (0.25 mg/mL; blue squares, solid line) or (0.06 mg/mL; red squares, solid line). Hydrogen turnover frequency displayed for each polymer concentration analysed in μmol h^−1^ g^−1^: 0.25 mg/mL (blue triangles, dashed line) and 0.06 mg/mL (red triangles, dashed line). All samples where degassed using three freeze-pump thaw cycles prior to irradiation.

Changing the solvent system from a 1:1 CH_3_CN/H_2_O (v/v) 0.1 M ascorbic acid solution to a 1:1 THF/H_2_O (v/v) 0.1 M ascorbic acid solution resulted in an increase in hydrogen generation from 108 μ mol h^−1^ g^−1^ after 24 h of irradiation to 152 μmol h^−1^ g^−1^ (Figure 9). Solvent effects are well-known to affect hydrogen generation and THF has been previously reported to be the solvent of choice for photocatalytic systems utilising BODIPY chromophores (Artero et al., 2011; Suryani et al., 2019). Furthermore, upon increasing the concentration of the polymer from 0.06 to 0.25 mg/mL (Figure 10), the amount of H_2_ evolved increased from 3,645 to 7,664 μmol g^−1^, with a corresponding increase in hydrogen turnover frequency of 152–319 μmol h^−1^ g^−1^ after 24 h.

At low concentration, the turnover frequency (TOF) increases until 8 h of irradiation, after which the activity decreases. At 24 h, the TOF is low and no further H_2_ is evolved, indicating degradation of the catalyst. Cobaloxime catalysts are known to have limited stability (Lazarides et al., 2009; Willkomm and Reisner, 2018). A summary of all photocatalytic conditions and hydrogen evolution graphs can be found in supporting information (Supplementary Figure 41 and Supplementary Table 3), and a summary of hydrogen evolution rates of organic polymers can be found in Supplementary Table 4. The majority of hydrogen evolution studies using polymeric materials require a precious metal co-catalyst. For example a polybenzothiadazole conjugated network required the addition of 3 wt% Pt to yield a hydrogen evolution rate of 116 μmol h^−1^ (Yang et al., 2016). The most notable difference between the polymer reported in this study and those reported in the literature to date, is the solubility of the polymer in a wide range of organic solvents. This offers the potential of further processing, for example post-functionalisation or further fabrication onto a photocathode surface. Post-functionalisation options include displacement of the TMS group, and addition of a carboxylic acid anchoring group to facilitate surface attachment (Ho et al., 2018).

#### Immobilisation Studies

While the mechanistic insights leading to the augmented activity of proton reduction using polymeric photosensitisers remains unclear, some limitations of polymeric species have been described, including the dispersion of the polymers in aqueous solution and hence separation of excitons, leading to enhanced charge carriers generated. The limitations associated with dispersion may be overcome if the polymer is cast as a thin film, or furthermore, immobilised onto a surface, such as NiO (Summers et al., 2015; Woods et al., 2017). This strategy may increase the stability of the polymer and also improve photon absorption, as an increase in conjugation on the surface (owed to more π-π stacking on the surface) will likely decrease the optical band gap. The importance of processability and incorporation of polymeric units onto thin films has previously been acknowledged in other work with polymers for hydrogen evolution (Woods et al., 2017).

Some preliminary studies were performed where the polymer was co-adsorbed in the first instance with 4,4′-dicarboxy-2,2′-bipyridine platinum dichloride as a catalyst for H_2_ evolution. Photoelectrocatalysis experiments were carried at pH 5 in aqueous phthalate buffer due to problems with desorption at higher or lower pH. Optimum photocurrent was detected under these conditions. The absorption spectrum of the BODIPY polymer on NiO is largely consistent with that in solution, showing a broad absorption (Supplementary Figure 45). The Pt-catalyst was difficult to co-adsorb on the NiO films due to limited solubility, but it was possible to confirm the presence of this additional layer with absorption spectroscopy and Energy-dispersive X-ray spectroscopy (EDX) analysis in conjunction with scanning electron microscopy (SEM) of the films. Representative examples of chronoamperometry measurements for the sensitised NiO films are shown in the supporting information (Supplementary Figures 48, 49). Linear sweep voltammetry allowed us to determine a safe bias potential to apply to the system without permanent reduction of species at the electrode surface. This was optimised to a value of −0.3 V vs. Ag/AgCl. It is also important to account for charging on the electrode surface at the beginning of each experiment. Control experiments confirmed that bare NiO did not produce any current due to irradiation with light, ruling out light absorbing impurities in the NiO. For the films with the BODIPY polymer and catalyst, over the timescale of the experiments (~40 min) the photocurrent of the system decreased gradually once the period of continuous illumination commenced. The chronoamperometry measurements were concurrent with gas sampling for H_2_ using the in-flow setup. While the in-flow gas sampling setup is more rigorous than headspace sampling, only trace amounts of H_2_ were detected in the experiments (possibly due to the detection limit of the system), yet bubbles were generated on the NiO surface during the experiment. Extended irradiation experiments up to 2 h 15 min did not see an increase in the amount of hydrogen detected. Chronoamperometry measurements of the polymer with the Pt-catalyst show an initial current density of ~18 μA cm^−2^, but this decreased to ~2.5 μA cm^−2^ after an initial decay (Supplementary Figure 48). There are many possible losses in the system due to dye/catalyst desorption and reaction. Surface wetting is also a consideration as bubbles of gas can become stuck on the electrode surface. Future research will necessitate identifying a more suitable co-catalyst with better loading on the NiO surface. Few easily synthesised examples of such catalysts with suitable anchoring groups exist, even fewer derived from earth abundant metals. Alternative strategies, such as surface treatments to improve wetability or doping the NiO with graphene to improve charge transport and dye loading are also attractive alternatives (Zannotti et al., 2019) but beyond the scope of the present study.

## Conclusion

In summary, the synthesis, characterisation, and photophysical properties of a TMS-BODIPY monomer and a novel TMS-BODIPY copolymer is reported. Modification of the BODIPY monomer facilitating close proximity of multiple chromophores in a polymeric backbone led to significant changes in the photophysical properties. Time-resolved techniques, such as TA and TRIR identified both singlet and triplet excited states and the related kinetics of these species. The photophysics of this polymer are sensitive to the surrounding solvent, facilitating transition to the triplet excited state via a charge transfer state. Photocatalytic hydrogen evolution studies in solution demonstrated that hydrogen evolution occurs using visible light in solution. This work shows that BODIPY copolymers can act as effective photosensitisers for hydrogen evolution, and how they can be used in artificial photosynthetic applications.

## Data Availability Statement

The raw data supporting the conclusions of this article will be made available by the authors, without undue reservation.

## Author Contributions

MP and AC: synthesis, time resolved studies, and photocatalysis. LO'R, MP, and GG: time resolved studies. KH: synthesis. CL: time resolved studies/Glotoran. EG and JK: photocatalysis. AH and RM: polymer MW. All authors contributed to the article and approved the submitted version.

## Conflict of Interest

The authors declare that the research was conducted in the absence of any commercial or financial relationships that could be construed as a potential conflict of interest.
