# Supplementary material for: A Time-Resolved Spectroscopic Investigation of a Novel BODIPY Copolymer and Its Potential Use as a Photosensitiser for Hydrogen Evolution
Source: Front Chem. 2020 Oct 19;8:584060. doi: 10.3389/fchem.2020.584060 (PMC7604388; doi:10.3389/fchem.2020.584060)
Supplement: Supplementary file 1 [file Data_Sheet_1.docx]

Supplementary Material

**Table of Contents**

[1 Synthetic procedure 2](#_Toc48739654)

[2 NMR spectra 5](#_Toc48739655)

[3 Mass Spectroscopy 6](#_Toc48739656)

[4 Size Exclusion chromatography (SEC) 7](#_Toc48739657)

[5 FTIR spectra 8](#_Toc48739658)

[6 Absorption spectra 9](#_Toc48739659)

[7 Excitation spectra 10](#_Toc48739660)

[8 Emission spectra 12](#_Toc48739661)

[9 Emission map experiments 14](#_Toc48739662)

[10 Singlet oxygen measurements 17](#_Toc48739663)

[11 Lifetime measurements 18](#_Toc48739664)

[12 Optical gap determination 21](#_Toc48739665)

[13 Steady state UV-visible absorption and emission spectra 22](#_Toc48739666)

[14 Transient absorption spectra (ps-timescale) 23](#_Toc48739667)

[15 Transient absorption spectroscopy (ns-timescale) 25](#_Toc48739668)

[16 Time Resolved Infrared Spectroscopy (ps-timescale) 26](#_Toc48739669)

[17 Summary of photophysical properties 29](#_Toc48739670)

[18 Photocatalytic hydrogen evolution experiments in solution 30](#_Toc48739671)

[19 Photoelectrochemical Hydrogen Evolution using NiO Photocathodes 32](#_Toc48739672)

[20 Literature review of organic-based polymers for hydrogen evolution 36](#_Toc48739673)

[21 Compounds in solution 38](#_Toc48739674)

[22 References 39](#_Toc48739675)

# Synthetic procedure


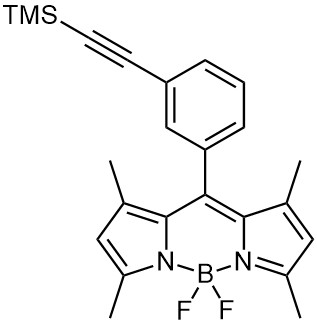


**3-TMS BODIPY monomer.** The synthesis of BODIPY monomers were carried out as previously reported with some minor modifications.^1^ To an oven dried flask fitted with magnetic stir-bar, 25 mL CH_2_Cl_2_ was added and allowed to purge with N_2_ for 15 min. To the degassed solvent, 3-[(trimethylsilyl)ethynyl] benzaldehyde (2.5 mmol) and 2,4-dimethylpyrrole (0.51 mL, 5.0 mmol) were added. A few drops of TFA was added and the solution was stirred overnight at room temperature, in the absence of light. DDQ (2.48 mmol was added and the solution was allowed to stir for a further 4 h. After this time, boron trifluoride diethyl etherate (18.4 mmol) was added, following in quick succession by TEA (41 mmol). The reaction was allowed to stir overnight at room temperature under the flow of nitrogen. After this time, the reaction was stopped, and the organic layer was collected after washing three times with saturated sodium bicarbonate solution. The organic layer was collected and dried over magnesium sulphate to yield a dark purple crude product. Purification was carried out using column chromatography with on silica (hexane: ethyl acetate 70:30) to yield a red solid. ^1^H NMR (600 MHz, CDCl_3_): δ 7.56 (dt, J = 7.8 Hz, 1H), 7.45 – 7.42 (m, 2H), 7.24 (dt, J = 7.6 Hz, 1H), 5.98 (s, 2H), 2.55 (s, 6H), 1.41 (s, 6H), 0.24 (s, 9H). ^13^C NMR (600 MHz, CDCl_3_): δ 155.9, 143.2, 140.5, 135.3, 132.6, 131.6, 131.5, 129.3, 128.2, 124.6, 121.5, 104.0, 96.0, 14.9, 14.8, 0.1. HRMS (ESI) calculated for C_24_H_27_BF_2_N_2_Si (M+H), 421.2077, obtained 421.2074.


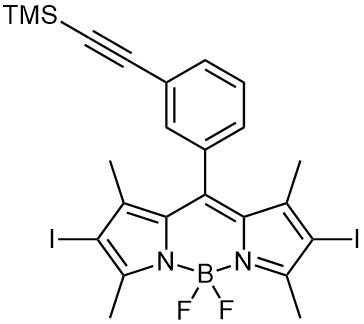


**3-TMS diiodo BODIPY**: This procedure was carried out as per previous reported with some minor modifications.^1^ 3-TMS BODIPY monomer (0.26 mmol) and iodine (76 mg, 0.30 mmol) were added to an oven-dried round bottom flask equipped with stir-bar, dissolved in 30 mL of EtOH and purged for 30 min with nitrogen. Iodic acid (93 mg, 0.53 mmol) was dissolved in 1.5 mL deionised water and also allowed to purge with nitrogen for 30 min. After this time, both solutions were combined and allowed to reflux at 60 °C for 20 min. When the complete conversion of the monomer was observed by monitoring the reaction by TLC, the solvent was removed using distillation, followed by dissolving the crude product in CH_2_Cl_2_ and washing three times with saturated sodium thiosulphate solution. The organic layer was collected, and the product was dried using rotary evaporation to yield pure product. ^1^H NMR (600 MHz, CDCl_3_): δ 7.61 (d, J = 7.7 Hz, 1H), 7.47 (t, 1H), 7.38 (s, 1H), 7.20 (d, J = 7.6 Hz, 1H), 2.64 (s, 6H), 1.43 (s, 6H), 0.25 (s, 9H). ^13^C NMR (600 MHz, CDCl_3_): δ 157.3, 145.5, 140.2, 135.1, 133.1, 131.4, 131.3, 129.6, 127.9, 125.0, 103.6, 96.6, 86.0, 17.5, 16.2, 0.1. HRMS (ESI) calculated for C_24_H_25_BF_2_I_2_N_2_Si, 671.9937, obtained 671.9922.


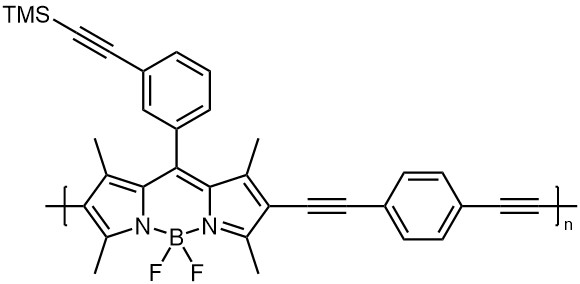


**3-TMS BODIPY polymer:** To an oven-dried Schlenk equipped with stir-bar, anhydrous tetrahydrofuran (THF) (15 mL) and anhydrous diisopropylamine (DiiPA) (15 mL) were added. All glass taps were greased and the Schlenk was degassed using three freeze-pump-thaw cycles. Addition of the solids to the Schlenk was then carried out – BODIPY iodo monomer, B2, (0.15 mmol), 1,4-diethynylbenzene (19 mg, 0.15 mmol), bis(triphenylphosphine) palladium(II) dichloride (16 mol%), copper iodide ( 8 mol%), triphenylphosphine (8 mol%) were added, and the freeze-pump-thaw cycle was repeated to ensure the reaction would occur in the absence of air. The solution was allowed to reflux for 48 hr at 75-85 °C, until complete consumption of the IODO BODIPY was monitored by TLC. After this time, the solvents were removed by distillation. The crude polymer was dissolved in CH_2_Cl_2_ and washed three times with saturated sodium bicarbonate solution. The organic layer was dried over MgSO_4_. Purification was carried out by washing in EtOH and collection of the precipitate to yield a pink/purple solid. This process was repeated to yield a pure polymer free of residual catalytic system. 3-TMS polymer was obtained as a dark purple solid (94 mg). ^1^H NMR (600 MHz, CDCl_3_): δ 7.73-7.27 (br, Ar-H), 2.64 (br, CH_3_ at 3, 5 position of BODIPY), 1.43 (br, CH_3_ at 1, 7 position of BODIPY), 0.26 (br, CH_3_ x 3 in TMS group at *meso* position of BODIPY).SEC results: Peak 1: M_n_ = 12260, M_w_ = 13990, PDI = 1.141; Peak 2: M_n_ = 4801, M_w_ = 4952, PDI = 1.031.


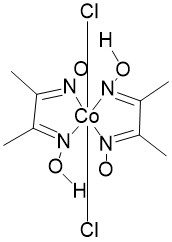


**Synthesis of dichlorobis(dimethylglyoximato) cobalt (IV) cobaloxime [Co(dmg)_2_Cl_2_]:** 1.1g (4.62 mmol) of cobalt (II) chloride hexahydrate was added to an oven-dried 50 mL round bottom flask followed by 15 mL acetone. 1.07 g (9.24 mmol) of dimethylglyoxime was dissolved in an additional 15 mL acetone and added to the reaction flask. Sonication was required to fully dissolve the dimethylglyoxime in acetone prior to addition to the flask. The reaction was allowed to stir for 60 min at room temperature under the flow of air to allow oxidation to occur. A dark green precipitate was observed in the reaction flask after this time. The flask was cooled over ice and the green solid was collected via vacuum filtration, washing 10 mL x 2 with ice-cold acetone. A green solid was obtained, 1.03 g, 2.85 mmol, 71%. ^1^H NMR (600 MHz, d^6^-DMSO) *δ* 2.33 (s, 12 H).


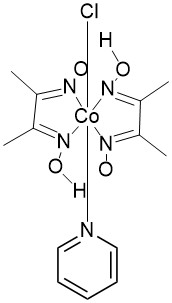


**Synthesis of chloro(pyridine)bis(dimethylglyoximato) cobalt (III) cobaloxime [Co(dmg)_2_pyCl]:** Synthesis of [Co(dmg)_2_pyCl] was carried out as per previously reported with some minor modifications.^2^ 0.3 g of [Co(dmg)_2_Cl_2_] previously synthesised, was suspended in 15 mL MeOH (poor solubility) in a 100 mL round-bottomed flask. 0.07 mL (0.833 mmol) pyridine was added in quick succession and the reaction was allowed to stir at room temperature for 30 min. A brown precipitate was observed in the reaction flask after this time. 20 mL deionised water was added to the flask, before cooling on ice for 20 min. The precipitate was collected via vacuum filtration, washing with 3 x 15 mL solution of 2:1, H_2_O: MeOH, followed by 2 x 10 mL of diethyl ether. A light brown solid results, 0.214 g, 0.530 mmol, 64%. ^1^H NMR (400 MHz, CDCl_3_) *δ* 8.24 ppm (d, 2H), 7.68 (m, 1H), 7.15 (d, 2H), 2.40 (s, 12H).

# NMR spectra


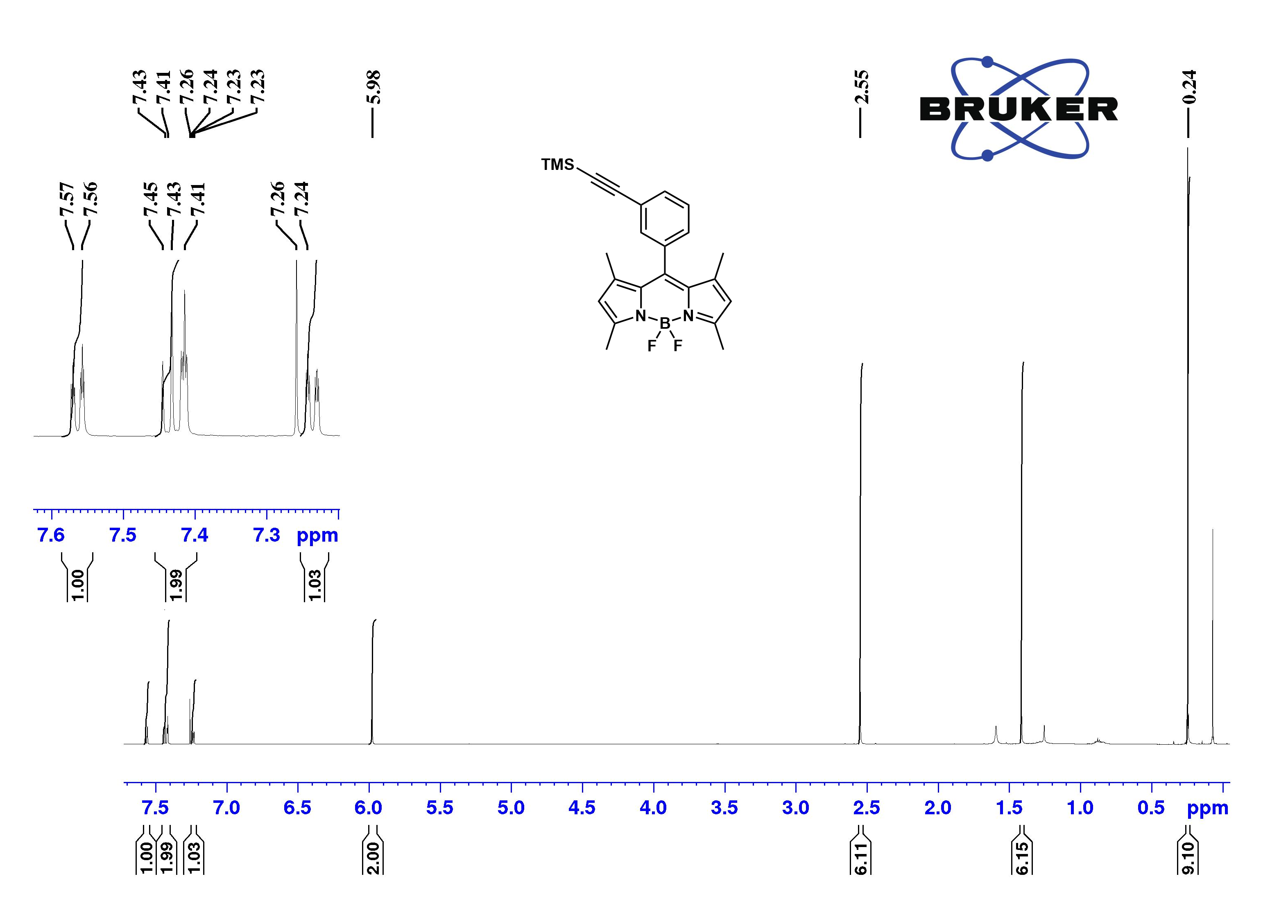


Figure S1. ^1^H NMR spectra of monomer. CDCl_3_ as calibration standard, δ = 7.26 ppm.


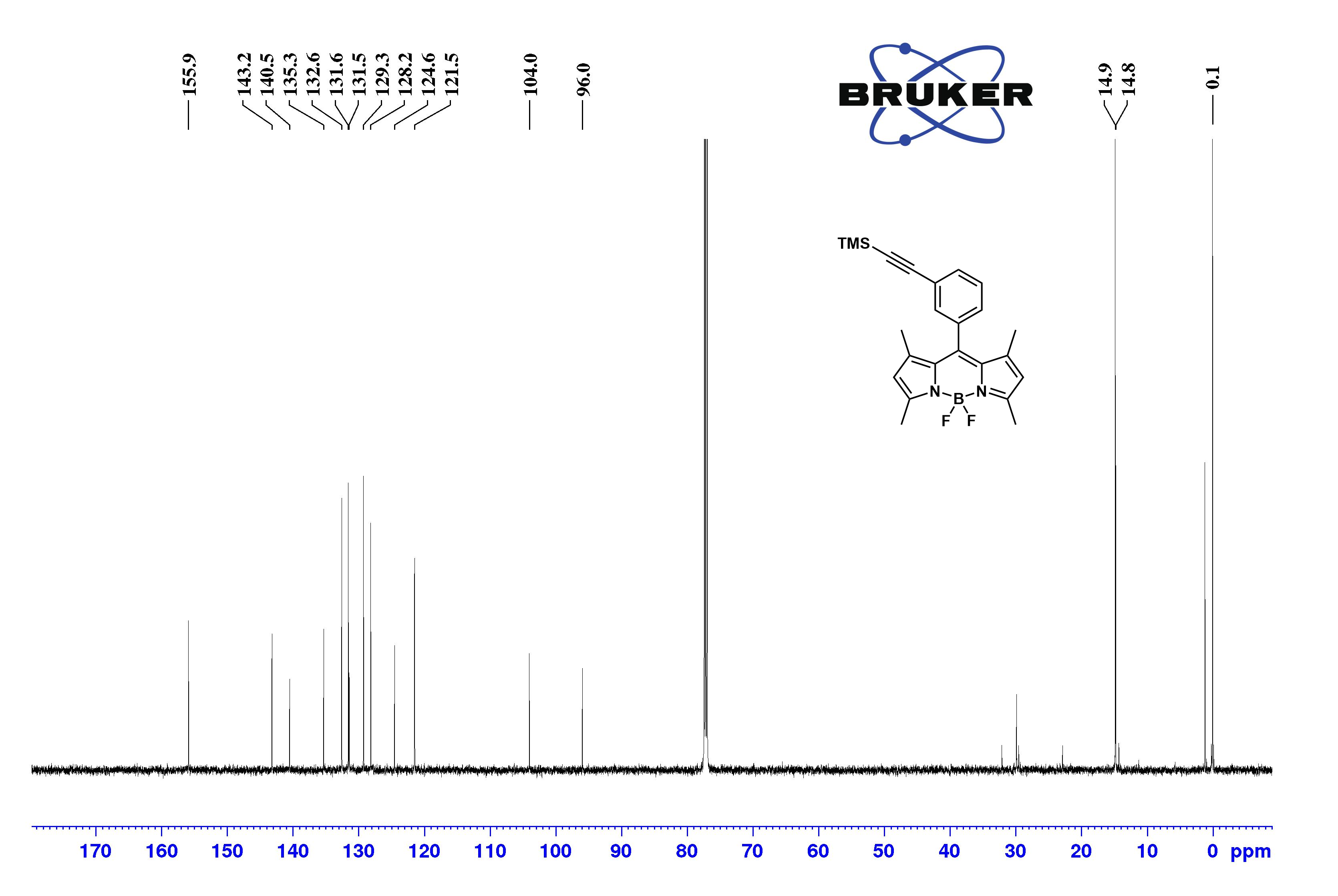


Figure S2. ^13^C NMR spectra of monomer. CDCl_3_ as calibration standard, δ = 77.16 ppm.


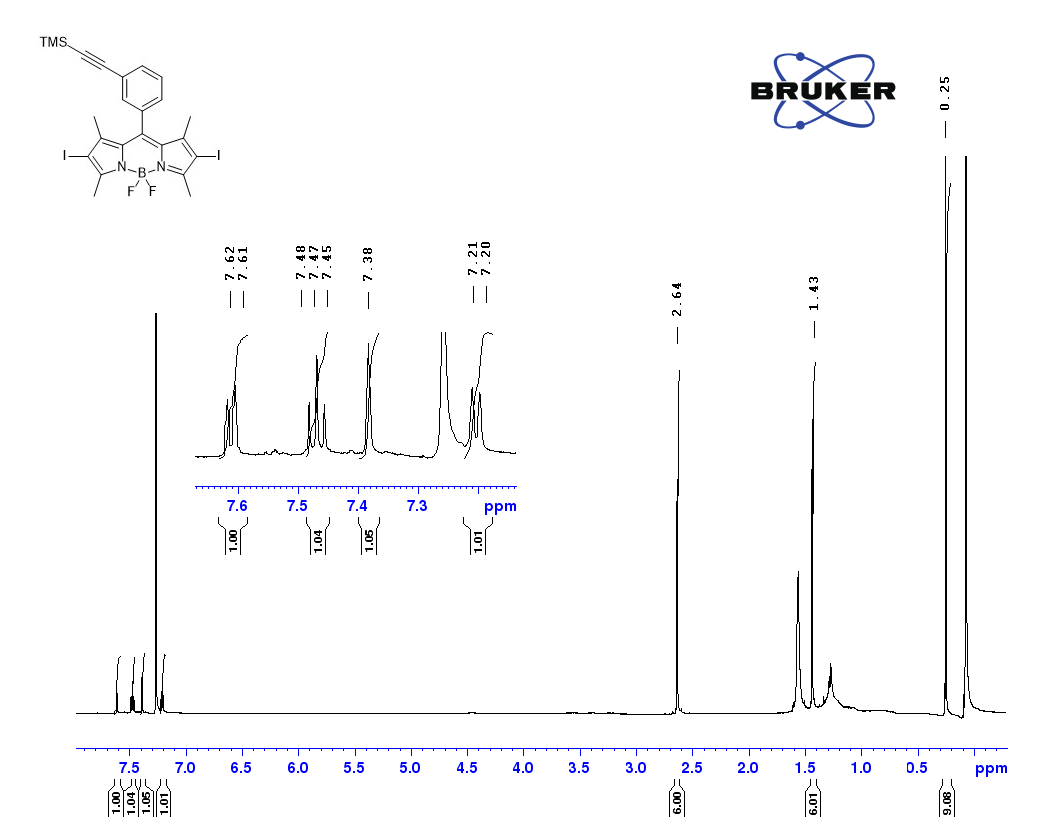


Figure S3. ^1^H NMR spectra of diiodo monomer. CDCl_3_ as calibration standard, δ = 7.26 ppm.


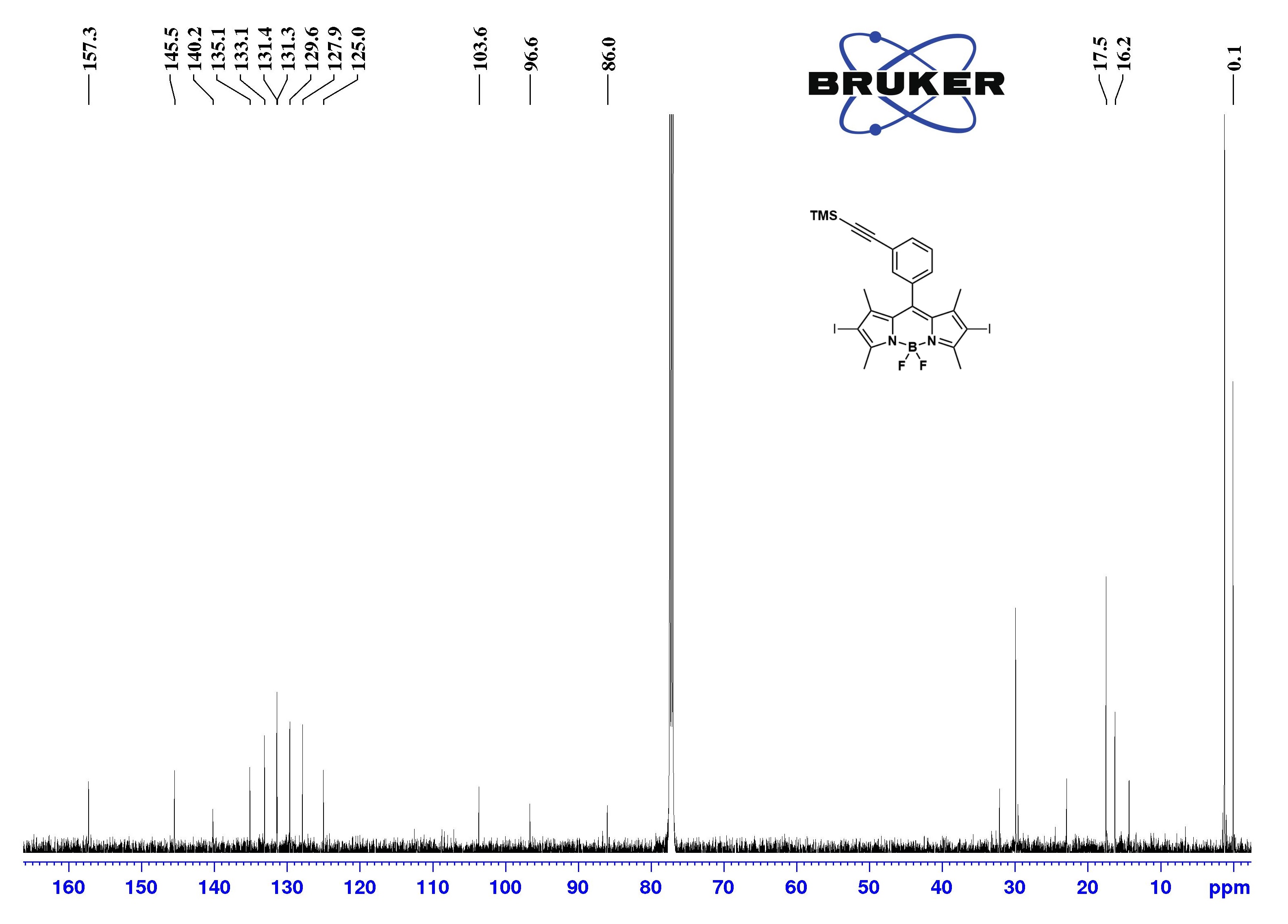


Figure S4. ^13^C NMR spectra of diiodo monomer. CDCl_3_ as calibration standard, δ = 17.16 ppm.


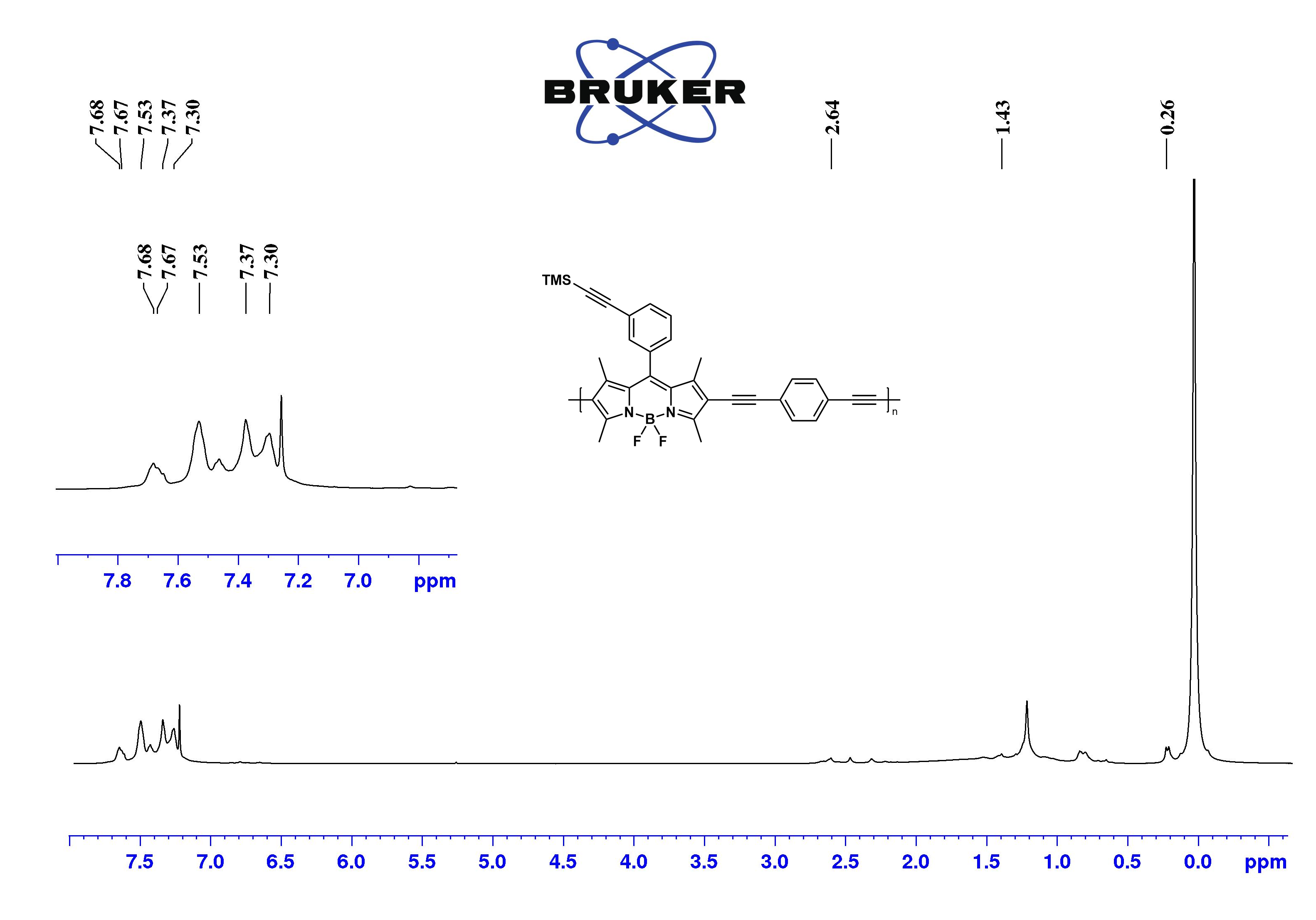


Figure S5. ^1^H NMR spectra of polymer. CDCl_3_ as calibration standard, δ = 7.26 ppm.

# Mass Spectroscopy


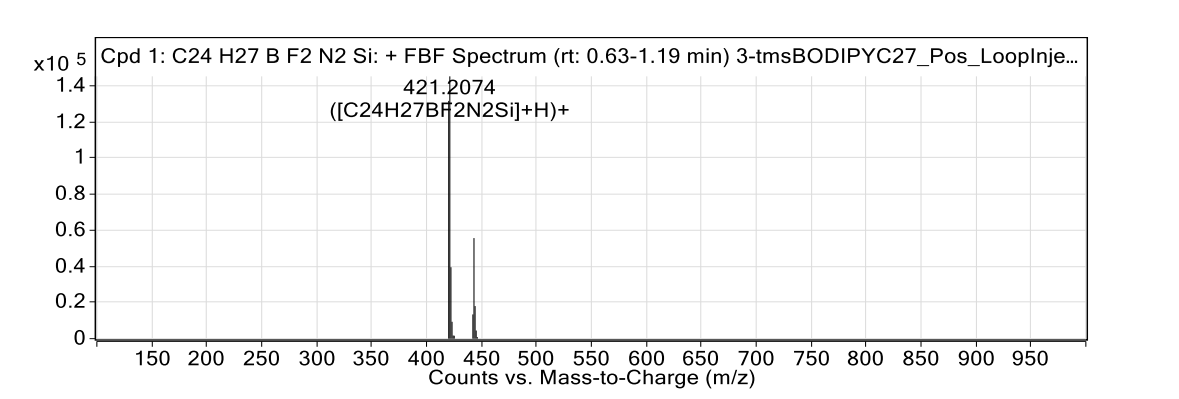


Figure S6. MS spectra [M+H] for 3-TMS BODIPY monomer


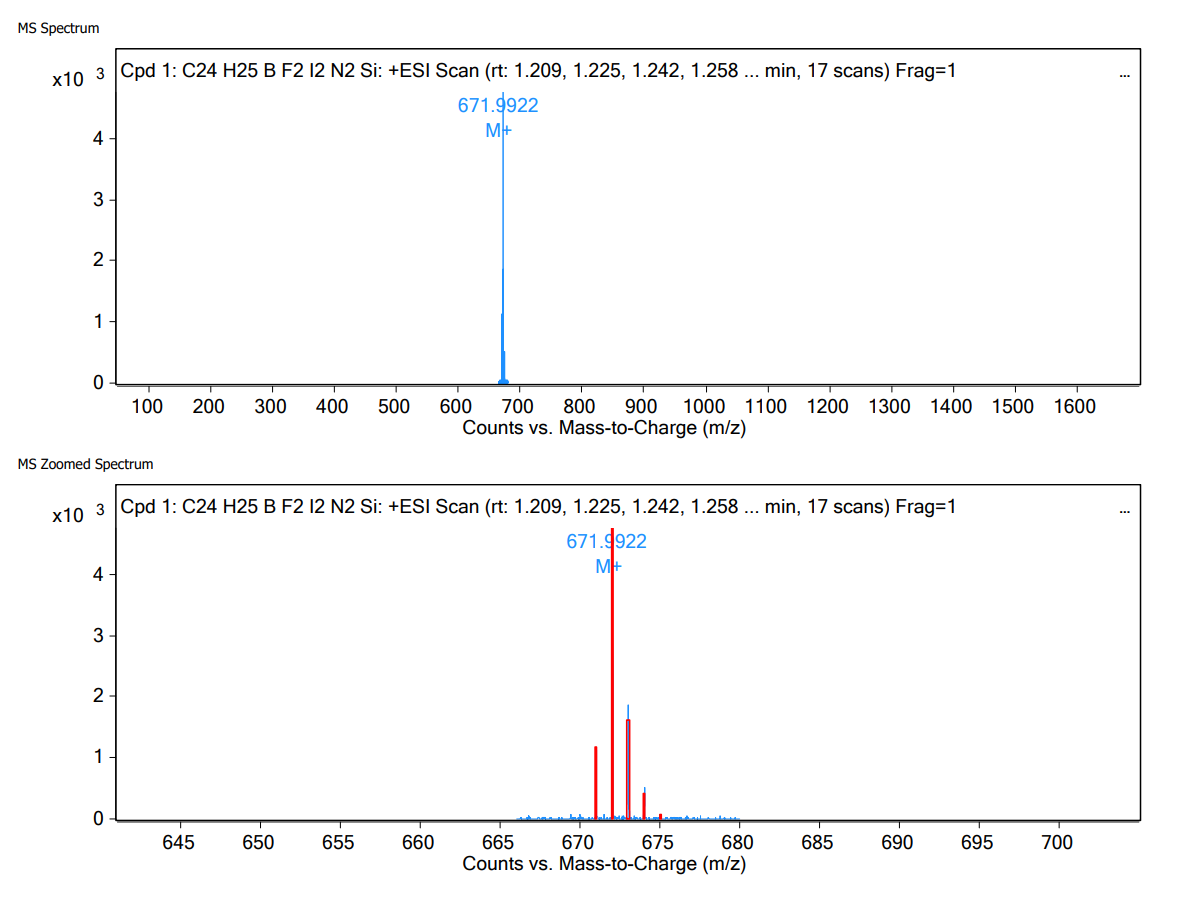


Figure S7. MS spectra for [M^+^] for 3-TMS diiodo BODIPY monomer

# Size Exclusion chromatography (SEC)


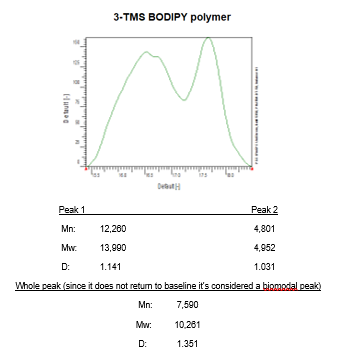


Figure S8. SEC results obtained for polymer showing the molecular weight distribution of the polymer

# FTIR spectra


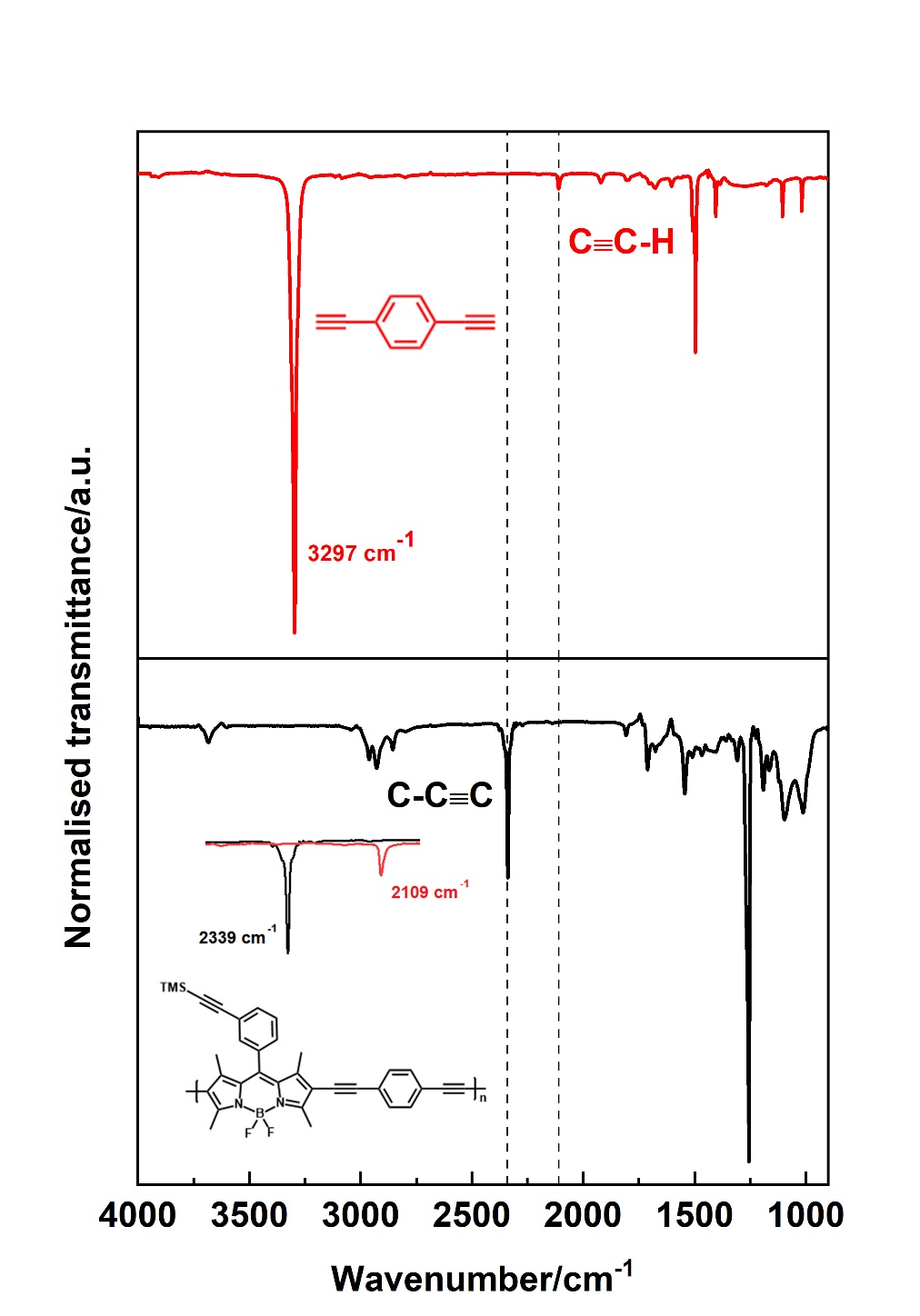


Figure S9. FTIR spectra of 1,4-diethynylbenzene (red spectra, top) and polymer (black spectra, bottom), recorded in a solution cell in spectrophotometric grade dichloromethane. Structures inserted below each IR spectra. Inset showing details between 2400 – 2000 cm^-1^.

# Absorption spectra


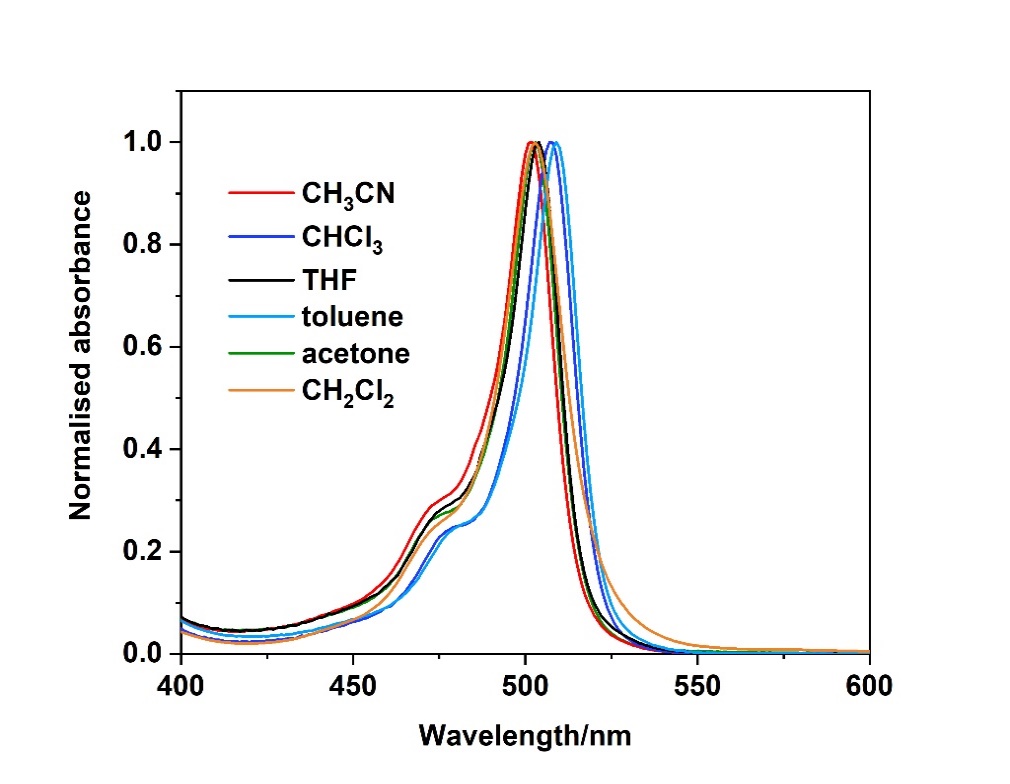


Figure S10. Normalised absorption spectra of monomer in acetonitrile (red), chloroform (dark blue), THF (black), toluene (light blue) and acetone (green), dichloromethane (orange). Recorded at room temperature.


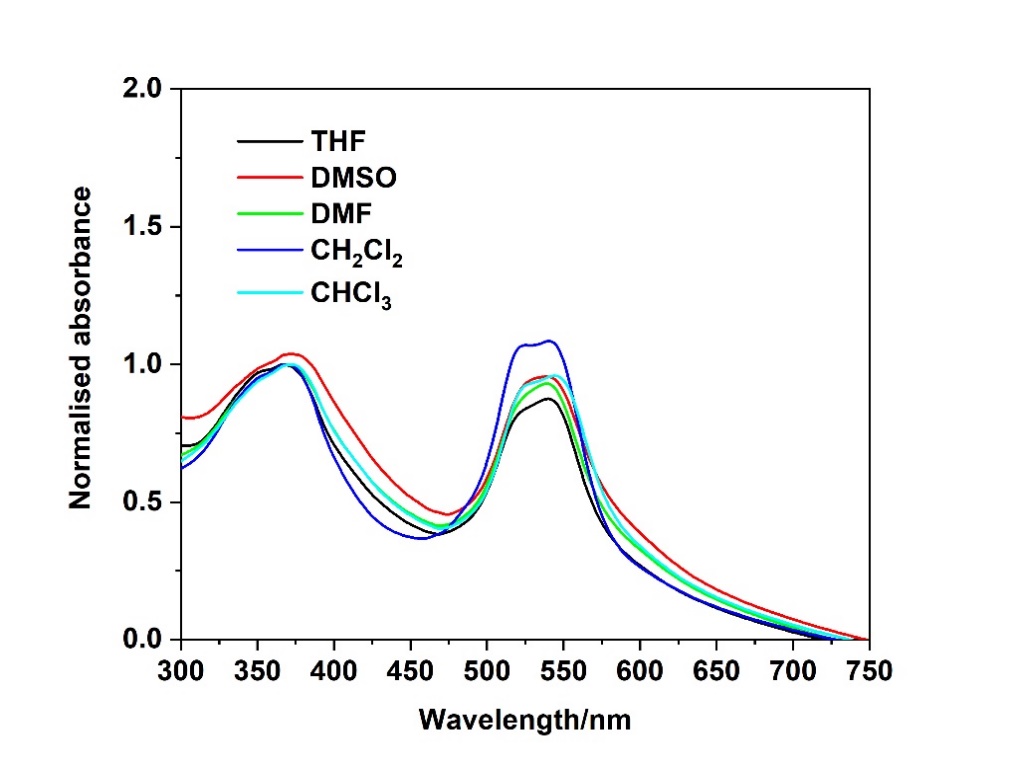


Figure S11. Normalised absorption spectra of polymer in THF (black), DMSO (red), DMF (green), dichloromethane (dark blue), chloroform (light blue). Recorded at room temperature.

# Excitation spectra


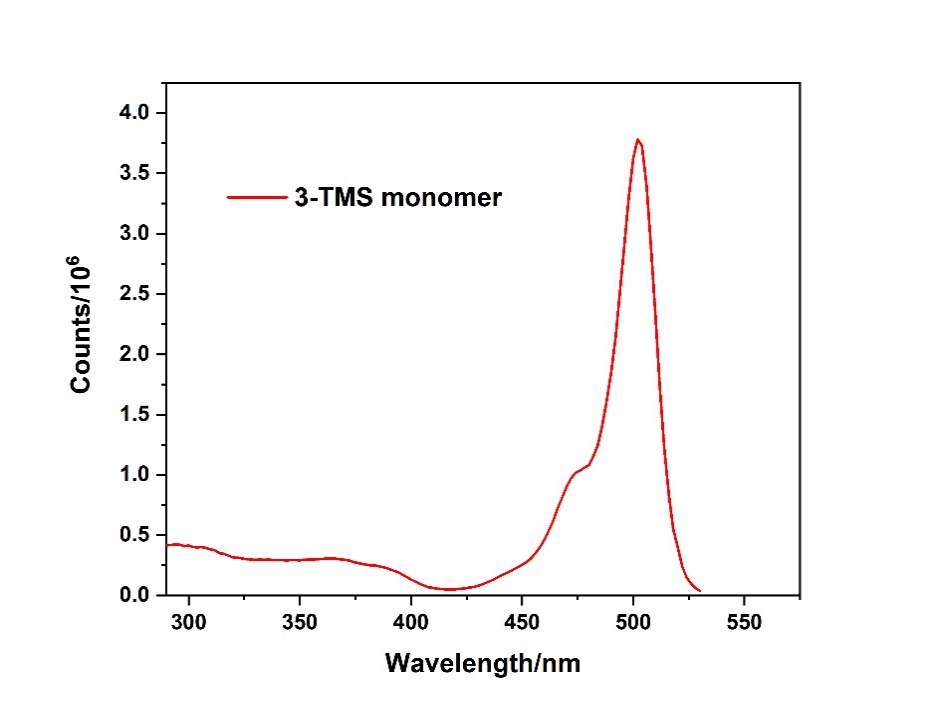


Figure S12. Excitation spectra of monomer in CH_2_Cl_2_. Recorded at the emission maxima.


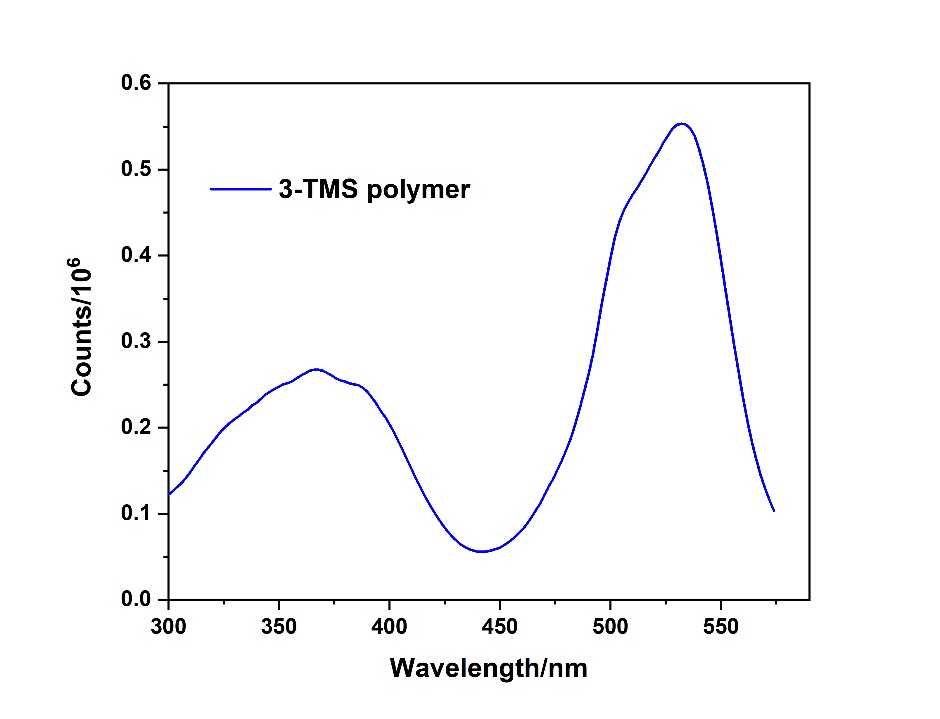


Figure S13. Excitation spectra of polymer in CH_2_Cl_2_. Recorded at the emission maxima.


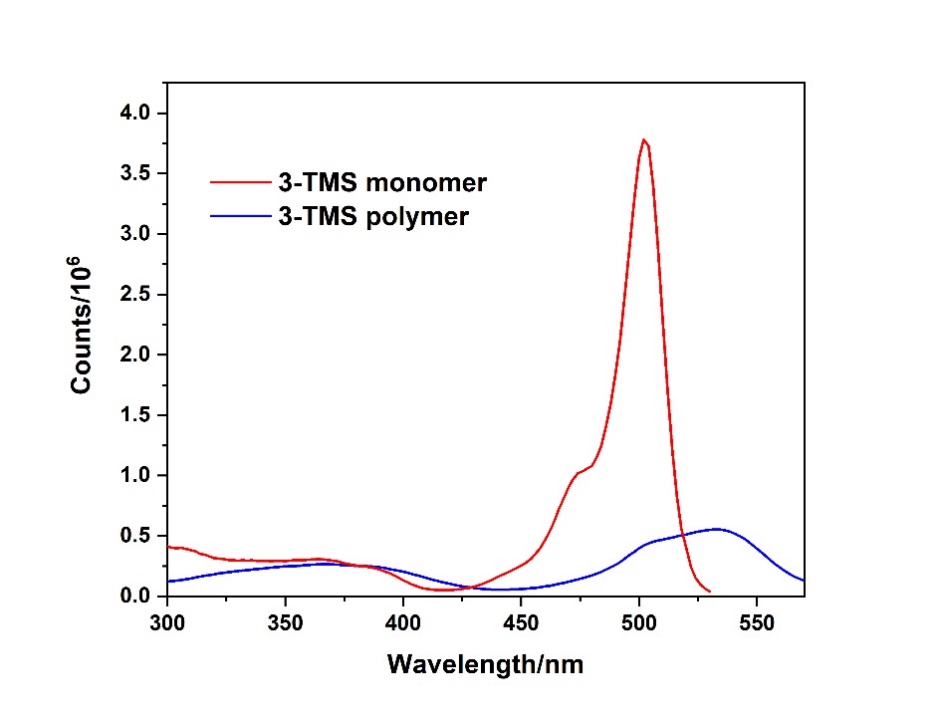


Figure S14. Excitation spectra of monomer (red) and polymer (blue) in CH_2_Cl_2_. Recorded at each emission maxima respectively.

# Emission spectra


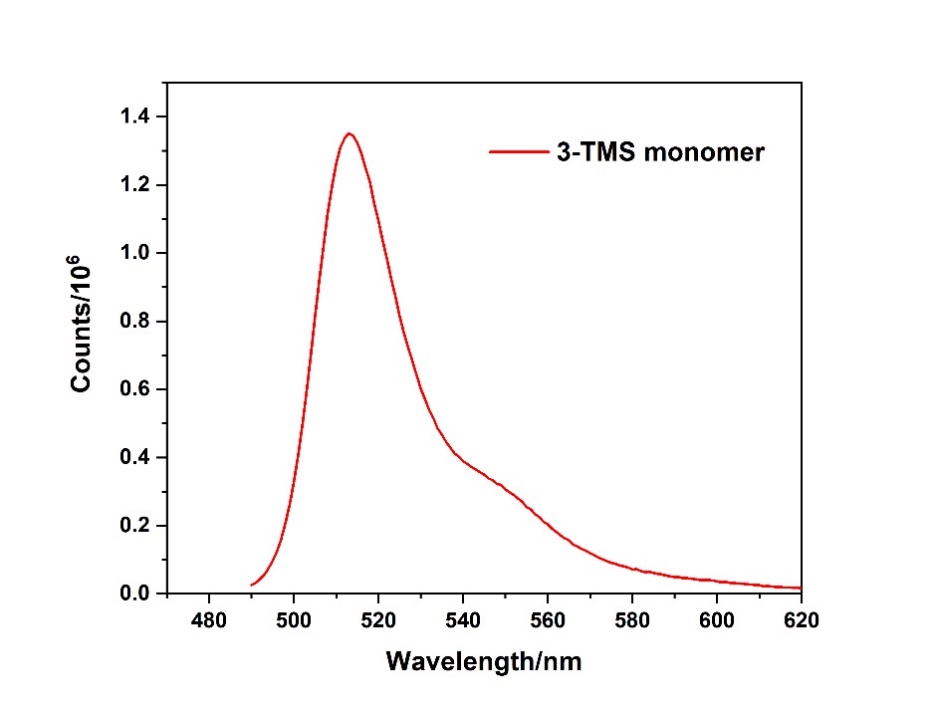


Figure S15. Emission spectra monomer in CH_2_Cl_2_. λ_exc_ corresponding to lowest energy absorption band.


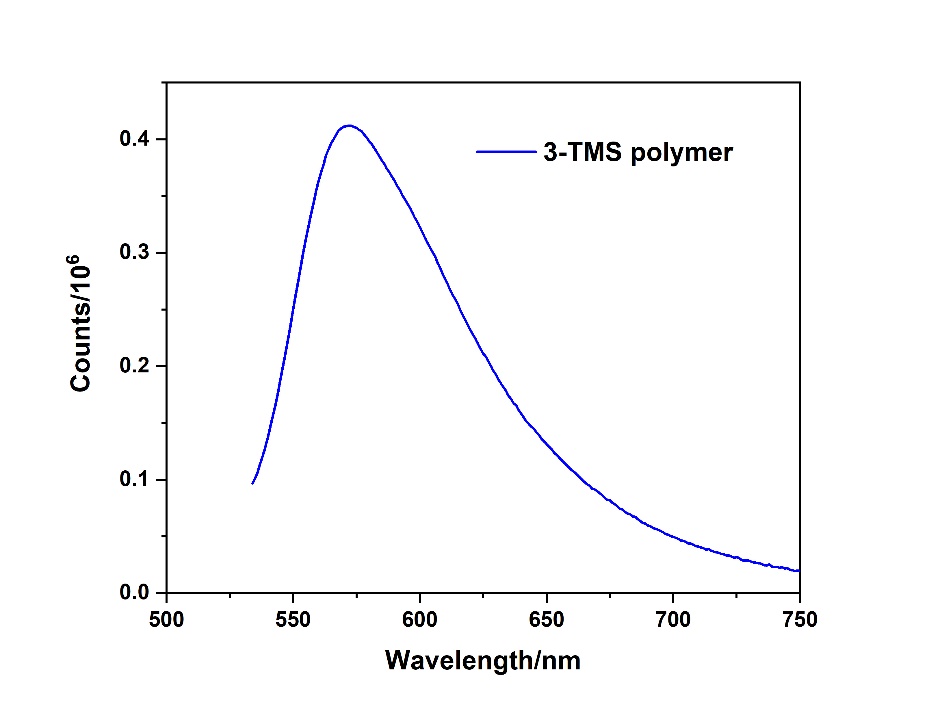


Figure S16. Emission spectra polymer in CH_2_Cl_2_. λ_exc_ corresponding to lowest energy absorption band.


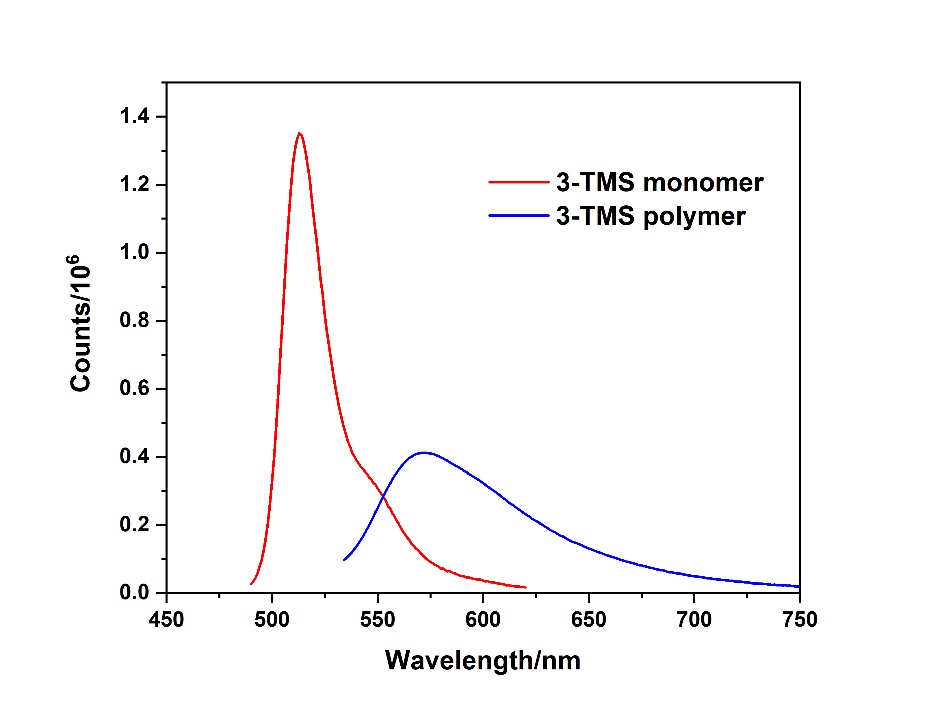


Figure S17. Emission spectra monomer (red) and 3-TMS polymer (blue) in CH_2_Cl_2_. λ_exc_ corresponding to lowest energy absorption band.

# Emission map experiments


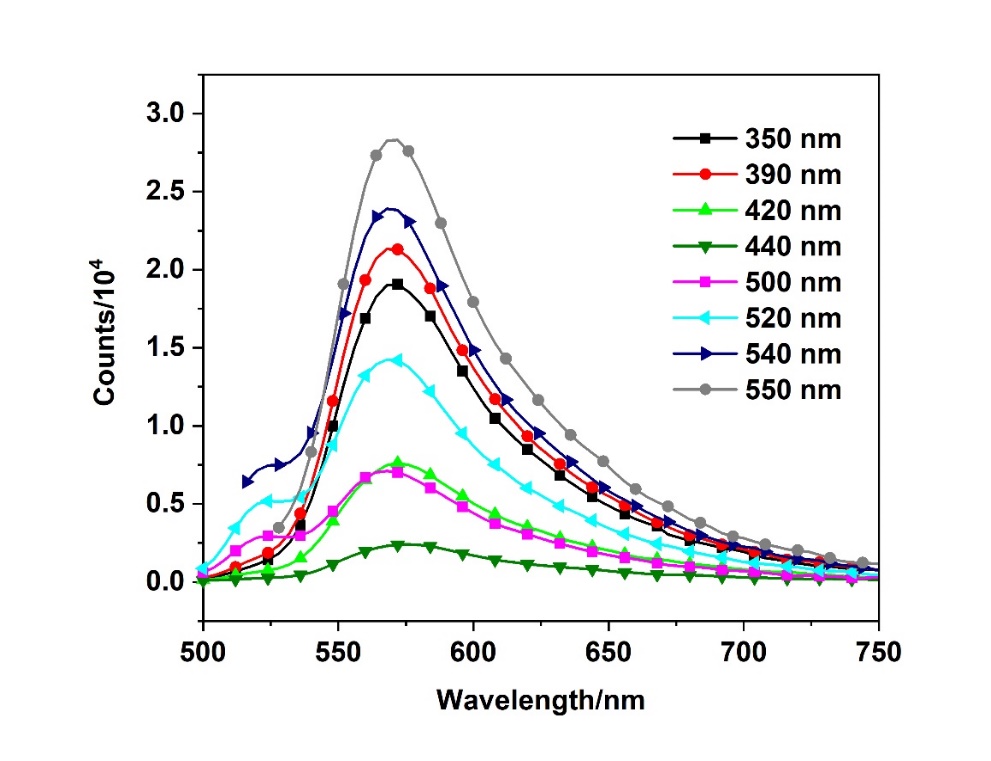


Figure S18. Emission map of polymer in dichloromethane, recorded at room temperature. Excitation wavelengths shown in inset: 350 nm (black squares), 390 nm (red circles), 420 nm (green triangles), 440 nm (olive triangles), 500 nm (magenta squares), 520 nm (cyan triangles), 540 nm (navy triangles), 550 nm (grey circles).


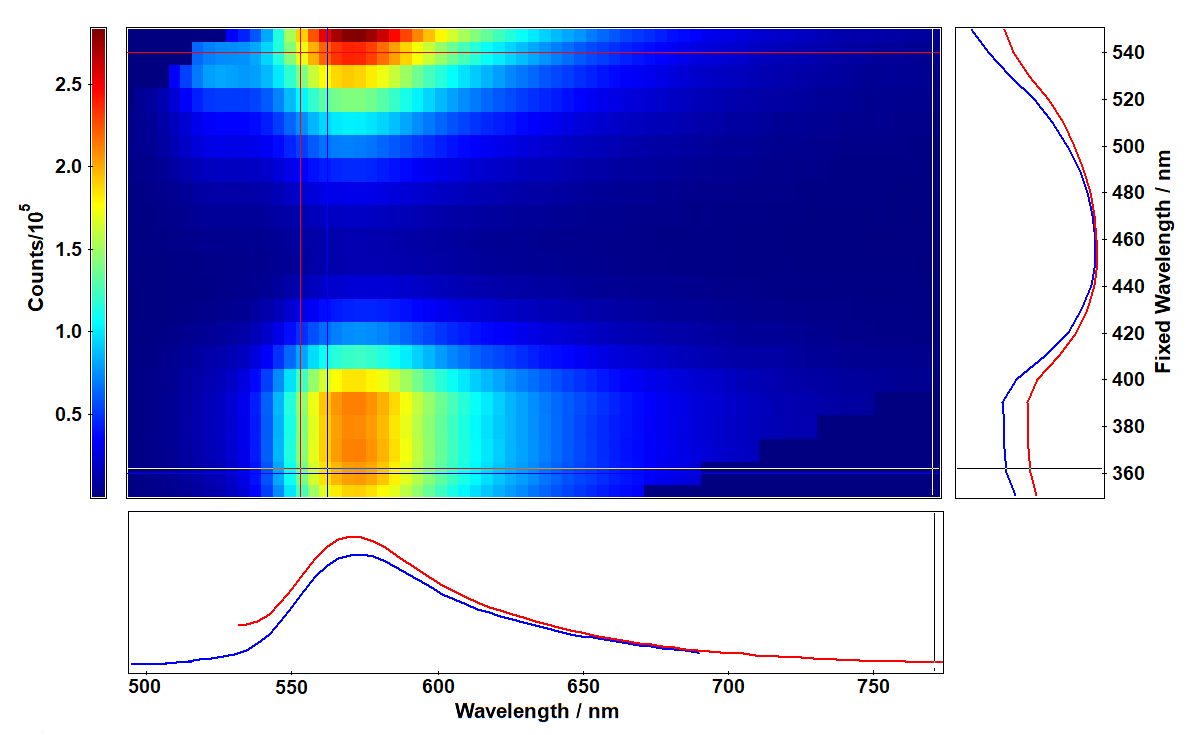


Figure S19. Excitation Emission contour map of polymer in dichloromethane, recorded at room temperature. Emission spectra shown at the bottom using λ_exc_ = 360 nm (blue) and λ_exc_ = 540 nm (red).


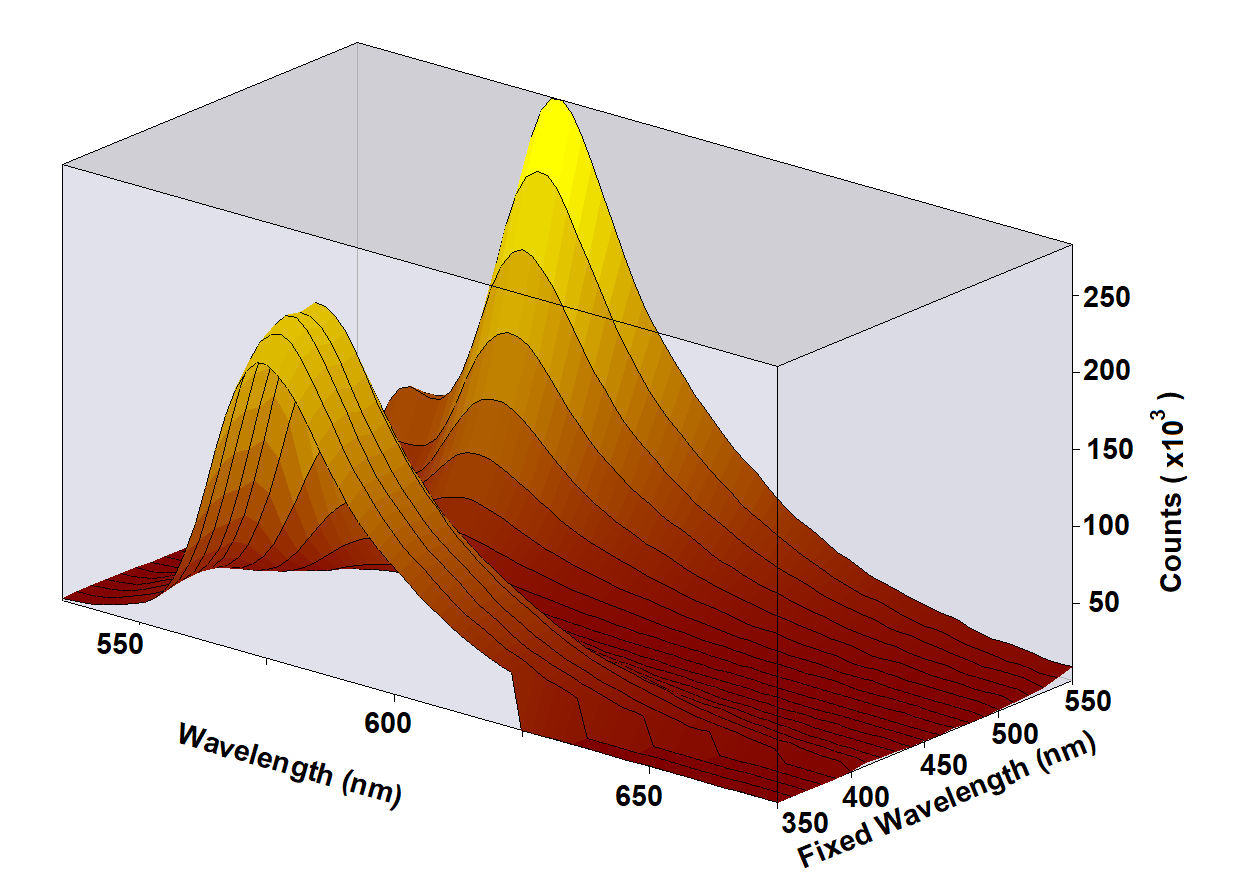


Figure S20. 3-D Emission map of polymer in dichloromethane, recorded at room temperature. Fixed wavelength axis shows varying excitation wavelengths used.


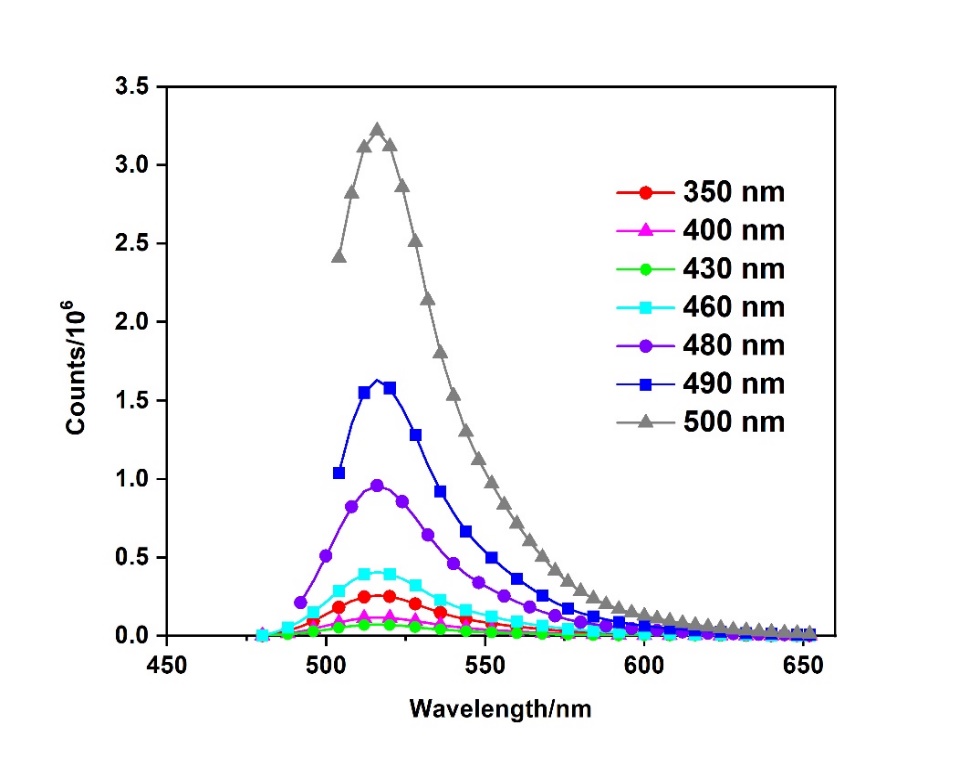


Figure S21. Emission map of monomer in dichloromethane, recorded at room temperature. Excitation wavelengths shown in inset: 350 nm (red circles), 400 nm (magenta triangles), 430 nm (green circles), 460 nm (cyan squares), 480 nm (violet circles), 490 nm (blue squares), 500 nm (grey triangles).


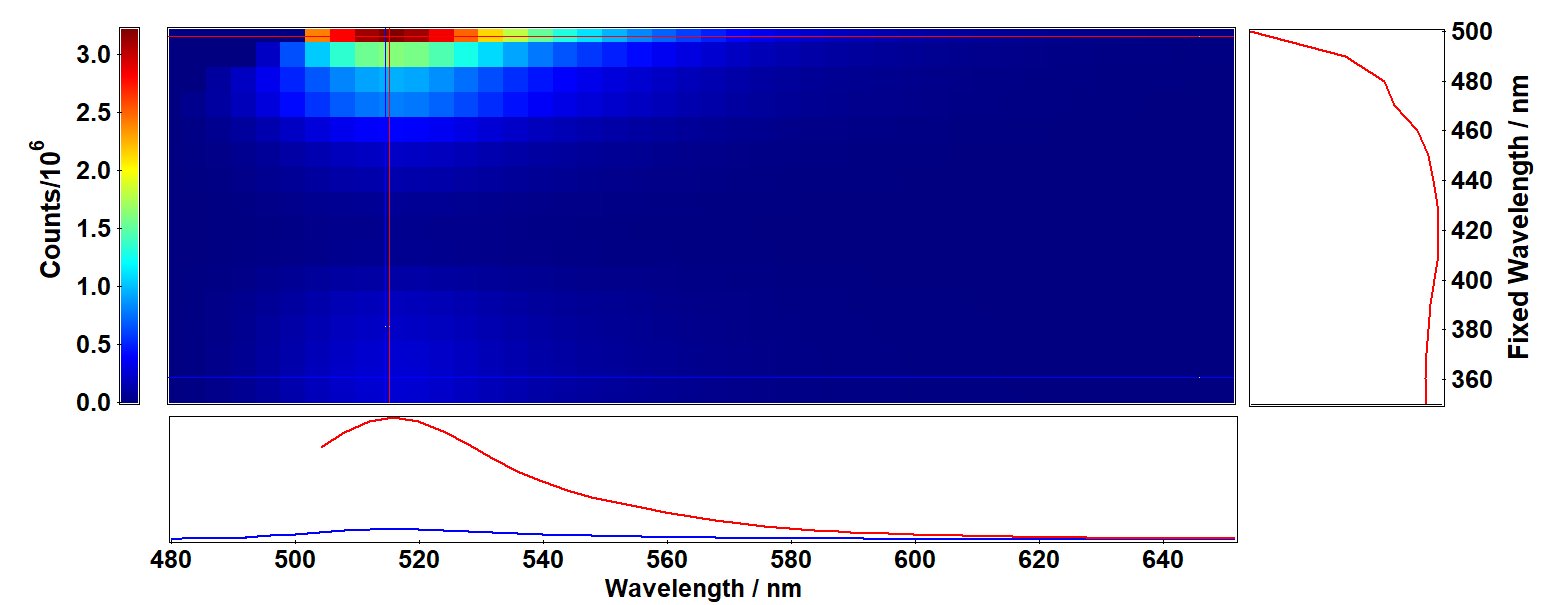


Figure S22. Excitation Emission contour map of monomer in dichloromethane, recorded at room temperature. Emission spectra shown at the bottom using λ_exc_ = 360 nm (blue) and λ_exc_ = 500 nm (red).


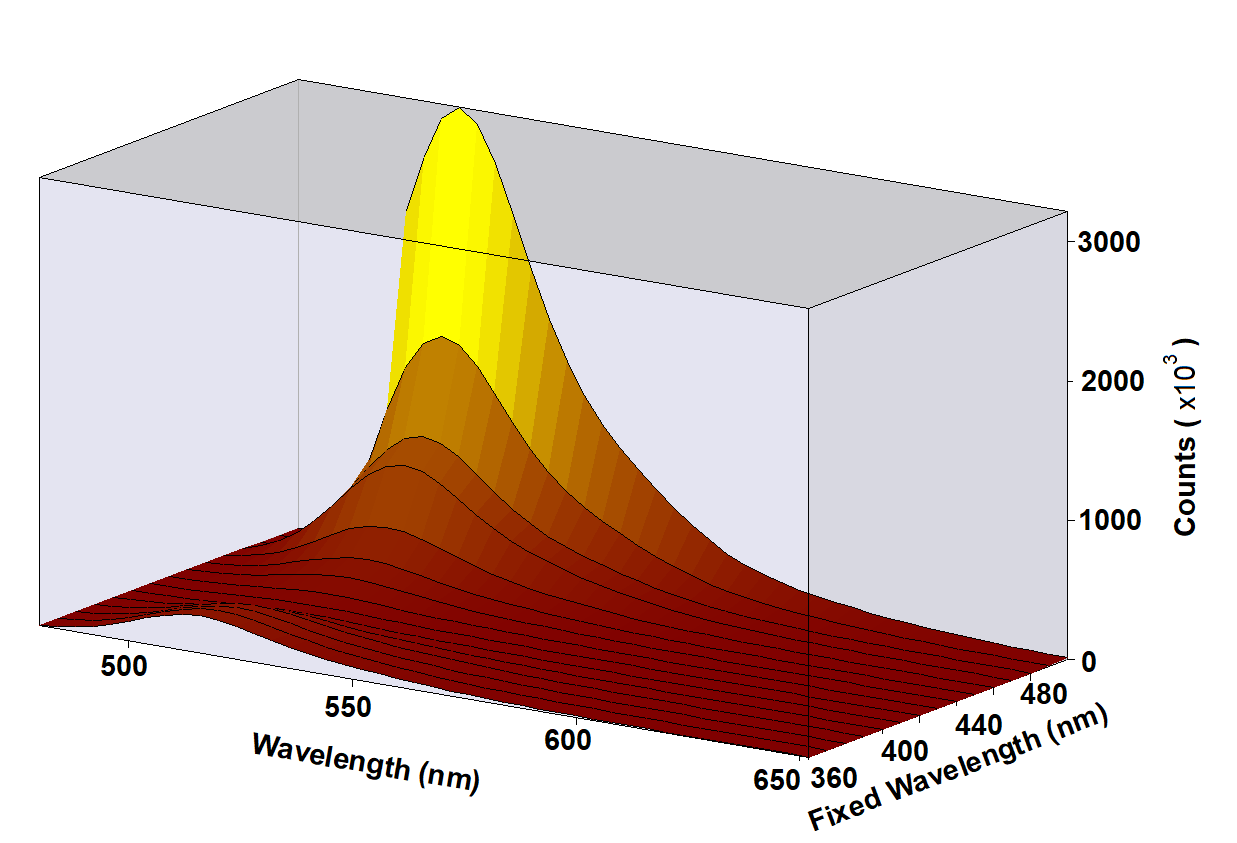


Figure S23. 3-D Emission map of monomer in dichloromethane, recorded at room temperature. Fixed wavelength axis shows varying excitation wavelengths used.

# Singlet oxygen measurements


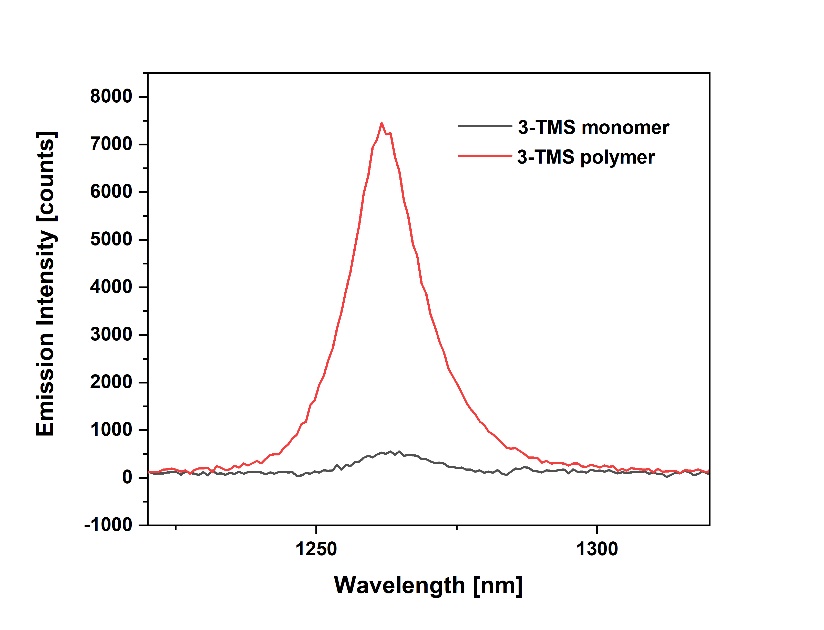


Figure S24. NIR Singlet oxygen emission spectra of monomer (grey spectra) and 3-TMS polymer (red spectra) in CHCl_3_ using λ_exc_ 530 nm. Ф_∆_ displayed in Table S2.

# Lifetime measurements


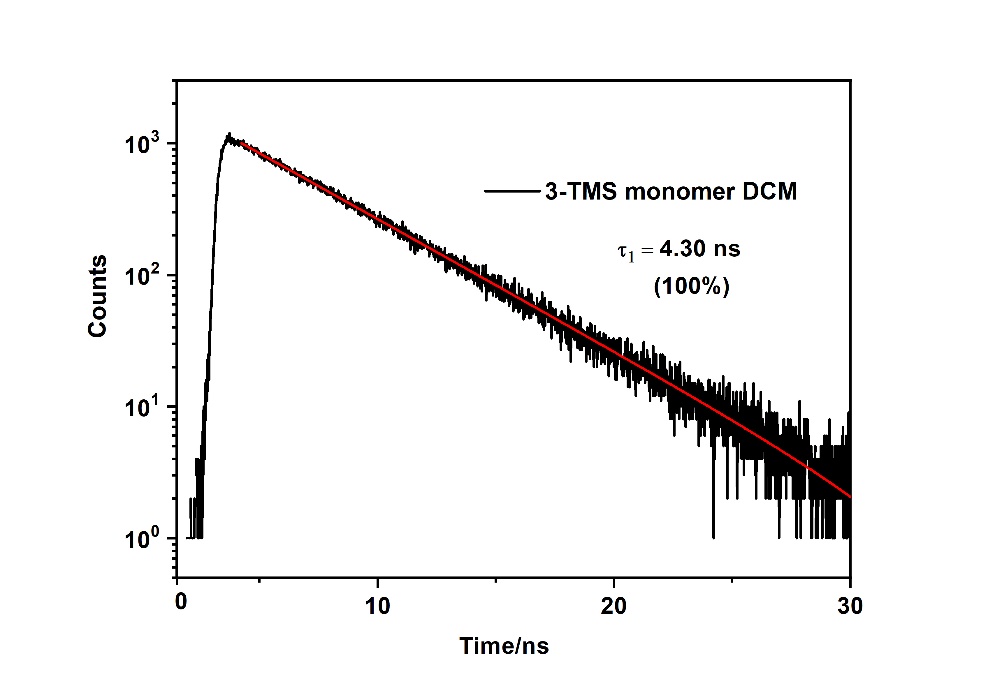


Figure S25. Emission decay of monomer in dichloromethane, λ_exc_ = 510 nm. Solution purged with N_2_ for 20 minutes prior to sample measurement. Red line showing exponential fit.


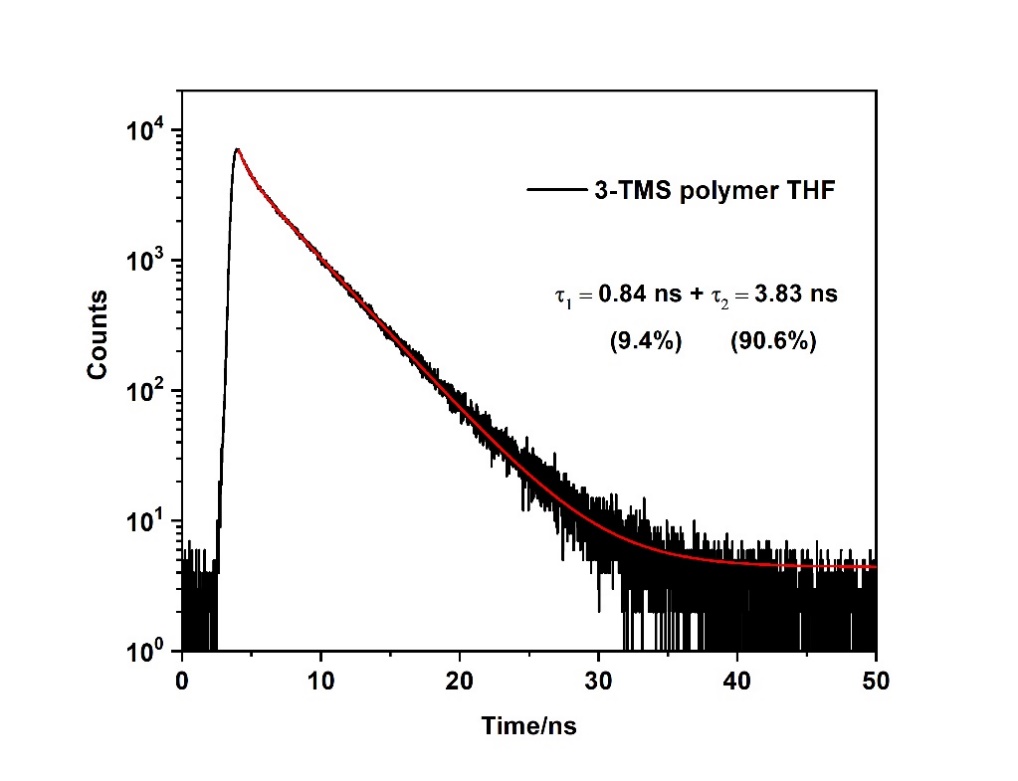


Figure S26. Emission decay of polymer in THF, λ_exc_ = 510 nm. Solution purged with N_2_ for 20 minutes prior to sample measurement. Red line showing biexponential fit.


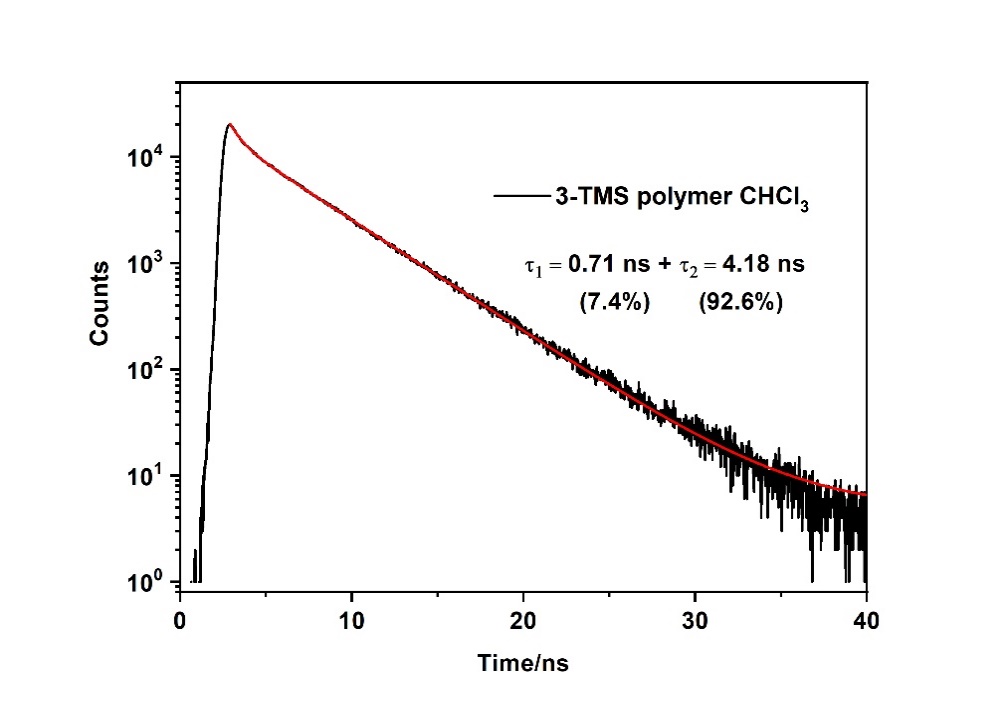


Figure S27. Emission decay of polymer in CHCl_3_, λ_exc_ = 510 nm. Solution purged with N_2_ for 20 minutes prior to sample measurement. Red line showing biexponential fit.


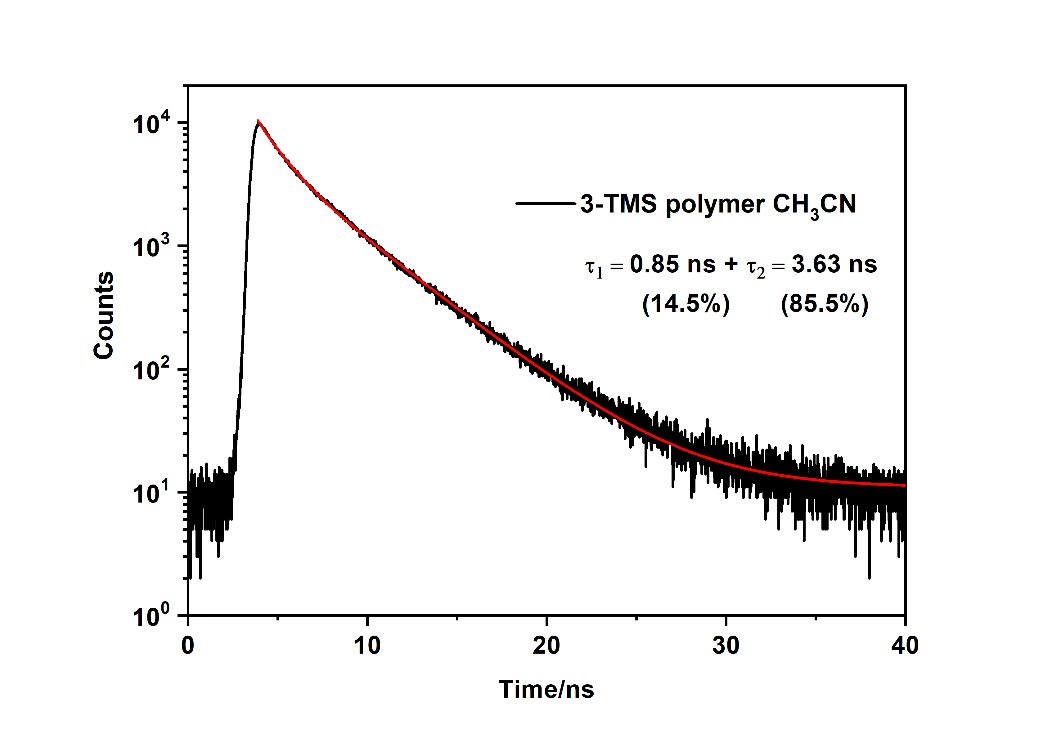


Figure S28. Emission decay of polymer in CH_3_CN, λ_exc_ = 510 nm. Solution purged with N_2_ for 20 minutes prior to sample measurement. Red line showing biexponential fit.


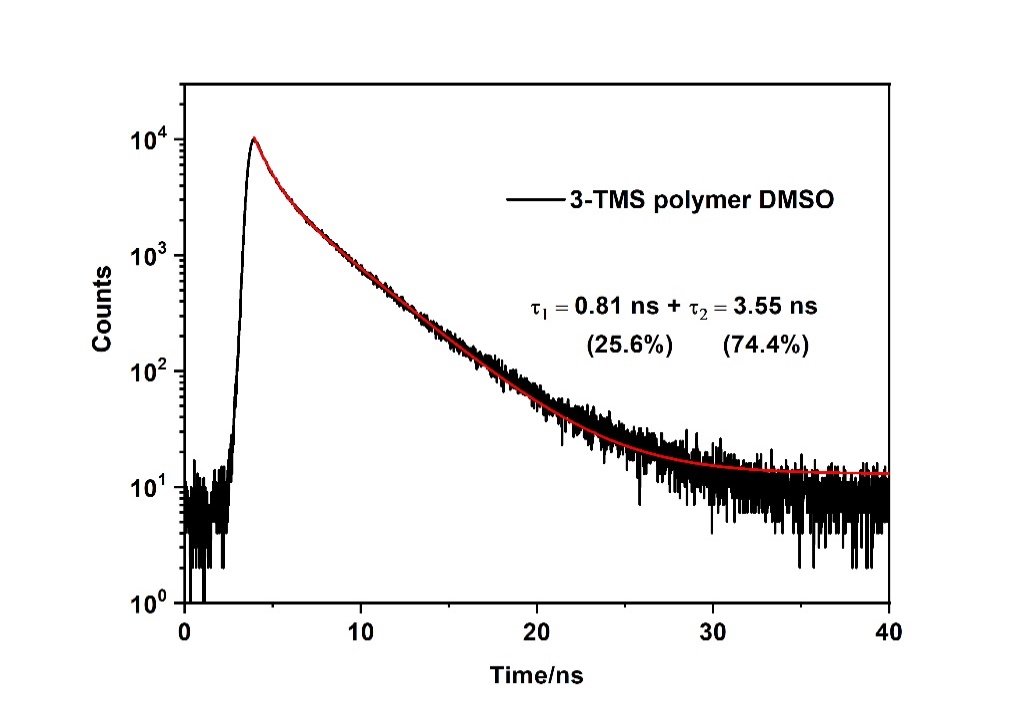


Figure S29. Emission decay of polymer in DMSO, λ_exc_ = 510 nm. Solution purged with N_2_ for 20 minutes prior to sample measurement. Red line showing biexponential fit.

# Optical gap determination


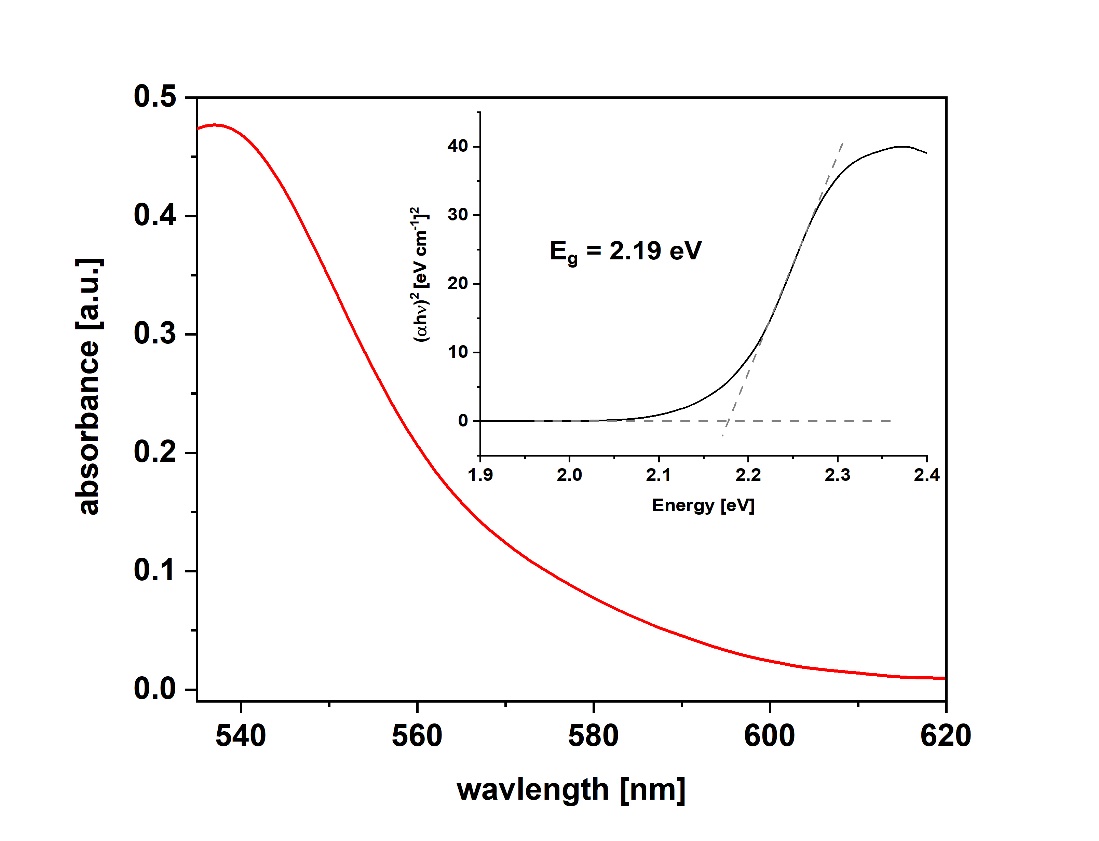


Figure S30. UV-visible absorption spectra of polymer in CH_2_Cl_2_. Inset showing Tauc Plot used to calculate the optical gap from the onset of absorption.^3,4^

# Steady state UV-visible absorption and emission spectra

3-TMS monomer and 3-TMS diiodo monomer UV-visible and emission spectrum can be seen in **Figure S26** and corresponding absorption and emission maxima summarized in **Table S1**.


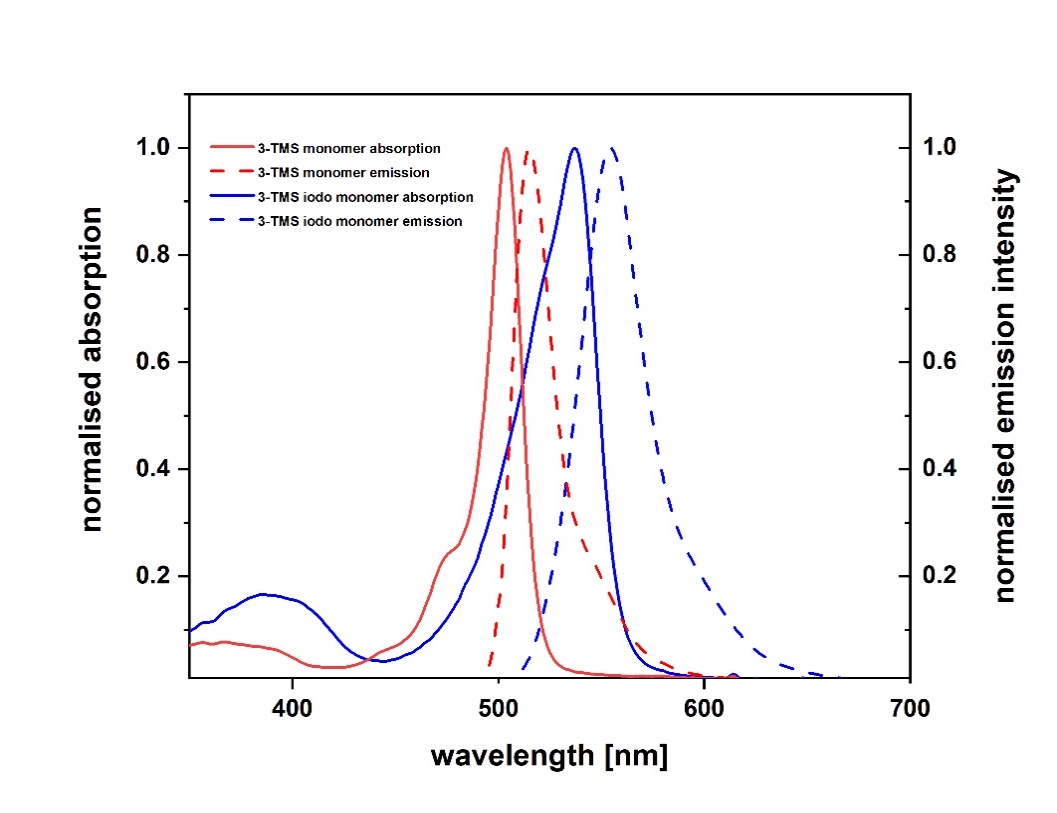


Figure S31. Normalised UV-visible absorption and emission spectra for monomer absorption (solid red), emission (dashed red), diiodo monomer absorption (solid blue) and emission (dashed blue). Recorded in CHCl_3_.

Table S1. Summary of photophysical properties monomer and diiodo monomer in CHCl_3_.

| **Compound** | **λ_abs_ (nm)** | **λ_em_ (nm)** |
| --- | --- | --- |
| monomer | 503 | 516 |
| diiodo monomer | 539 | 573 |

# Transient absorption spectra (ps-timescale)


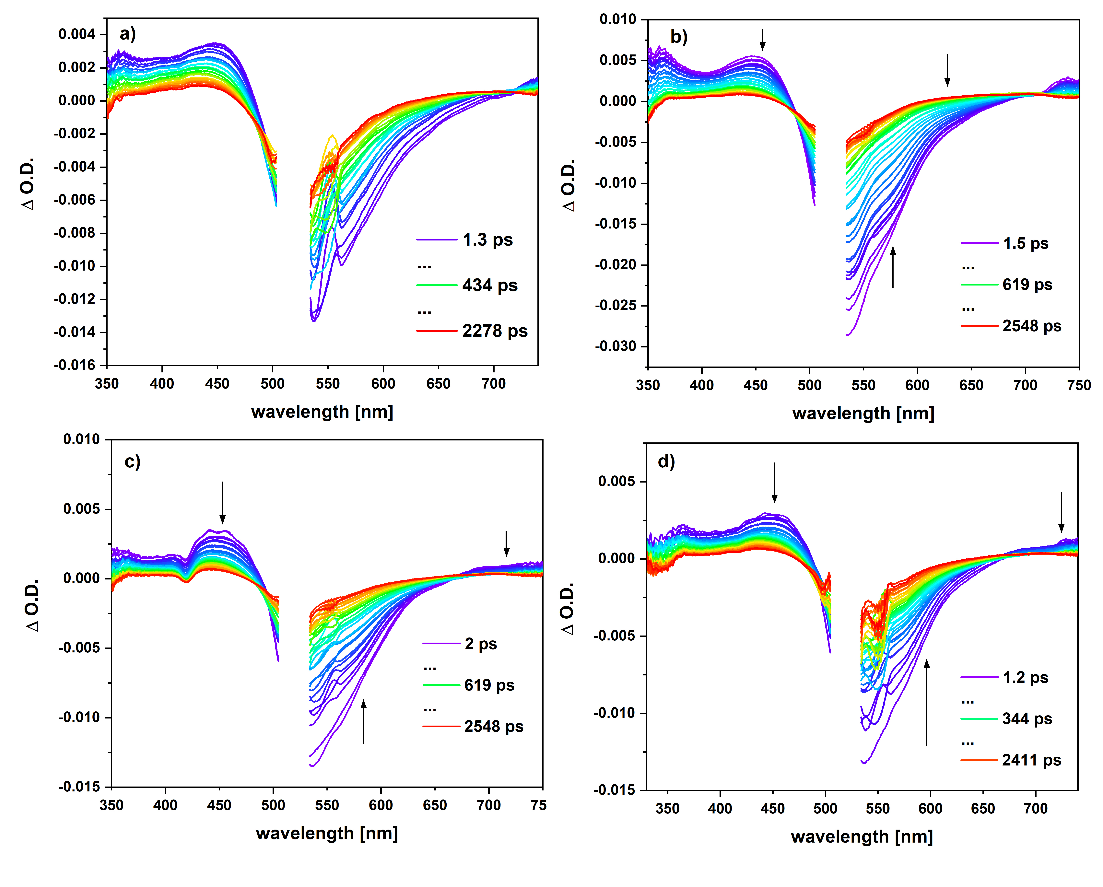


Figure S32. Transient absorption spectra of polymer in a) CD_3_CN, b) DMSO, c) chloroform and d) dichloromethane. Time delays indicated in inset. λ_exc_ = 525 nm.


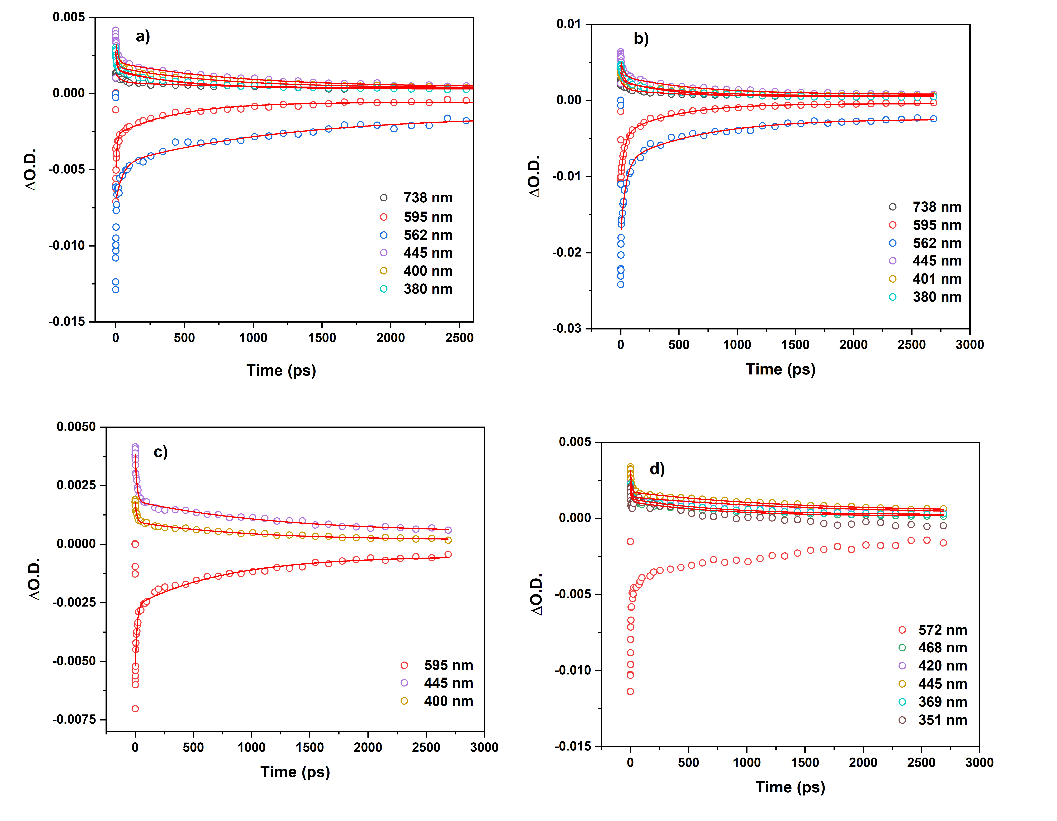


Figure S33. Exponential fitting (red curve) of polymer TA spectra at stated wavelengths in a) CD_3_CN, b) DMSO, c) chloroform and d) dichloromethane. Red line indicates exponential decay.


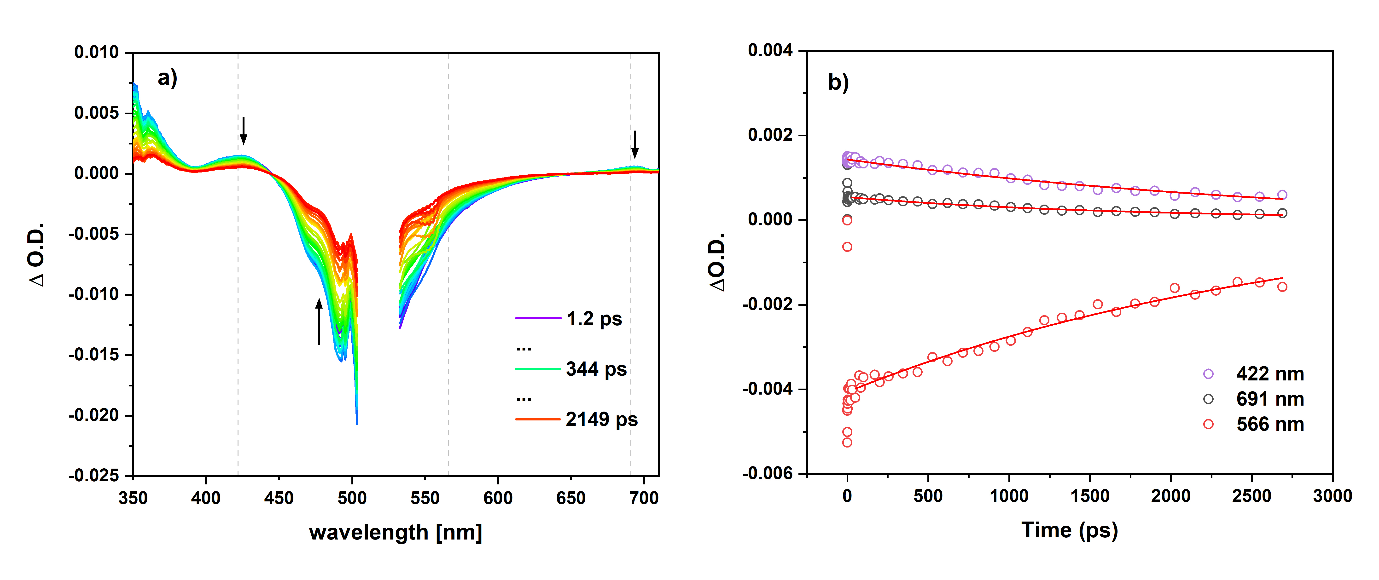


Figure S34. Transient absorption spectra of a) monomer in CD_3_CN at random time delays. Notch in graph where excitation wavelength occurred, grey dashed lines indicate the kinetic traces analysis and b) kinetic traces at stated wavelength, red lines indicate the exponential fit used, λ_exc_ = 525 nm.


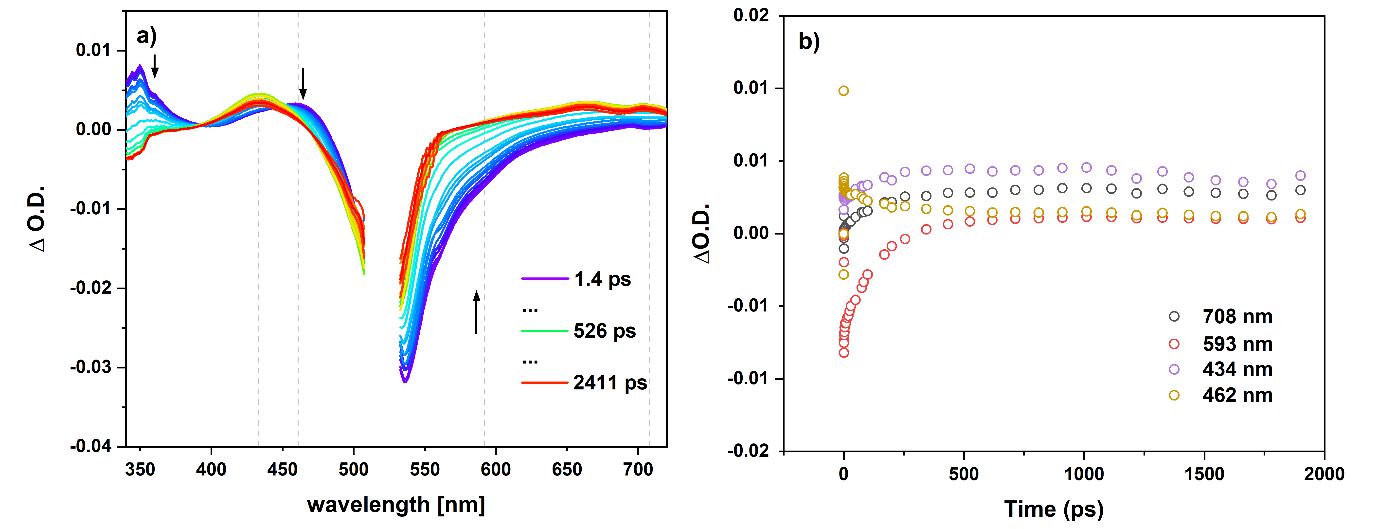


Figure S35. Transient absorption spectra of a) diiodo monomer in CD_3_CN at random time delays. Notch in graph where excitation wavelength occurred, grey dashed lines indicate the kinetic traces analysis and b) kinetic traces at stated wavelength, λ_exc_ = 525 nm.

# Transient absorption spectroscopy (ns-timescale)


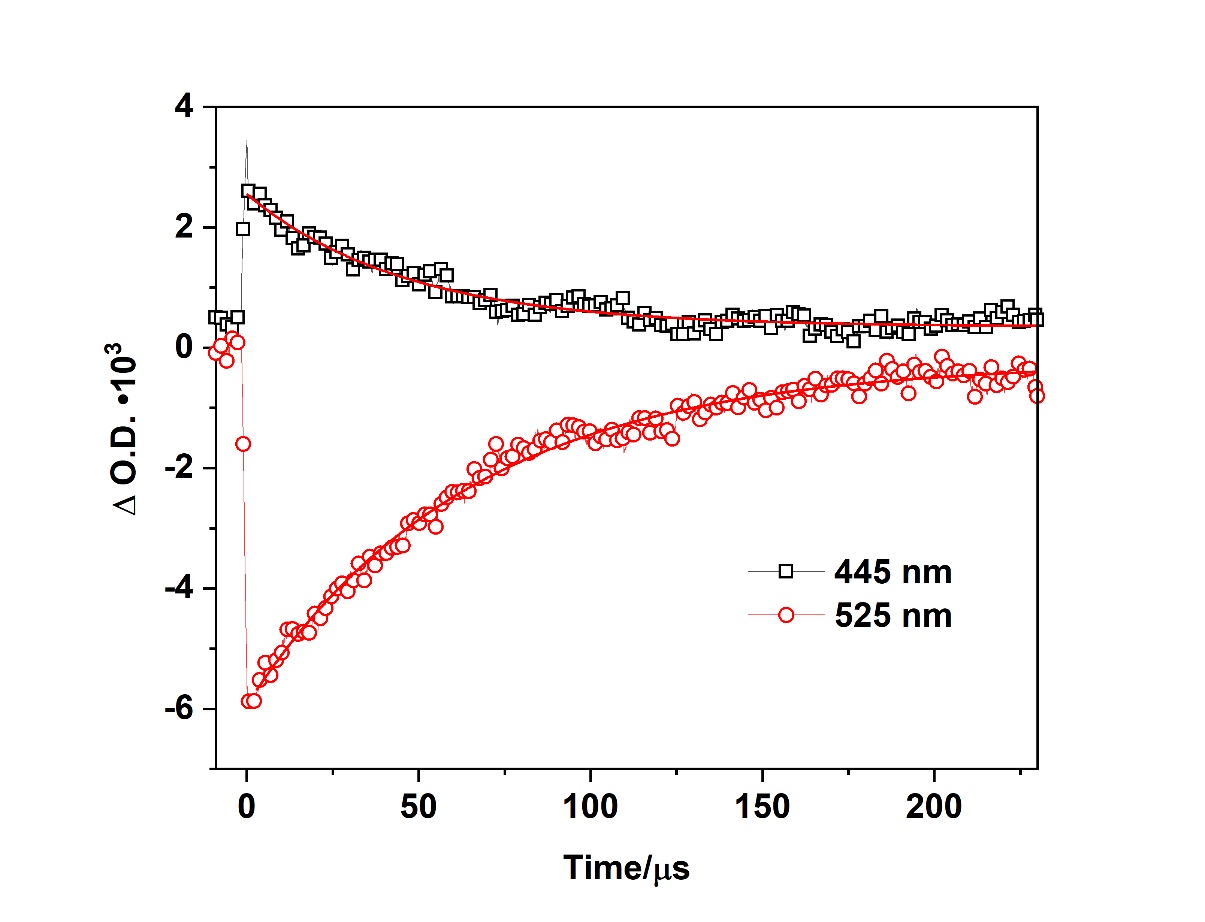


Figure S36. Transient absorption decays at stated wavelengths for 3-TMS polymer in CH_3_CN at stated wavelengths: 445 nm (black squares) and 525 nm (red circles). λ_exc_ = 355 nm. Red lines indicate exponential fit.

For monoexponential fitting the formula used is shown in equation **(1)**.

$\mathbf{y=}\mathbf{y}_{\mathbf{0}}\mathbf{+}\mathbf{A}_{\mathbf{1}}\mathbf{e}^{\frac{\mathbf{-x}}{\boldsymbol{\tau}_{\mathbf{1}}}}$ **(1)**

# Time Resolved Infrared Spectroscopy (ps-timescale)


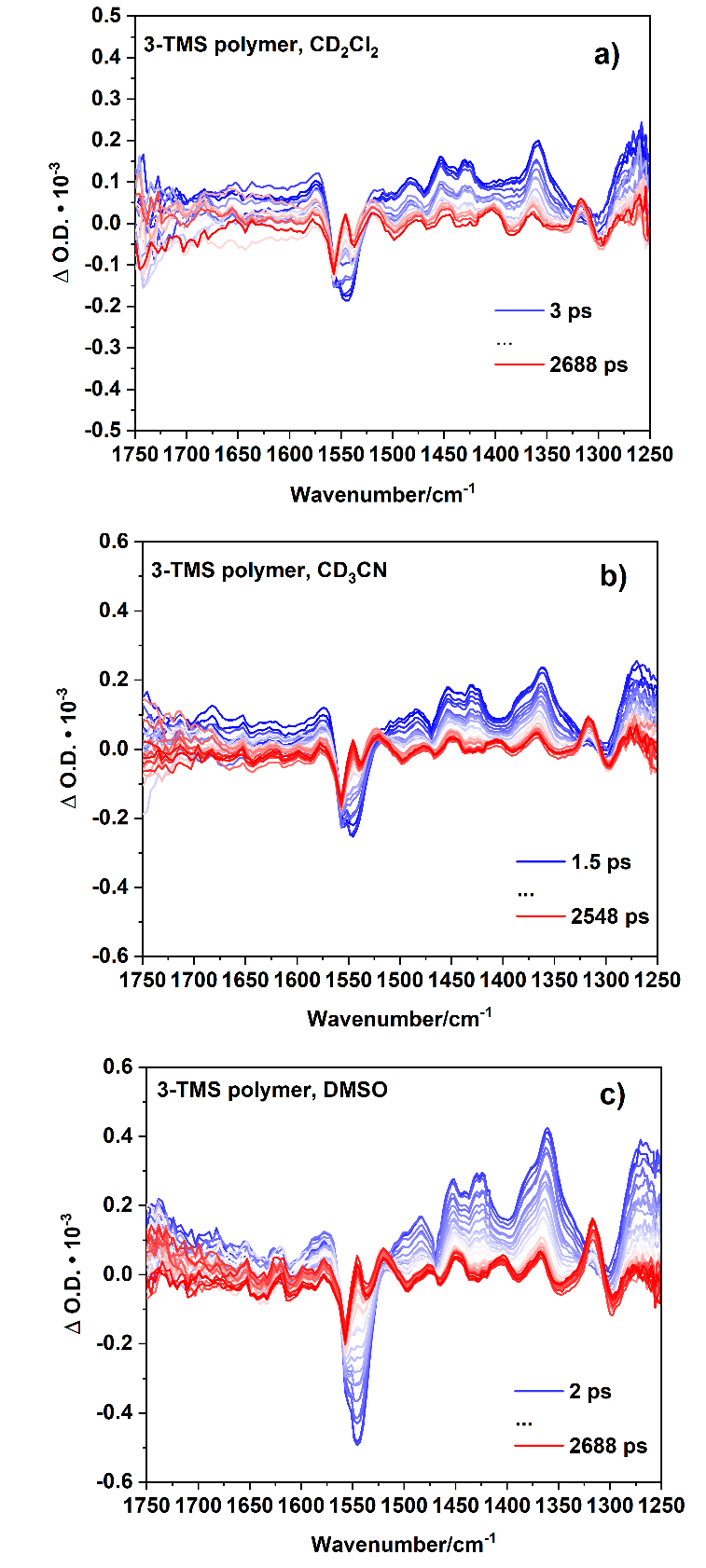


Figure S37. TRIR spectra of 3-TMS polymer in a) CD_2_Cl_2_ (top), b) CD_3_CN (centre) and c) DMSO (bottom) in the IR spectral window of 1750 cm^-1^ - 1250 cm^-1^. Blue spectra indicating initial time delays (ps), red spectra indicating final time delays (ps), λ_exc_ = 525 nm.


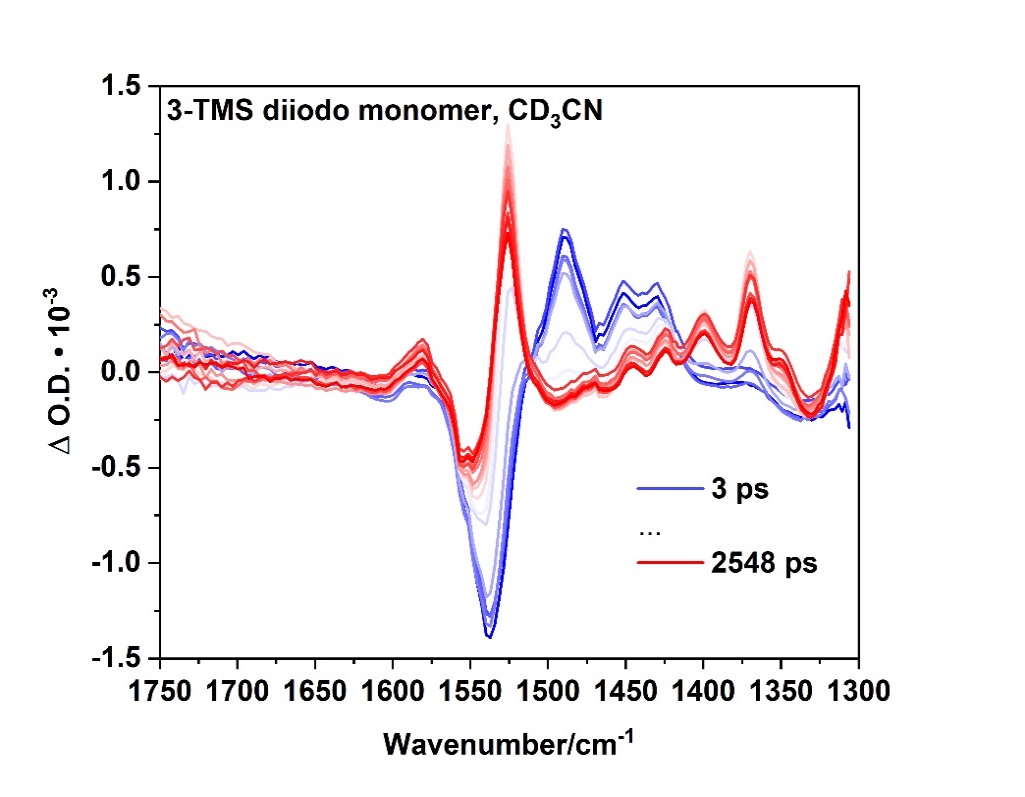


Figure S38. TRIR of 3-TMS diiodo monomer in CD_3_CN showing the spectral window of 1750 cm^-1^ – 1300 cm^-1^ in the IR region. Blue spectra indicating initial time delays (ps), red spectra indicating final time delays (ps), λ_exc_ = 525 nm.


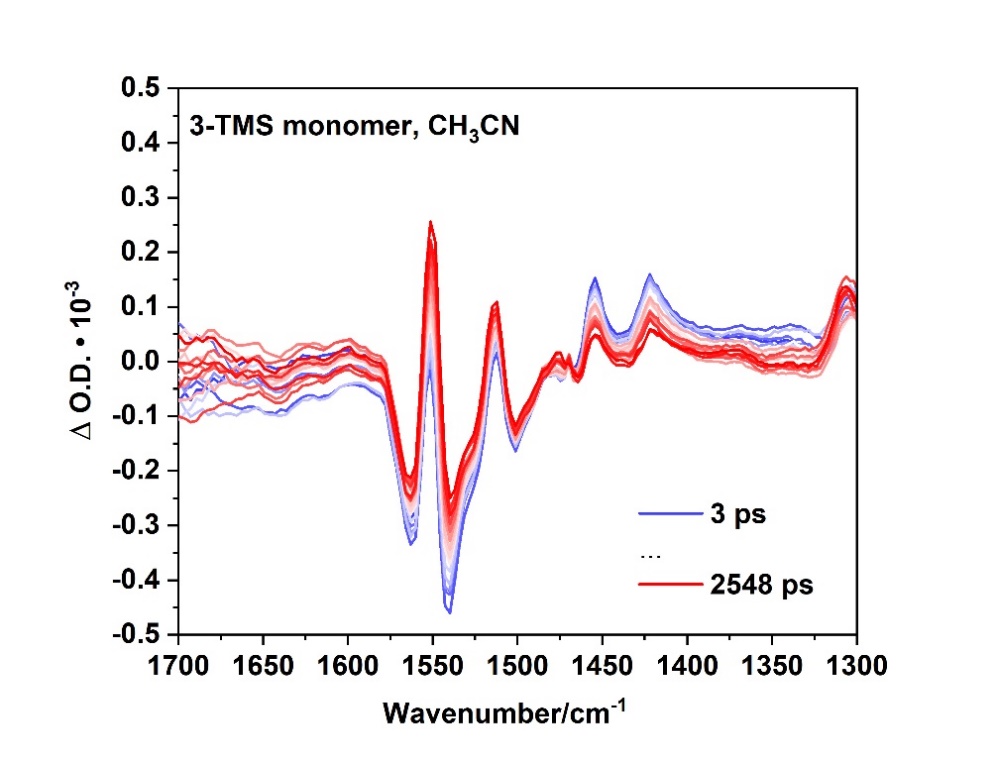


Figure S39. TRIR spectra of 3-TMS monomer in CH3CN showing the spectral window of 1750 cm^-1^ – 1300 cm^-1^ approximately in the IR region. Blue spectra indicating initial time delays (ps), red spectra indicating final time delays (ps), λ_exc_ = 525 nm.


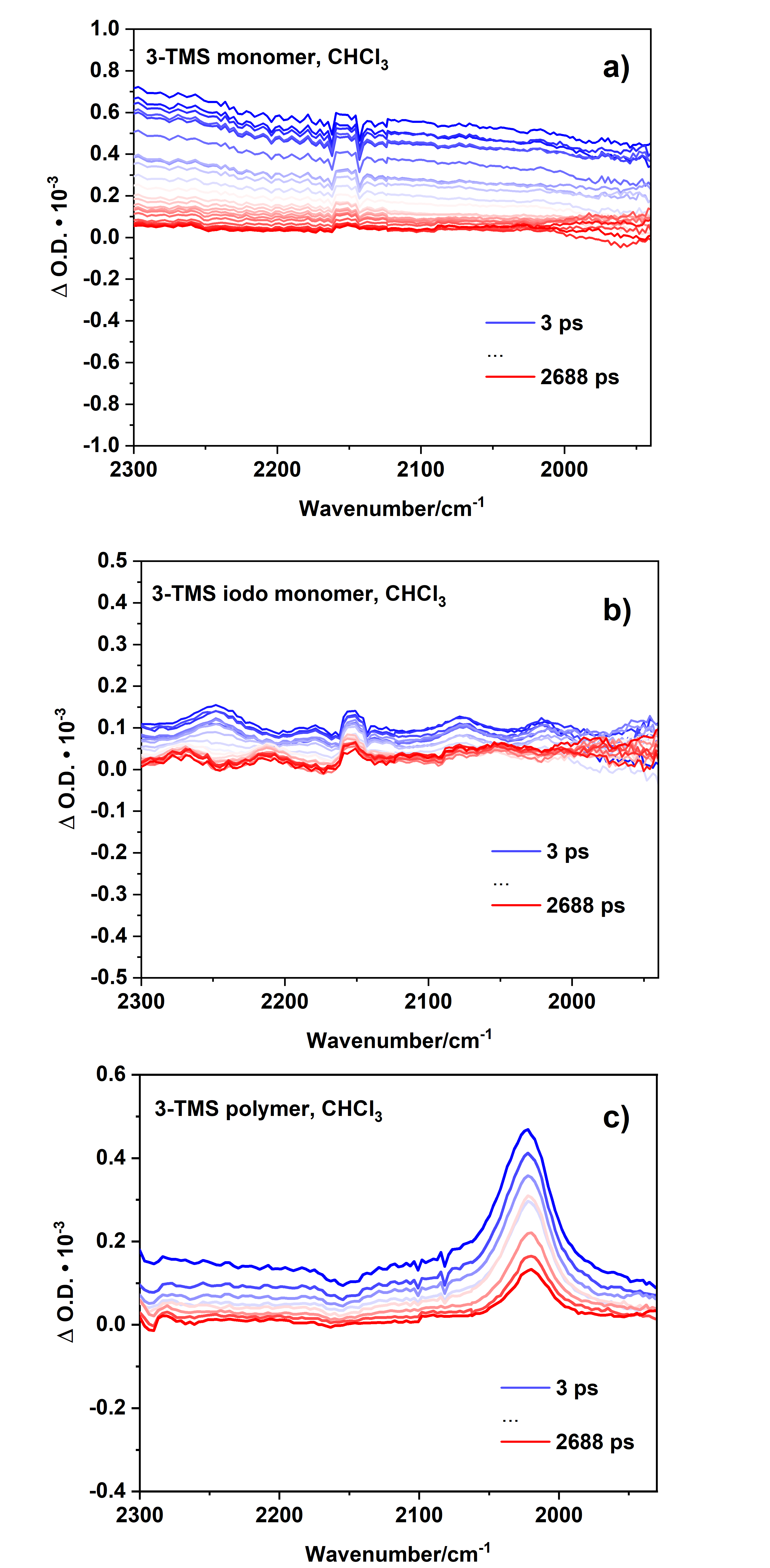


Figure S40. TRIR spectra of a) 3-TMS monomer, b) 3-TMS diiodo monomer and c) 3-TMS polymer in CHCl_3_, showing the spectral window of 2300 cm^-1^ – 1930 cm^-1^ in the IR region. Blue spectra indicating initial time delays (ps), red spectra indicating final time delays (ps), λ_exc_ = 525 nm.

# Summary of photophysical properties

Table S2. Summary of photophysical properties of 3-TMS monomer and 3-TMS polymer. ^a^measured in CH_2_Cl_2_, ^b^ approx. molar absorptivity of the polymer calculated using the molecular weight of the monomeric repeating unit, ^c^Full Width at Half Maximum of stated absorption or emission band, ^d^flourescence quantum yield calculated using 3-pyridine H-BODIPY as a reference standard, where Φ_fl_ = 62% in CH_2_Cl_2,_^1^ ^e^measured using TCSPC, ^f^the chi-squared value for determining the accuracy of the decay fit, ^g^determined from the decay kinetics obtained from ns-transient absorption spectroscopy in a sample that was degassed using freeze-pump thaw method, ^h^singlet oxygen quantum yield calculated using ZnTPP as a reference standard, where Ф_∆_ = 0.72 in CHCl_3_.^5^ ^i^the optical gap determined from the absorption edge.

| **Compound** | **λ_abs_ (nm)^a^ (ԑ, 10^3^ M^-1^ cm^-1^)^b^** | **λ_em_ (nm)^a^** | **∆ʋ (cm^-1^)** | **FWHM_abs_^c^ (cm^-1^)** | **FWHM_em_^c^ (cm^-1^)** | **Ф_fl_^d^** | **τ_s_ (ns)^e^** | **χ^2 f^** | **τ_T_ (μs)^g^** | **Ф_∆_^h^** | **Optical gap (eV)^i^** |
| --- | --- | --- | --- | --- | --- | --- | --- | --- | --- | --- | --- |
| monomer | 503 (63) | 516 | 501 | 768 | 857 | 0.92 | 4.3 | 1.1 | - | 0.05 | 2.41 |
| polymer | 539 (17) | 573 | 1101 | 2209 | 2363 | 0.09 | 1.1, 3.9 | 1.2 | 61 | 0.77 | 2.19 |

# Photocatalytic hydrogen evolution experiments in solution

Hydrogen experiments were carried out as per previously reported by the group.^6–8^ In brief, all photocatalytic experiments to assess the activity of the polymer for hydrogen evolution were carried out in 23 mL Schlenk tubes, with 8 mL volume of photocatalytic solution. All Schlenk tubes were equipped with an air-tight septum to allow samples of headspace to be measured at different timepoints throughout the experiment. Degassing of photocatalytic solution was carried out using three freeze-pump-thaw cycles to ensure oxygen was not present during photocatalysis. The Schlenk tube was blocked to external light prior to irradiation to ensure only the Xe arc lamp incident on the photocatalytic tube was the cause of the photocatalytic activity. For hydrogen experiments employing 0.1 M ascorbic acid, the appropriate amount of 0.2 M NaOH solution was titrated into the solution to obtain the desired pH prior to addition to the reaction Schlenk tube containing the polymer and the catalyst components of the desired concentration. The Schlenk tube was placed in a black box equipped with a fan to avoid thermal processes driving the catalytic cycle. All samples were irradiated with 300 W Xe arc lamp equipped with cut-off filter, λ > 420 nm. Control samples were left in the dark and measured at the same timepoints as described, no hydrogen was observed. Hydrogen production was monitored by gas chromatography (Shimadzu GC-2010) with a BID detector on a 5 Å molecular sieve column using He as a carrier gas, with oven temperature at 30 ºC. Hydrogen obtained was measured against a calibration curve of standard injections, using commercially available standards of 0.01%, 0.1%, 1%, 2% and 5% respectively. **Figure S36** depicts the hydrogen evolution results obtained using the polymer as a photosensitiser in this study with specific conditions summarized in **Table S3**.


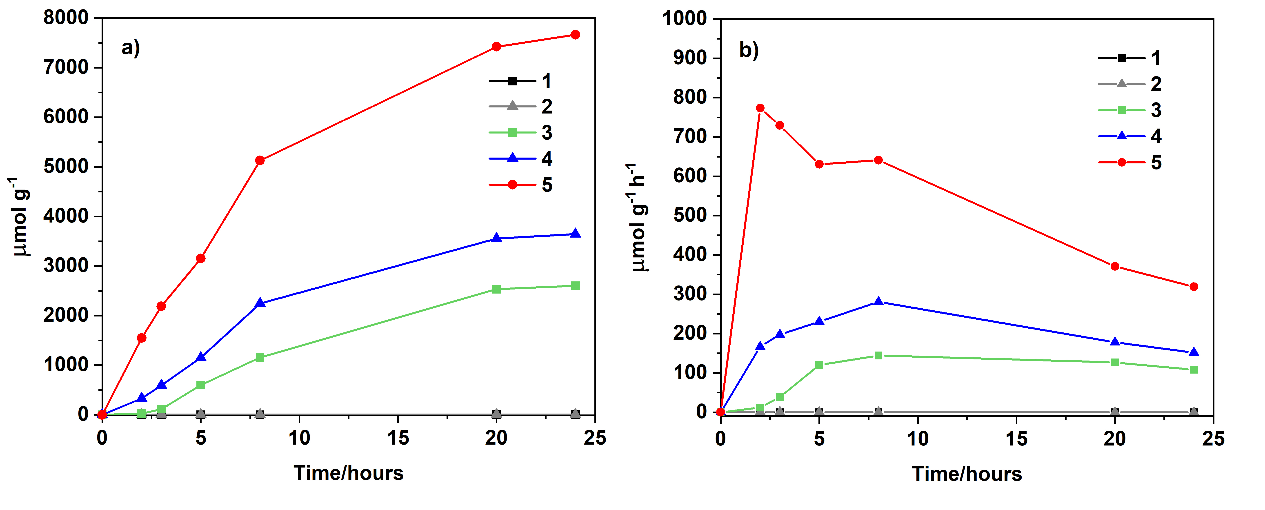


Figure S41. a) Hydrogen evolution of the polymer over 24 hours and b) corresponding turnover frequencies. Conditions summarised in Table S3 for 1-5.

Table S3. Summary of experimental parameters using polymer as PS and cobalt cobaloxime as catalyst, irradiation Xe arc lamp, λ > 420 nm. SA – sacrificial agent, TEA – triethylamine, AA – ascorbic acid. A parameter altered in every subsequent experiment is shown in red. ^a^all solvent systems were prepared in a 1:1 (v/v) solvent ratio unless otherwise stated, ^b^refers to the pH of the ascorbic acid solution prior to addition to the photocatalytic Schlenk tube containing the polymer and catalyst dissolved in the stated organic solvent.

| **Entry** | **mass polymer (mg)** | **concentration catalyst (mM)** | **solvent system^a^** | **SA (concentration)** | **pH** | **µmol g^-1^** | **µmol h^-1^ g^-1^** |
| --- | --- | --- | --- | --- | --- | --- | --- |
| 1 | 0.5 | 1.8 | ACN: H_2_O, 9: 1 (v/v) | TEA (2.2 M) | 8.5 | 2 | 0.1 |
| 2 | 0.5 | 2.5 | ACN: H_2_O | AA (0.1 M) | 2^b^ | 3 | 0.2 |
| 3 | 0.5 | 2.5 | ACN: H_2_O | AA (0.1 M) | 5^b^ | 2602 | 108 |
| 4 | 0.5 | 2.5 | THF: H_2_O | AA (0.1 M) | 5^b^ | 3645 | 152 |
| 5 | 2 | 2.5 | THF: H_2_O | AA (0.1 M) | 5^b^ | 7664 | 319 |


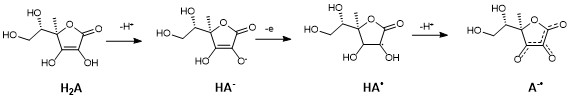


Figure S42. Ascorbic acid showing multiple deprotonation steps and monooxidation step in different pH environments.^9^

# Photoelectrochemical Hydrogen Evolution using NiO Photocathodes

**Preparation of NiO Films.** Mesoporous NiO films were prepared according to a previously reported procedure.^10^ Precursor solution comprised anhydrous NiCl_2_ and tri-block co-polymer F108 (poly (ethylene glycol)-block-poly (propylene glycol)-block-poly (ethylene glycol)). 1 g of each was dissolved in ethanol (6 mL) and ultrapure Milli-Q water (5 mL). The resulting solution was left to age in a vial for 1-2 weeks and centrifuged to remove any large aggregated polymer before doctor blading onto cleaned conductive glass, fluorine doped tin oxide (FTO)-coated TEC^TM^ 15 (Pilkington), cut into 2x2 cm squares. The area of the NiO was 0.79 cm^-1^, masked off by Scotch Magic Tape.

Three layers of NiO were deposited for each film, annealing each layer in a furnace at 450 ^o^C for 30 mins in the presence of oxygen. BODIPY 3-TMS polymer sensitizer was adsorbed onto the NiO surface by chemical bath deposition where dye was dissolved in dry acetonitrile (0.3 mM) and films submerged in a sealed container for at least 16 hours (overnight). Film thickness was 1.5 μm on average, determined by surface profilometry (Bruker Dektak^3^XT Surface Profile Measuring System). Pt counter electrodes were prepared by dissolving chloroplatinic acid on 2x2 TEC 8 (Pilkington) FTO glass films and annealing in the presence of oxygen at 150 ^o^C for 15 mins.

**Preparation of co-catalyst*.*** (4’4-dicarboxy-2’2-bipyridine)dichloroplatinum (II) was prepared by modification of a previously reported procedure.^11^ 4,4’-dicarboxy-2,2’-bipyridine with K_2_PtCl_4_ (Sigma) were refluxed in water for one hour at 80 degrees Celsius to give the Pt-bipyridine complex (named Pt-bipy for convenience). The filtered product was washed and recrystallized in acetonitrile. The compound was sparingly soluble in alcohols. Characterization was by high-resolution mass spectrometry.


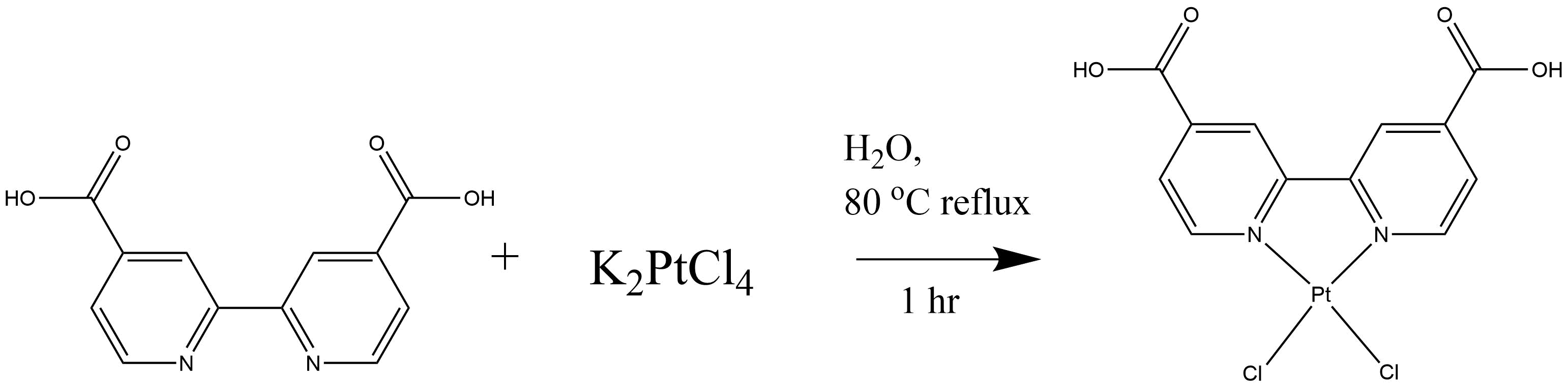


**Figure S43.** Reaction scheme for synthesis of (4'4-dicarboxy-2'2-bipyridine)dichloroplatinum(II).

**Electrochemistry.** Electrochemical measurements were conducted in a three-electrode cell where the NiO films with the adsorbed sensitizer and catalyst were the working electrode, a Pt-coated FTO glass film the counter electrode, and the reference electrode Ag/AgCl (3.0 M NaCl, E_o_ Ag/AgCl = 0.210 V vs. NHE). Potential was applied with an IviumStat EmStat 3 Blue potentiostat.

Before each photoelectrocatalysis experiment, the reaction cell was degassed for at least 15 minutes with either nitrogen or argon. Aqueous electrolytes were freshly prepared and pH adjusted with concentrated HCl, and measured with a benchtop pH meter (Hanna Instruments). The cell was irradiated with simulated 1 sun intensity light (AM 1.5, 100 mW cm^-2^) using a 300 W Xe arc lamp (Oriel Instruments).

**Analysis of Gases.** Gas chromatography measurements were carried out using a Shimadzu GC-2014 instrument fitted with a ShinCarbon ST Micropacked column (Restek) using Ar as a carrier gas at 25 ml min^-1^. Gases were detected with a thermal conductivity detector (TCD) with the system operating at 80 ^o^C. Gas sampling was done in flow, through a integrated cell block (**Figure S39**), where research grade argon was used as a carrier gas to a 2-position 6-port Rheodyne switch at 1 SCCM maintained by a Bronkhorst E-flow series mass flow controller. The flow setup was programmed to run automated analysis every 3 minutes. Calibration of hydrogen measurements were undertaken by dosing known amounts of pure H_2_ (99.9995%) into the GC in flow dosing via a second 2-position, 6-port Rheodyne switch. All measurements were repeated several times.


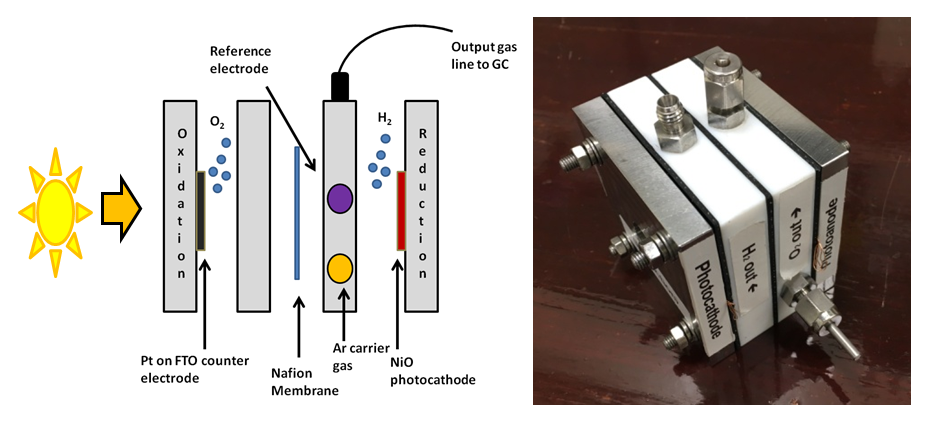


**Figure S44**. Photoelectrocatalysis cell block exploded view and photo of the device.

**Optical Measurements**. UV-visible absorption measurements for films were obtained using an Ocean Optics USB2000+ spectrophotometer where fibre optic cables enabled effective measurements through the film.


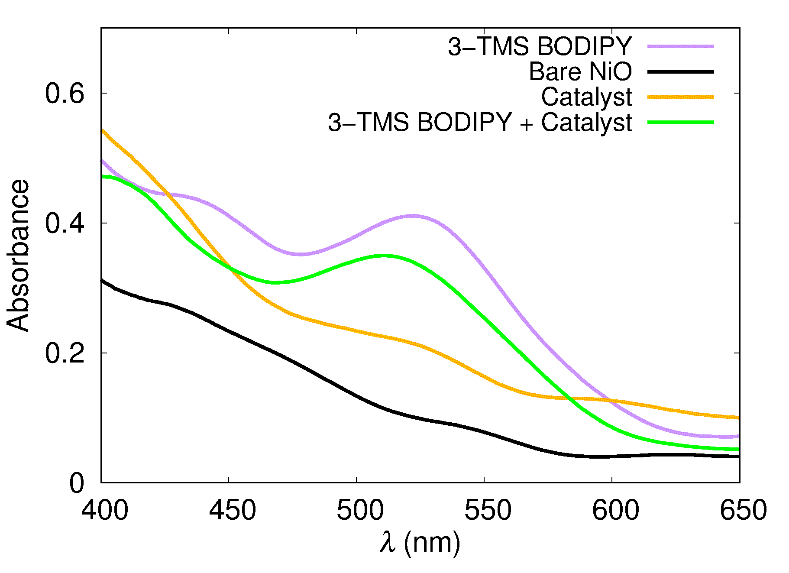


Figure S45. Steady-state absorption spectra of sensitised NiO films

**Microscopy**. Scanning electron microscopy was undertaken at Northumbria University (UK) using a Tescan Mira 3 microscope with an Oxford Instruments X-ray detector. Typical imaging conditions employed an in-beam SE detector with the electron source set at 5kV.


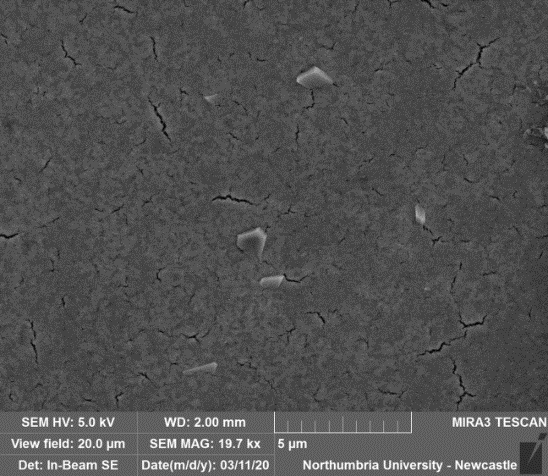


Figure S46. SEM image of the dye-sensitized NiO surface containing 3-TMS BODIPY polymer and Pt-bipy catalyst.


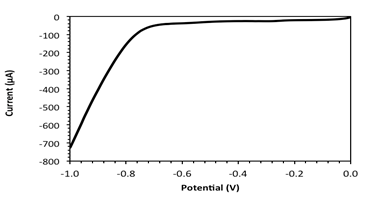


Figure S47. Linear sweep measurement (in the dark) for a NiO film sensitized with 3-TMS BODIPY polymer and Pt-bipy catalyst in pH 5 phthalate buffer vs Ag/AgCl. Scan speed 0.05 V/s.


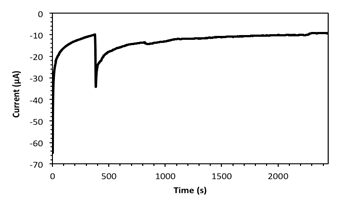


Figure S48. Chronoamperometry measurement of NiO sensitized with 3-TMS BODIPY polymer and Pt-bipy, in pH 5 phthalate buffer with an applied potential of -0.3 V vs Ag/AgCl. A period of equilibriation in the dark takes place till AM 1.5 1 sun illumination is introduced at approximately 380 seconds.


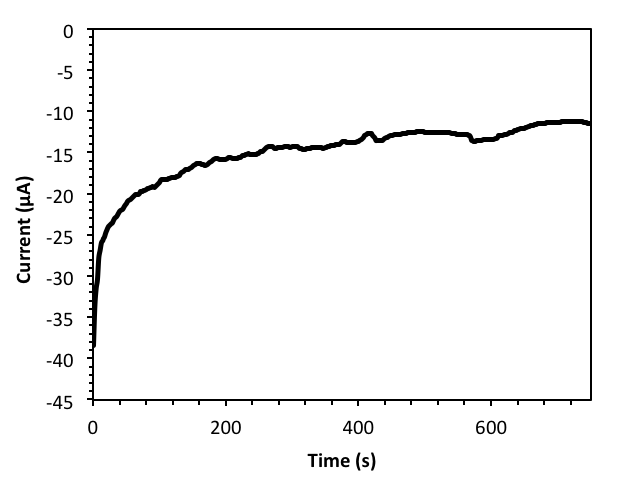


**Figure S49.** Control chronoamperometry experiment with bare NiO in pH 5 phthalate buffer with an applied potential of -0.3 V vs Ag/AgCl under 1 sun illumination. Chopping the light output had no impact on the current.

# Literature review of organic-based polymers for hydrogen evolution

To get an idea of the activity of other organic polymers for hydrogen evolution, we summarised the current activity reported for polymeric systems.

Table S4. Comparison of photocatalytic experimental conditions and hydrogen evolution rate (HER) of organic polymeric photocatalysts reported in the literature. COF – covalent organic framework, CMP – conjugated microporous polymer.

| **Polymer type** | **Conditions** | **Light source** | **Catalysts** | **H_2_ (µmol) (irradiation time)** | **H_2_ (µmol h^-1^) (irradiation time)** | **H_2_ (µmol h^-1^ g^-1^)** | **ref** |
| --- | --- | --- | --- | --- | --- | --- | --- |
| PPP | 20 mg polymer, 2 mL H20, 2 mL diethylamine | 300 (Hg) | - | 8.3 (4 hours) | - |  | ^12^ |
| Nitrogen-PPP (P31) | 25 mg polymer, water/methanol/triethylamine solution | 300 W Xe Arc, λ > 295 nm | - | - | 37.5 ± 1.1 |  | ^13^ |
| Nitrogen-PPP (P31) | 25 mg polymer, water/methanol/triethylamine solution | 300 W Xe Arc, λ > 420 nm | - | - | 15.2 ± 0.06 |  | ^13^ |
| Poly(axomethine) networks | 100 mg polymer, 100 mL aqueous solution with 10% vol TEOA | Xe Arc, λ > 300 nm | 3 wt% Pt | 26.7 | 7 |  | ^14^ |
| Hydrazone based COF | 4 mg polymer, 9mL H_2_O, 10% vol TEOA |  | 2.2 wt% Pt |  |  | 1970 | ^3^ |
| Hydrazone based COF | 10 mg polymer, 100 mg sodium ascorbate, 10 mL H_2_O | 300 W Xe Arc, λ > 420 nm | 2.2 wt% Pt | 97.6 (52 hours) |  | 230 | ^3^ |
| CP-CMP10 | 100 mg polymer, 100 mL diethylamine/water solution (20 vol%) | 300 W Xe Arc, λ > 420 nm |  | 100 (6 hours) | 17.4 ± 0.9 | - | ^15^ |
| Dibenzo-[b,d]-thiophene sulfone copolymer (P7) | 25 mg polymer, water/methanol/triethylamine solution | 300 W Xe Arc, λ > 295 nm |  |  | 145 ± 7 (5 hours) |  | ^16^ |
| Dibenzo-[b,d]-thiophene sulfone copolymer (P7) | 25 mg polymer, water/methanol/triethylamine solution | 300 W Xe Arc, λ > 420 nm |  |  | 92 ± 2 (5 hours) |  | ^16^ |
| Conjugated polybenzothiadiazole | 50 mg polymer, 10% vol TEOA, 110 mL H_2_O | 300 W Xe Arc, λ > 420 nm |  |  | 12 |  | ^17^ |
| Conjugated polybenzothiadiazole | 50 mg polymer, 10% vol TEOA, 110 mL H_2_O | 300 W Xe Arc, λ > 420 nm | 3 wt% Pt |  | 116 |  | ^17^ |
| P8-i | 25 mg polymer, water/methanol/triethylamine solution | 300 W Xe Arc, λ > 295 nm |  |  | 21.5 ± 0.1 (5 hours) |  | ^18^ |
| P8-i | 25 mg polymer, water/methanol/triethylamine solution | 300 W Xe Arc, λ > 420 nm |  |  | 3.1 ± 0.02 (5 hours) |  | ^18^ |
| P8-s | 25 mg polymer, water/methanol/triethylamine solution | 300 W Xe Arc, λ > 295 nm |  |  | 2.0 ± 0.05 (5 hours) |  | ^18^ |
| P8-s | 25 mg polymer, water/methanol/triethylamine solution | 300 W Xe Arc, λ > 420 nm |  |  | 0.5 ± 0.05 (5 hours) |  | ^18^ |
| Perylene CMP | 100 mg polymer, 100 mL 20% vol TEOA aqueous solution | 300 W Xe Arc, λ > 300 nm | 3 wt% Pt |  | 12.1 |  | ^19^ |

# Compounds in solution


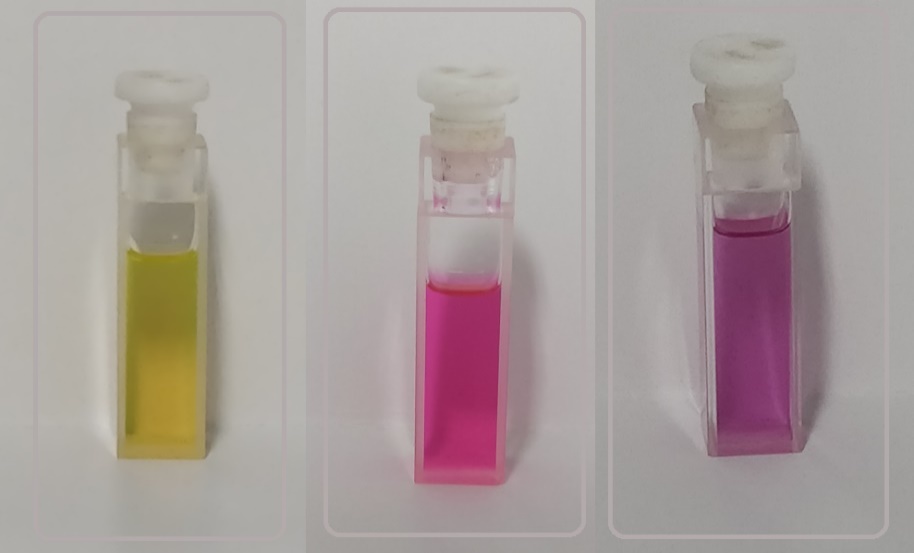


Figure S50. Quartz cuvettes showing monomer (left), diiodo monomer (centre) and polymer (right) in CH_2_Cl_2_, aerated solution.

# References

(1) Banfi, S.; Nasini, G.; Zaza, S.; Caruso, E. *Tetrahedron* **2013**, *69* (24), 4845–4856.

(2) Du, P.; Schneider, J.; Luo, G.; Brennessel, W. W.; Eisenberg, R. *Inorg. Chem.* **2009**, *48* (11), 4952–4962.

(3) Stegbauer, L.; Schwinghammer, K.; Lotsch, B. V. *Chem. Sci.* **2014**, *5* (7), 2789–2793.

(4) Yu, J.; Sun, X.; Xu, X.; Zhang, C.; He, X. *Appl. Catal. B Environ.* **2019**, *257* (March), 117935.

(5) Redmond, R. W.; Gamlin, J. N. *Photochem. Photobiol.* **1999**, *70* (4), 391–475.

(6) O’Reilly, L.; Pan, Q.; Das, N.; Wenderich, K.; Korterik, J. P.; Vos, J. G.; Pryce, M. T.; Huijser, A. *ChemPhysChem* **2018**, *19* (22), 3084–3091.

(7) Das, N.; Bindra, G. S.; Paul, A.; Vos, J. G.; Schulz, M.; Pryce, M. T. *Chem. - A Eur. J.* **2017**, *23* (22), 5330–5337.

(8) Manton, J. C.; Long, C.; Vos, J. G.; Pryce, M. T. *Phys. Chem. Chem. Phys.* **2014**, *16* (11), 5229–5236.

(9) Pellegrin, Y.; Odobel, F. *Comptes Rendus Chim.* **2017**, *20* (3), 283–295.

(10) Sumikura, S.; Mori, S.; Shimizu, S.; Usami, H.; Suzuki, E. *J. Photochem. Photobiol. A Chem.* **2008**, *199* (1), 1–7.

(11) Morgan, T. G.; Burstall, H. F. *J. Chem. Soc.* **1934**, *323*, 1498–1500.

(12) Yanagida, S.; Kabumoto, A.; Mizumoto, K.; Pac, C.; Yoshino, K. *J. Chem. Soc. Chem. Commun.* **1985**, No. 8, 474–475.

(13) Sprick, R. S.; Wilbraham, L.; Bai, Y.; Guiglion, P.; Monti, A.; Clowes, R.; Cooper, A. I.; Zwijnenburg, M. A. *Chem. Mater.* **2018**, *30* (16), 5733–5742.

(14) Schwab, M. G.; Hamburger, M.; Feng, X.; Shu, J.; Spiess, H. W.; Wang, X.; Antonietti, M.; Müllen, K. *Chem. Commun.* **2010**, *46* (47), 8932–8934.

(15) Sprick, R. S.; Jiang, J. X.; Bonillo, B.; Ren, S.; Ratvijitvech, T.; Guiglion, P.; Zwijnenburg, M. A.; Adams, D. J.; Cooper, A. I. *J. Am. Chem. Soc.* **2015**, *137* (9), 3265–3270.

(16) Sprick, R. S.; Bonillo, B.; Clowes, R.; Guiglion, P.; Brownbill, N. J.; Slater, B. J.; Blanc, F.; Zwijnenburg, M. A.; Adams, D. J.; Cooper, A. I. *Angew. Chemie - Int. Ed.* **2016**, *55* (5), 1792–1796.

(17) Yang, C.; Ma, B. C.; Zhang, L.; Lin, S.; Ghasimi, S.; Landfester, K.; Zhang, K. A. I.; Wang, X. *Angew. Chemie - Int. Ed.* **2016**, *55* (32), 9202–9206.

(18) Woods, D. J.; Sprick, R. S.; Smith, C. L.; Cowan, A. J.; Cooper, A. I. *Adv. Energy Mater.* **2017**, *7* (22), 1–6.

(19) Xu, Y.; Mao, N.; Feng, S.; Zhang, C.; Wang, F.; Chen, Y.; Zeng, J.; Jiang, J. X. *Macromol. Chem. Phys.* **2017**, *218* (14), 1–9.
